# Synthesis of Sulfur Enriched Cyclohexenes via the Tungsten-Promoted Dearomatization of a Phenyl Sulfone

**DOI:** 10.1002/ejoc.202501090

**Published:** 2026-02-04

**Authors:** Daniel J. Siela, Matthew C. McGraw, Diane A. Dickie, W. Dean Harman

**Affiliations:** Department of Chemistry, University of Virginia, Charlottesville, Virginia, USA

**Keywords:** dearomatization, phenyl sulfones, sulfur addition reactions, synthetic methods, tungsten

## Abstract

Prior work with the dearomatization agent [WTp(NO)(PMe_3_)] (Tp = trispyrazolylborate) has demonstrated the ability to synthesize *cis*, *cis*–3,4,6 trisubstituted cyclohexenes from phenyl sulfones. Herein, the compatibility of these methods with sulfur nucleophiles was investigated for the synthesis of sp^3^-enriched thioether- and thioacetate-functionalized cyclohexenes. Protonation of the WTp(NO)(PMe_3_)(*η*^2^-PhSO_2_Me) complex followed by addition of a sulfur nucleophile was only successful for a thioacetate salt. However, when the first nucleophile was an ester enolate, subsequent protonation and nucleophilic addition successfully yielded a range of cyclohexene complexes with thioacetate, thiol, and sulfonyl substituents. Oxidative decomplexation of these complexes liberated the corresponding cyclohexene products. This strategy was then applied to the synthesis of a thioglycolate ester functionalized tetrahydrophenanthridinone.

## 1 ∣ Introduction

As the fifth-most common element in pharmaceuticals, sulfur continues to be a focal point for medicinal chemists, appearing in 20% of new FDA-approved drugs in 2024 [1, 2]. The sulfur atom’s high polarizability and ability to exist in different oxidation states provide versatility in drug design. Medicinal chemists can adjust the molecular structure around the thioether (e.g., a sulfoxide or sulfone) to modify a drug’s solubility and membrane permeability, which influences its absorption and distribution throughout the body [2]. Of the various sulfur-containing functional groups, thioethers rank as the third most prevalent amongst FDA-approved drugs [3]. Thioether-containing drugs include anti-cancer drugs (e.g., axitinib and trabectedin), anti-HIV drugs (tipranavir, darunavir), antipsychotics (e.g., chlorpromazine and thioridazine), and H2 antagonists (e.g., cimetidine and nizatidine) [3]. A number of important medicines feature a thioether connected to a chiral heterocyclic sp^3^ carbon, including the penicillin class of antibiotics, and the antitumor drug trabectedin [3]. The medicinal chemistry community has become increasingly aware of the strategic value of incorporating stereogenic sp^3^ carbon centers in small molecule design; such molecules offer a notable improvement to binding selectivity compared to their “flat” counterparts [4, 5]. Carbocyclic thioether pharmaceuticals are also known, such as lefamulin (novel antibiotic) or spironolactone (aldosterone antagonist), but examples of this architecture are far less common [6, 7].

**SCHEME 1 ∣:**
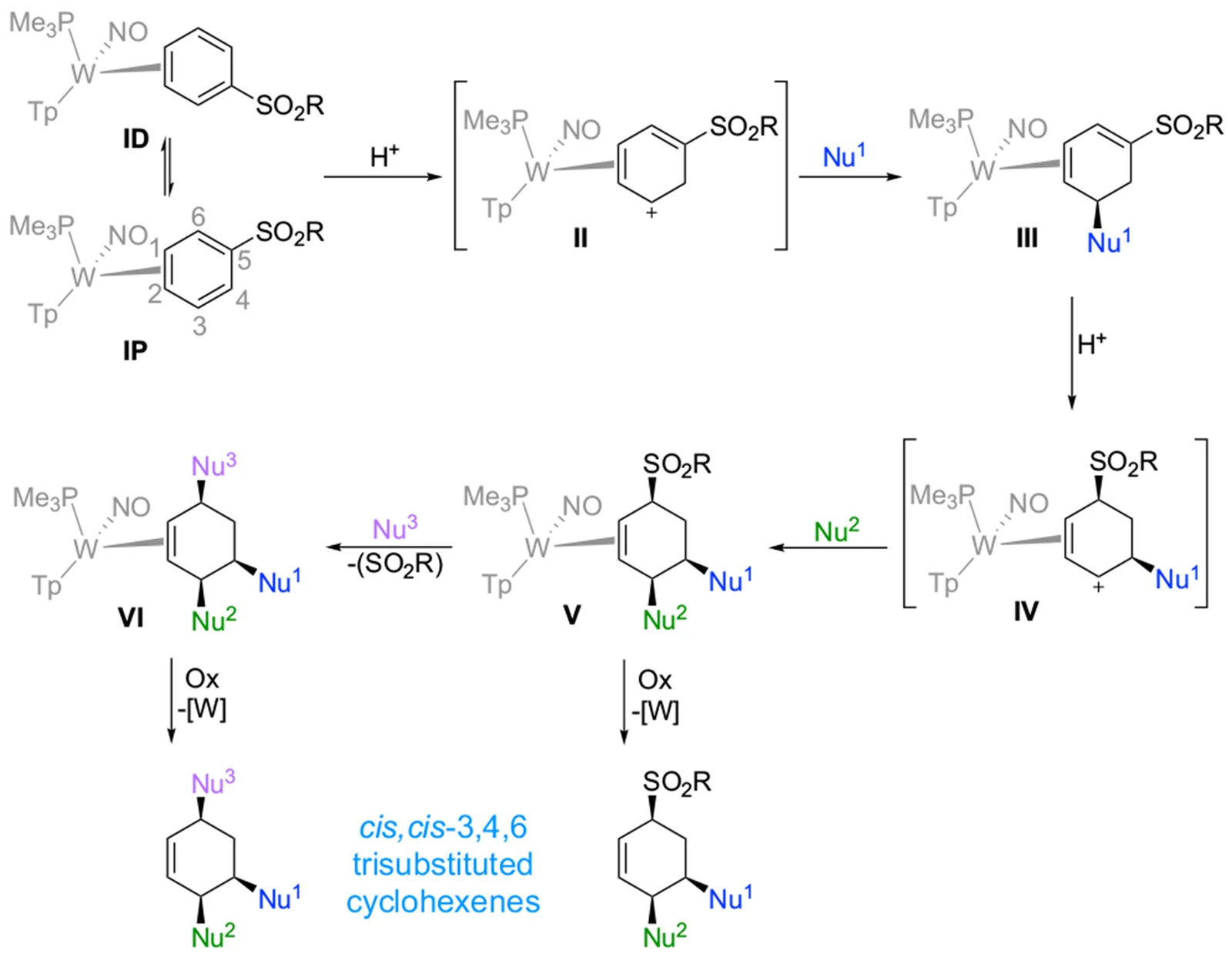
General synthetic strategy to *cis, cis*–3,4,6 trisubstituted cyclohexenes from WTp(NO)(PMe_3_)(*η*^2^-PhSO_2_R); Tp = trispyrazolylborate.

Previously, we have shown the ability to synthesize highly functionalized cyclohexenes from benzenes using the dearomatization agent [WTp(NO)(PMe_3_)] ([W]) [8]. Dihapto coordination of an arene to this tungsten complex activates the unbound portions of the arene to sequential electrophilic/nucleophilic addition reactions [9, 10]. Recent studies have demonstrated that complexes of the form WTp(NO)(PMe_3_)(*η*^2^-PhSO_2_R) are versatile precursors to *cis, cis*-3,4,6 trisubstituted cyclohexenes [9]. Hence, we chose the *η*^2^-phenylsulfone system as a foundation to explore possible new routes to sulfur-functionalized cyclohexenes for drug design. This general strategy for the synthesis of trisubstituted cyclohexenes is summarized in Scheme 1. Protonation of the *η*^2^-(methyl phenylsulfone) complex (where, R = Me) occurs selectively at C4 (*ortho* to the sulfonyl group) to form an arenium complex (**II**). At this point, the first nucleophile (Nu^1^) can be added to generate a diene complex (**III**). Subsequent protonation at C5 provides a *π*-allyl complex (**IV**), which then can be treated with a second nucleophile (Nu^2^) to form the sulfonyl-substituted cyclohexene complex **V**. Finally, replacement of the sulfinate by a third nucleophile (Nu^3^) can be carried out to form the trifunctionalized cyclohexene **VI**. While this reaction sequence has been carried out with a variety of nucleophiles, the use of sulfur nucleophiles in this scheme is largely unexplored [8, 10, 11]. In cases where a potential leaving group is located on an allylic carbon of **VI**, elimination to a *π*-allyl complex would be a major concern, and at the outset of this study, it was not clear whether sulfur substituents would be viable candidates for Nu^1^–Nu^3^ (Scheme 1) [12, 13].

## 2 ∣ Results and Discussion

### 2.1 ∣ First Nucleophilic Addition

**SCHEME 2 ∣:**
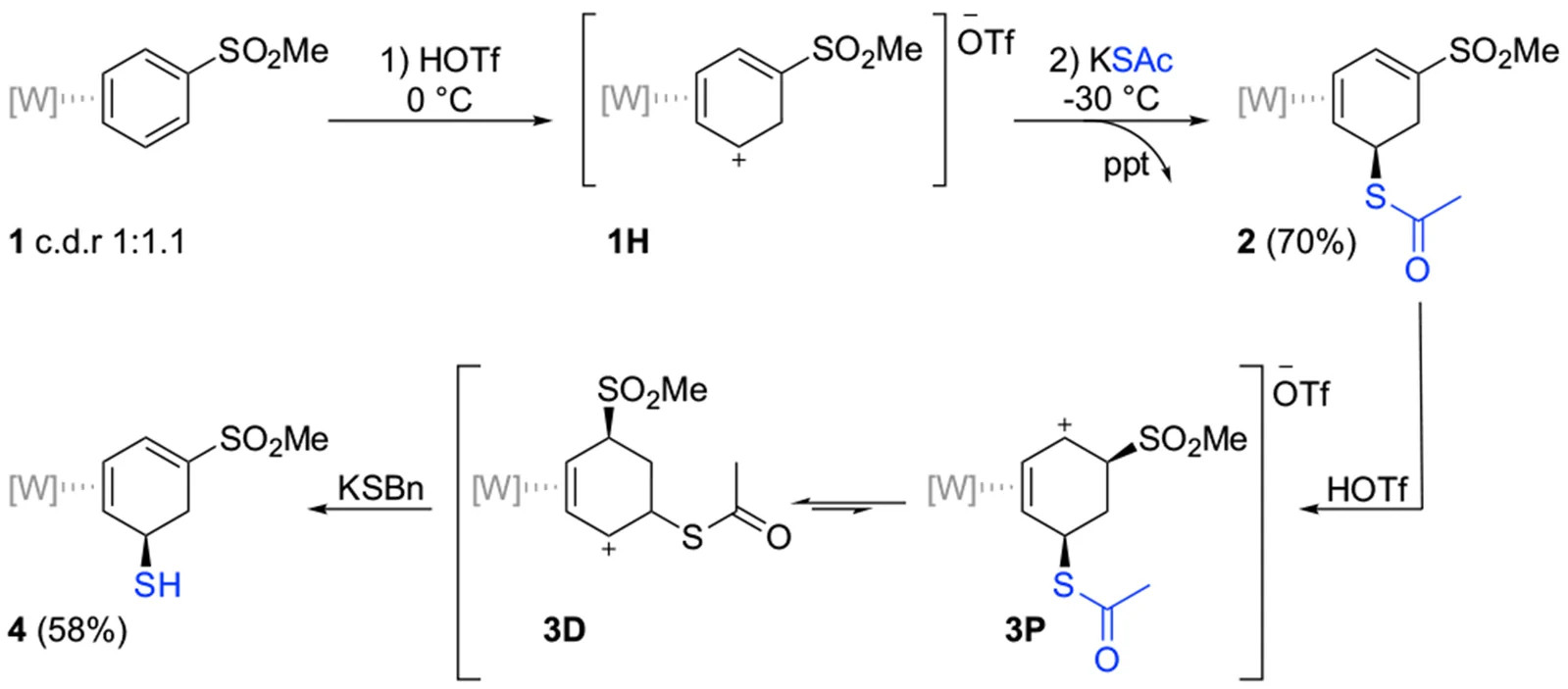
Preparation of sulfur-substituted *η*^2^-sulfonyl cyclohexadiene complexes. [W] = WTp(NO)(PMe_3_).

As previously described, the *η*^2^-phenylsulfone complex (**I**) initially exists as a roughly equal mixture of two coordination diastereomers (c.d.) differing by the relative position of the sulfone in relation to other metal-bound ligands (**IP** = sulfone proximal to the PMe_3_, **ID** = sulfone distal to the PMe_3_) [9]. However, through the initial protonation of the arene complex at C4, the ratio of these diastereomers is resolved to >20:1 for the first nucleophilic addition (Scheme 1) [9]. While the addition of irreversible nucleophiles (i.e., ester enolates; compound **5**) has been successfully carried out at C3 (Scheme 1), diene complexes of the form **III**, where Nu^1^ is a potential leaving group, tend to be shortlived as reversion to the arenium complex (**II**) ultimately leads to decomposition [9]. Consistent with this, sulfonyl diene complexes of type III (Scheme 1) were generally inaccessible using sulfur nucleophiles as Nu^1^. However, in the case of potassium thioacetate, compound **2** serendipitously precipitated from the crude reaction mixture at cold temperatures, allowing for its successful isolation, trapped in the solid state, which prevents subsequent decomposition. Characterization of **2** was performed via 2D NMR data and single-crystal X-ray diffraction (SC-XRD). A ^1^HNMR spectrum of compound **2** featured a dd at 7.69 ppm (H6), which has an NOE interaction with the PMe_3_. This signal is a clear diagnostic for diene complexes of type **III**. Additionally, ^13^C-NMR data include a peak at 196.7 ppm, indicating the presence of a thioacetate group. The SC-XRD molecular structure confirms that the addition of the thioacetate occurred anti- to the metal (Scheme 2), as is typically observed for reactions of **1H** with carbon nucleophiles [9]. Despite the presumed lability of the thioacetate group, protonation of **2** with strong acid at cold temperatures (<−30°C) generated a clean allyl species (**3**; existing as two distinct but rapidly interchanging conformers; Scheme 2) that could be observed by ^1^H-NMR in situ (SI) [12]. We posit that while protonation of the thioacetate moiety undoubtedly occurs under these highly acidic conditions, the cold temperatures slow the loss of the thioacetate group enough to allow for protonation of the diene complex **2**. Attempts to isolate or functionalize allyl complex **3** with various nucleophiles generally resulted in elimination back to the diene complex **2**. However, when **3** was treated with a benzyl mercaptan salt, the thioacetate group of **2** was converted to a sulfhydryl, resulting in diene complex **4**. Attempts to further functionalize **4** through the addition of acid led to decomposition. Dearomative methods yielding sulfur-substituted diene complexes such as **2** and **4** are relatively rare, with limited examples coming from more-traditional *η*^6^-coordination of arenes to manganese [14].

### 2.2 ∣ Second Nucleophilic Addition

**SCHEME 3 ∣:**
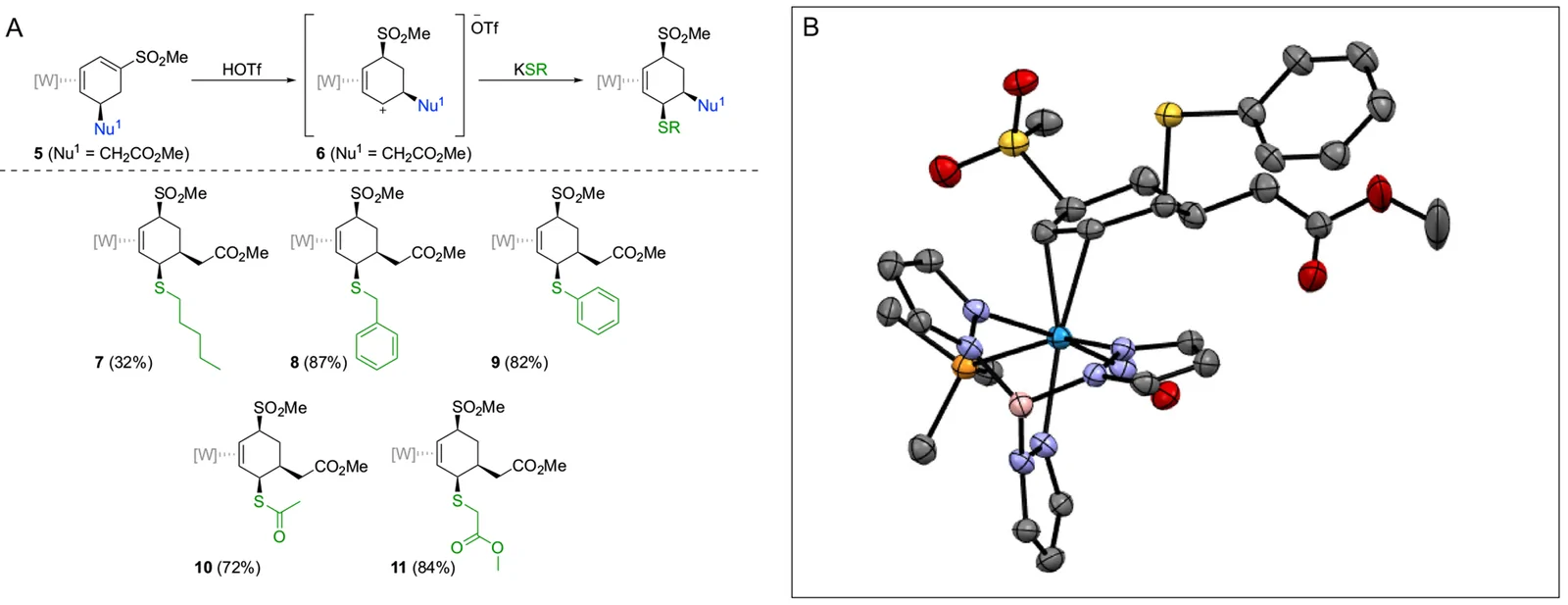
(A) Preparation of sulfur-substituted *η*^2^-sulfonyl cyclohexene complexes from *η*^2^-PhSO_2_Me. Yields are estimated based on mass recovery of the impure (^~^90%) compounds. (B) Crystal structure (SC-XRD) of **9** displayed at 50% ellipsoids.

Using sulfur nucleophiles for the first addition (Nu^1^ in Scheme 1) resulted in a diene complex (**III**) that was prone to eliminate back to the arene complex **I**. This retroaddition was suspected to be the result of residual protic sources from the generation of the arenium complex **1H** [9]. Unlike the arenium complex (**1H** in Scheme 2), allyl complexes resulting from the protonation of *η*^2^-cyclohexadienes (**6**) are isolable as triflate salts and are stable as solids for extended periods under nitrogen atmosphere at −20°C (Scheme 3). Further, such *π*-allyl complexes tend to be slow to eliminate in solution at cold temperatures. Thus, a diene complex **5** was prepared from **1** and an ester enolate, and this complex was then protonated to form *π*-allyl complex **6**. By combining **6** with anionic sulfur nucleophiles (Nu^2^; prepared as sodium or potassium salts), we were able to isolate sulfonyl cyclohexene complexes **7–11** in moderate yields (Scheme 3). Reaction monitoring via ^1^H-NMR spectroscopy showed that while addition of Nu^2^ occurred cleanly under these conditions, warming the reaction to room temperature for extended periods or exposure to water, such as in a liquid–liquid extraction procedure, caused significant elimination of the sulfur moiety. As such, **7–11** were isolated without aqueous work-up, instead separating the product from residual salts by precipitating the crude reaction mixture into pentane or hexanes collecting the solids on a fritted disc, and washing any additional product back into the filtrate with DCM. The combined filtrates were then evaporated to dryness under reduced pressure to collect the complex without further purification. 2D-NMR data were used to determine the connectivity of **7–10**, and SC-XRD (**8–10**) data confirmed the *cis, cis*-stereochemistry in which the second nucleophile adds to the ring-face opposite to that of tungsten coordination. When we attempted the synthesis of **8** in one pot from **5** with BnSK, a spectroscopically distinct product was isolated (**13**), where the sulfone had been replaced by a second equivalent of -SBn. This nucleophilic displacement of the sulfone group, which has been previously observed, offers an additional site to add sulfur nucleophiles [9].

### 2.3 ∣ Third Nucleophilic Addition

**SCHEME 4 ∣:**
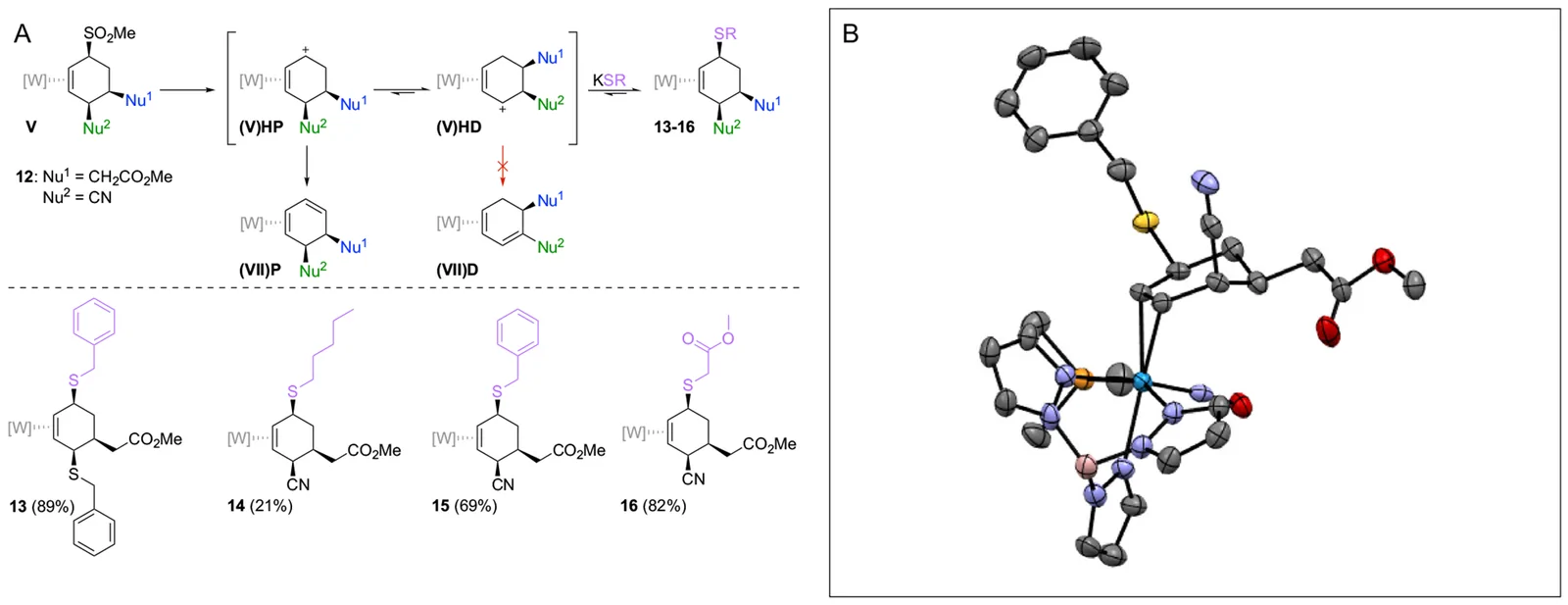
(A) Proposed mechanism and side products of sulfone displacement by thiolates for the preparation of sulfur-substituted *η*^2^-cyclohexene complexes. (B) Crystal structure (SC-XRD) of **15** displayed at 50% ellipsoids.

Optimization of sulfone displacement using the sulfonyl cyclohexene complex, **12** (Nu^1^ = CH_2_CO_2_Me; Nu^2^ = CN), required a mild Lewis acid (i.e., methanol or silica) to promote elimination of sulfinic acid, generating the *π*-allyl complex intermediate (**(V)HD**; Scheme 4). In the presence of excess nucleophile, **(V)HD** rapidly undergoes addition to the more kinetically accessible proximal allyl, **(V)HP**. However, due to the lability of these allylic thiol substituents, elimination to a diene ((**VII)P**) occurs over time (NMR). It should be noted that the elimination to (**VII)D**, although possible, was not observed. Presumably this is because the endo-oriented proton at the homoallylic carbon of **(V)HD** is kinetically less accessible than the exo-oriented proton of **(V)HP**. This predisposition of the allyl mercaptan complexes **7–11** and **13–16** to convert to diene (**VII)P** made their purification beyond 90% difficult. As such, complexes **7–11** and **13–16** were characterized and carried forward without further purification. Key spectroscopic features include a signal for H6 shifted upfield from that of **12**, with a strong NOE interaction to the PMe_3_, and HMBC interactions across the thioether (C15 for **13**; C11 for **14–16** (see SI)). SC-XRD analysis of **14–16** confirms their structures in which the addition of the thiols occurs anti to the bulky tungsten complex [9]. This antiaddition pattern explains the *cis, cis*-stereochemistry observed for the organic ligands of **7–16**.

### 2.4 ∣ Liberation of Sulfur-Substituted Cyclohexenes

**SCHEME 5 ∣:**
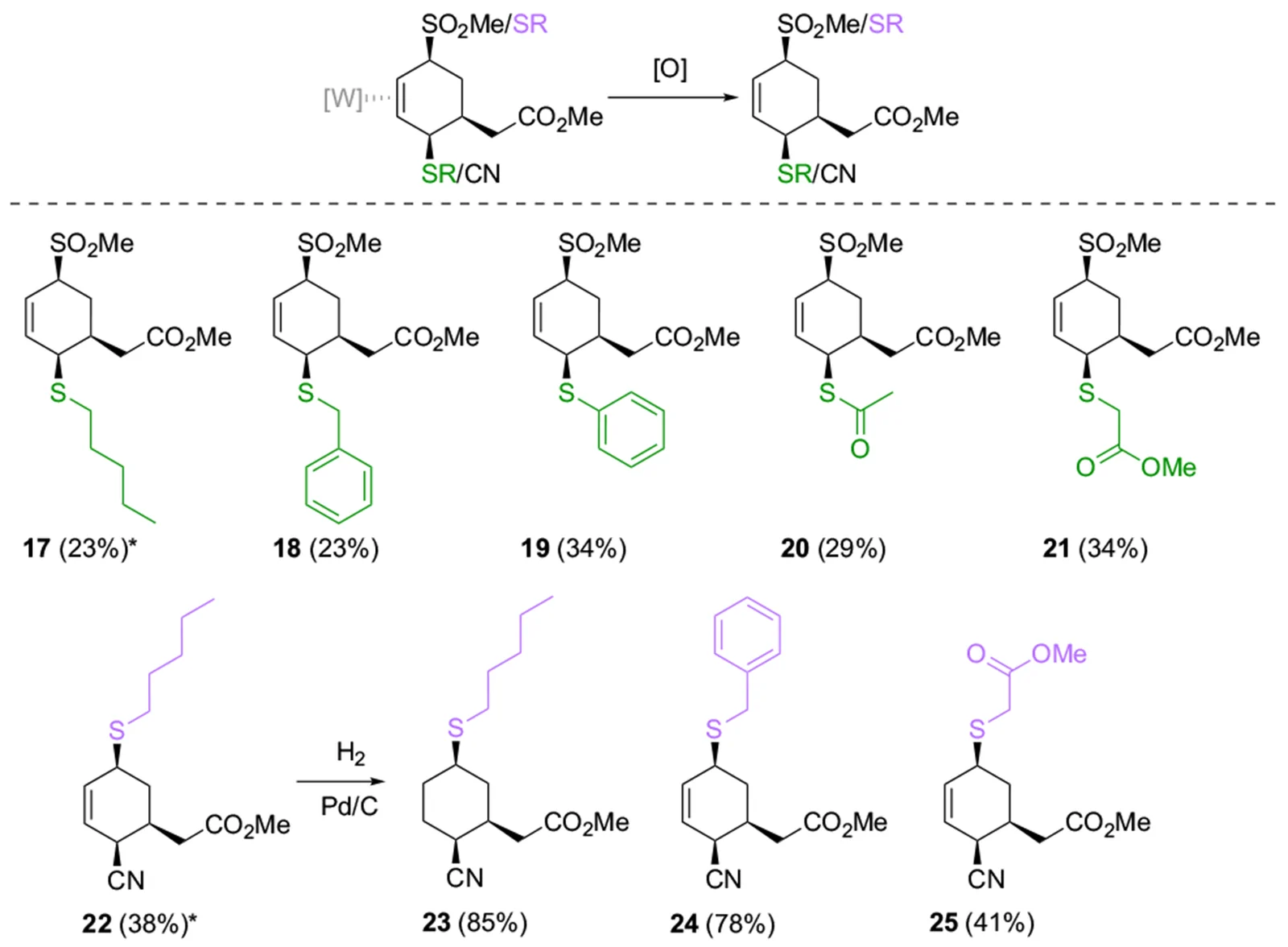
Liberation of sulfur-functionalized *cis, cis*–3,4,6 trisubstituted cyclohexenes. * Yield calculated over 2 steps from **6** for **17** and from **12** for **22**.

To liberate the sulfur-substituted *cis, cis*-3,4,6 trisubstituted cyclohexene ligands from the [WTp(NO)(PMe_3_)] fragment, oxidation of the metal via either 2,3-dichloro-5,6-dicyano-1,4-benzoquinone (DDQ) or ceric ammonium nitrate (CAN) was employed. An initial screening (NMR scale) of oxidants found CAN or DDQ to be superior to other mild oxidants such as NOPF_6_ or AgNO_3_. While oxidation of the thioether was a concern, maintaining strict equivalents of oxidant avoided the formation of sulfoxide/sulfone. Additionally, sodium sulfite was employed to quench excess oxidant in some cases. After purification, compounds **17–22**, **24**, and **25** were isolated in yields from 23% to 78% (Scheme 5). Strict equivalences for these oxidants and short reaction times were paramount to prevent side reactions with the liberated organic, and cold reaction temperatures were often employed to prevent substituent elimination prior to oxidation. As purification of the preceding allylic thiol complexes (**7–11**, **14–16**) was challenging, we investigated liberating cyclohexenes **17** and **22** directly without purification of the corresponding complexes (**7** and **14**, respectively). From this approach, we noticed a significant improvement in the overall yield of **17** and **22** over two steps. Additionally, to demonstrate the ability to derive cyclohexanes from these methods, compound **23** was obtained via hydrogenation of **22**. Compounds **17, 18, 20**, and **22–25** were characterized by 2D NMR data, and **17–25** were analyzed by HRMS to ensure the identity of the organics post liberation. Yields of the final organics post purification and isolation were generally modest (23–78%), owing in part to elimination of the sulfur-functionality to a diene complex prior to oxidative liberation of the cyclohexene.

The advantage of sulfur additions to WTp(NO)(PMe_3_)(*η*^2^-PhSO_2_R) compared to other routes to similar sulfur-containing cylcohexenes is the excellent regio- and stereoselectivity offered by the metal. Compared to Diels-Alder reactions with sulfur-substituted dienes, the relative position of the sulfur substituent via these methods is not limited by the electronics of the other ring substituents (cf. **17** vs. **22**) [15]. As previously described, the steric bulk of the metal complex directs additions to the bound ring with high stereoselectivity relative to the tungsten stereocenter. In addition, we note that the ability to enantioenrich the WTp(NO)(PMe_3_)(*η*^2^-arene) complexes would provide a means to isolate single enantiomers of these organics without other chiral directing groups, although this has not been explored here [16, 17]. Although most of the organic products obtained herein are derived from the *η*^2^-sulfone and a silyl ketene acetal or cyanide as one of the first two nucleophiles, we expect similar reactivity patterns using other established nucleophilic addition products from the *η*^2^-sulfone system, including the use of hydride, amides (e.g., 2-azetidinone), and organocuprates as Nu^1^ or Nu^2^ [9, 18].

### 2.5 ∣ Extensions

**SCHEME 6 ∣:**
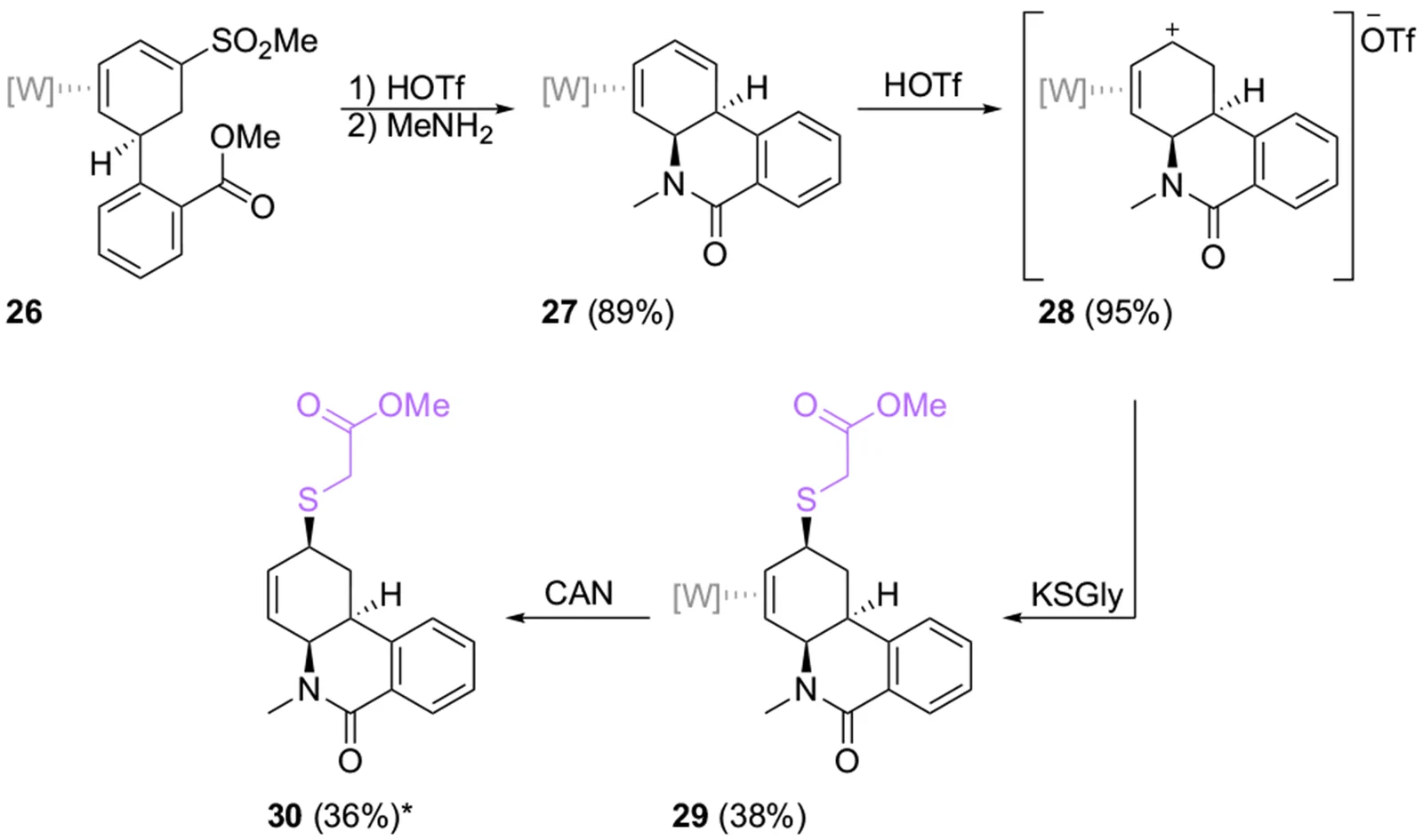
Synthesis of sulfur-substituted tetrahydrophenanthridi-none. * Yield calculated over 2 steps from **28**.

While compounds **17–25** represent only a modest range of sulfur-substituted products, the insight gained into how such thiol and thioacetate nucleophiles behave with the WTp(NO)(PMe_3_) (*η*^2^-PhSO_2_R) system can be applied to more complex structures. As previously described, protonation of an *η*^2^-sulfonyl cyclohexadiene resulting from a benzoate addition (**26**), followed by the addition of a primary amine (Nu^2^) to form a complex of type **V** (Scheme 1), leads to intramolecular lactamization. Subsequent loss of the sulfone results in an *η*^2^-dihydrophenanthridinone (**27**; Scheme 6) [13, 19]. This diene can then be protonated to a stable *π*-allyl intermediate, **28**, of type **(V)H** (Scheme 4). Analogous to **6, 28** reacts cleanly with potassium thioglycolate (KSGly) to afford the sulfur-substituted tetrahydrophenanthridi-none **29**. The organic phenanthridinone derivative, **30**, was isolated via oxidative decomplexation with CAN. Given the continued pharmaceutical relevance of phenanthridinones, incorporation of a thioether, as with **30**, demonstrates the utility of the dearomatization process described herein [20].

## 3 ∣ Conclusion

We have explored the transformation of a phenylsulfone into sp^3^-enriched thioether and thioacetate functionalized cyclohexenes. This transformation occurs through the *η*^2^-coordination of the arene to a [WTp(NO)(PMe_3_)] fragment. Initial protonation/nucleophilic addition to WTp(NO)(PMe_3_)(*η*^2^-PhSO_2_Me) leads to sulfur-functionalized *η*^2^-cyclohexadienes; however, further functionalization of these dienes was unsuccessful. Despite this, the addition of various thiol and thioacetate salts to other *η*^2^-allyl complexes did result in isolable trisubstituted cyclohexene complexes containing both an allylic sulfone and a thioether or thioacetate moiety, respectively. Substitution of the sulfone group from such complexes by thiolate or thioacetate salts was also successful. Oxidative decomplexation via DDQ or CAN provided a small library of *cis, cis*–3,4,6 trisubstituted cyclohexenes with sulfur functionality in the 3- and/or 6-positions. The palladium-catalyzed hydrogenation of one such example to its corresponding cyclohexane was also demonstrated. The insights provided by these methods were then applied to access a sulfur-functionalized tetra-hydrophenanthridinone. This approach provides stereo- and regiocontrol without the need for prefunctionalization (e.g., halides) or precious metals. Furthermore, the carbocyclic frameworks accessed herein hold potential for use as drug discovery fragments since the incorporation of both sp^3^ centers and sulfur moieties are desirable features in pharmaceutical development.

## Supplementary Material

Supporting Info final

Additional **supporting information** can be found online in the Supporting Information section. **Supporting Fig. S1**: Key NOE interactions (blue dashes) between the [WTp(NO)(PMe_3_)] fragment and the *η*^2^ -bound ligand. **Supporting Fig. S2**: ^1^H NMR ((CD_3_)_2_CO) of Compound **2**. **Supporting Fig. S3**: ^13^C NMR ((CD_3_)_2_CO) of Compound **2**. **Supporting Fig. S4**: ^1^H NMR (CD_3_CN) of Compound **3**. Unknown decomposition peaks can be seen growing in ~6.3 – 8.4 ppm and 1.29 ppm as the sample warms above −30°C. **Supporting Fig. S5**: ^1^H NMR ((CD_3_)_2_CO) of Compound **4**. **Supporting Fig. S6**: ^13^C NMR ((CD_3_)_2_CO) of Compound **4**. **Supporting Fig. S7**: ^1^H NMR (CD_3_Cl) of Compound **7**. **Supporting Fig. S8**: ^13^C NMR (CD_3_Cl) of Compound **7**. **Supporting Fig. S9**: ^1^H NMR (CD_3_Cl) of Compound **8**. **Supporting Fig. S10**: ^13^C NMR (CD_3_Cl) of Compound **8**. **Supporting Fig. S11**: ^1^H NMR ((CD_3_)_2_CO) of Compound **9**. **Supporting Fig. S12**: ^13^C NMR ((CD_3_)_2_CO) of Compound **9**. **Supporting Fig. S13**: ^1^H NMR (CD_3_CN) of Compound **10** (with ~0.15 residual Compound **5**). **Supporting Fig. S14**: ^13^C NMR (CD_3_CN) of Compound **10** (with ~0.15 residual Compound **5**). **Supporting Fig. S15**: ^1^H NMR (CD_3_Cl) of Compound **11**. **Supporting Fig. S16**: ^13^C NMR (CD_3_Cl) of Compound **11**. **Supporting Fig. S17**: ^1^H NMR ((CD_3_)_2_CO) of Compound **13**. **Supporting Fig. S18**: ^13^C NMR ((CD_3_)_2_CO) of Compound **13**. **Supporting Fig. S19**: ^1^H NMR (CD_3_Cl) of Compound **14**. **Supporting Fig. S20**: ^1^H NMR (CD_3_Cl) of Compound **14**. **Supporting Fig. S21**: ^1^H NMR ((CD_3_)_2_CO) of Compound **15**. **Supporting Fig. S22**: 3C NMR ((CD_3_)_2_CO) of Compound **15**. **Supporting Fig. S23**: ^1^H NMR (CDCL_3_) of Compound **16**. **Supporting Fig. S24**: ^13^C NMR (CDCL_3_) of Compound **16**. **Supporting Fig. S25**: ^1^H NMR (CD_3_CN) of Compound **17**. **Supporting Fig. S26**: ^13^C NMR (CD_3_CN) of Compound **17**. **Supporting Fig. S27**: ^1^H NMR (CDCL_3_) of Compound **18**. **Supporting Fig. S28**: ^13^C NMR (CDCL_3_) of Compound **18**. **Supporting Fig. S29**: ^1^H NMR (CDCL_3_) of Compound **19**. **Supporting Fig. S30**: ^13^C NMR (CDCL_3_) of Compound **19**. **Supporting Fig. S31**: ^1^H NMR (CD_3_CN) of Compound **20**. **Supporting Fig. S32**: ^13^C NMR (CD_3_CN) of Compound **20**. **Supporting Fig. S33**: ^1^H NMR (CDCL_3_) of Compound **21**. **Supporting Fig. S34**: ^13^C NMR (CDCL_3_) of Compound **21**. **Supporting Fig. S35**: ^1^H NMR (CD_3_CN) of Compound **22**. **Supporting Fig. S36**: ^13^C NMR (CD_3_CN) of Compound **22**. **Supporting Fig. S37**: ^1^H NMR (CD_3_CN) of Compound **23**. **Supporting Fig. S38**: ^13^C NMR (CD_3_CN) of Compound **23**. **Supporting Fig. S39**: ^1^H NMR ((CD_3_)_2_CO) of Compound **24**. **Supporting Fig. S40**: ^13^C NMR ((CD_3_)_2_CO) of Compound **24**. **Supporting Fig. S41**: ^1^H NMR (CD_3_CN) of Compound **25**. **Supporting Fig. S42**: ^13^C NMR (CD_3_CN) of Compound **25**. **Supporting Fig. S43**: ^1^H NMR (CD_3_CN) of Compound **28**. **Supporting Fig. S44**: ^1^H NMR (CD_2_Cl_2_) of Compound **29**. **Supporting Fig. S45**: ^13^C NMR (CD_2_Cl_2_) of Compound **29**. **Supporting Fig. S46**: ^1^H NMR (CD_3_CN) of Compound **30**. **Supporting Fig. S47**: ^13^C NMR (CD_3_CN) of Compound **30**. CCDC deposition numbers 2492652 – 2492658 contain the supplementary crystallographic data for this paper. These data can be obtained free of charge from The Cambridge Crystallographic Data Centre via www.ccdc.cam.ac.uk/structures.

## Data Availability

The data that support the findings of this study are available from the corresponding author upon reasonable request.
