# Supplementary material for: Synthesis of Sulfur Enriched Cyclohexenes via the Tungsten-Promoted Dearomatization of a Phenyl Sulfone
Source: European J Org Chem. Author manuscript; Available in PMC 2026 Sep 25. (PMC13604020; doi:10.1002/ejoc.202501090)
Supplement: Supplementary file 1 [file NIHMS2209200-supplement-Supporting_Info_final.pdf]

Synthesis of Sulfur Enriched Cyclohexenes via the Tungsten-Promoted Dearomatization of a Phenyl Sulfone

Daniel J. Siela[1], Matthew C. McGraw[1], Diane A. Dickie[1], and W. Dean Harman*[1]

[1] Daniel J. Siela, Matthew C. McGraw, Diane A. Dickie, and W. Dean Harman

Department of Chemistry, University of Virginia; Charlottesville, VA 22904 U.S.A.
E-mail: wdh5z@virginia.edu

Supplementary Information

Table of Contents

Supplementary Information **1**

Table of Contents 1

Experimental **2**

NMR Spectra **18**

Crystallographic Data **63**

References **66**

Chemical Shifts are reported in ppm, and coupling constants (J) are reported in hertz (Hz). All synthetic reactions were performed in a glovebox under a dry nitrogen atmosphere unless otherwise noted. All solvents were purged with nitrogen prior to use. Deuterated solvents were used as received from Cambridge Isotopes and were purged with nitrogen under an inert atmosphere. When possible, pyrazole protons of the tris(pyrazolyl)borate (Tp) ligand were uniquely assigned (e.g., “Tp3B”) using two-dimensional NMR data (see Supplementary Figure 1). If unambiguous assignments were not possible, protons were labeled as “H3 or H5, or H3/5”. All J values for Tp protons are 2(±0.4) Hz. BH peaks (around 4−5 ppm) in the 1 H NMR spectra are not assigned due to their quadrupole broadening. Compound 1, 5, 6, 12, 26, and 27 were previously reported.[9, 19] Full characterization of compounds is provided in the SI.

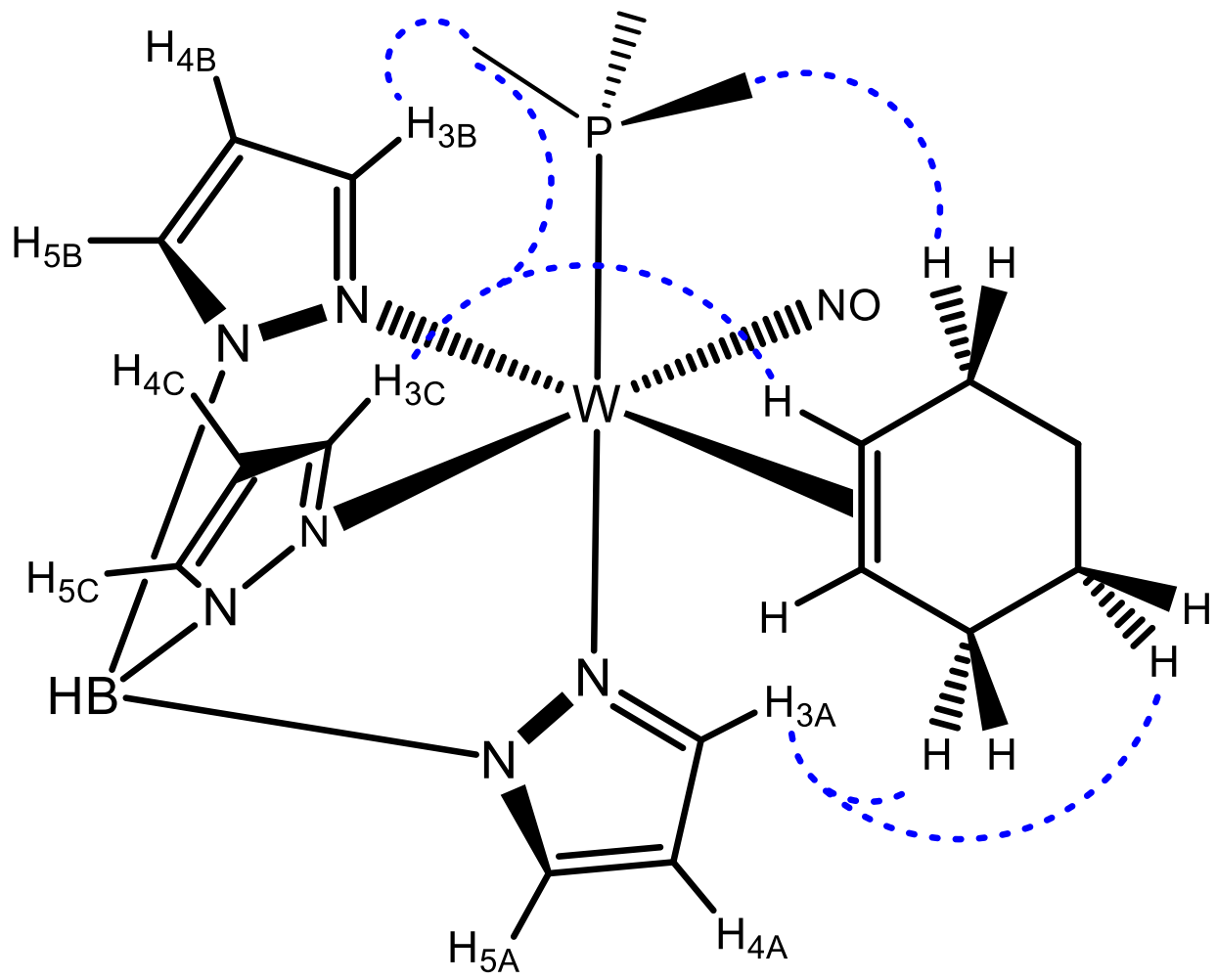

Supplementary Figure 1. Key NOE interactions (blue dashes) between the [WTp(NO)(PMe_3)] fragment and the η²-bound ligand.

Experimental

Compound 2

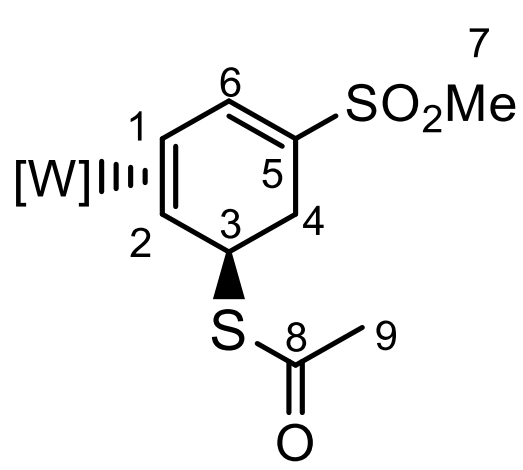

Compound **1** (100 mg; 0.152 mmol) was cooled to 0°C, followed by the addition of 1M HOTf in MeCN (228 µL; 0.228 mmol) cooled to -30°C. The reaction was allowed to stir until a yellow precipitate formed (~15 min) and was then cooled to -30°C. Meanwhile, KSAc (69 mg; 0.607 mmol) was taken in MeOH (2 mL), cooled to -30°C, and then added to the allyl complex. The reaction was allowed to stir cold for 18 hrs, during which significant white precipitate formed. Saturated $NaHCO_3$ solution (1 mL) was added to induce further precipitation, and the crude mixture was filtered through a fine porosity fritted disc. The precipitate was washed with pentane (3x3 mL), yielding Compound **2** as a white solid (56 mg; 0.076 mmol; 50%).

^{1}H NMR (800 MHz, Acetone) δ 8.15 (1H, d, *J* = 2.0 Hz, Tp3B), 8.08 (1H, d, *J* = 2.0 Hz, Tp3A), 8.00 (2H, dd, *J* = 3.4, 2.4 Hz, Tp5B/Tp5C), 7.88 (1H, d, *J* = 2.4 Hz, Tp5A), 7.69 (1H, dd, *J* = 5.8, 2.5 Hz, H6), 7.67 (1H, d, *J* = 2.2 Hz, Tp3C), 6.44 (1H, t, *J* = 2.2 Hz, Tp4B), 6.41 (1H, t, *J* = 2.2 Hz, Tp4A), 6.37 (1H, t, *J* = 2.2 Hz, Tp4C), 5.09 (1H, dq, *J* = 5.9, 2.0 Hz, H3), 3.14 (1H, m, H4), 3.04 (1H, ddd, *J* = 12.0, 9.0, 5.8 Hz, H1), 2.89 (3H, s, H7), 2.71 (1H, d, *J* = 16.8 Hz, H4), 2.19 (3H, s, H9), 1.47 (1H, d, *J* = 9.1 Hz, H2), 1.34 (9H, d, *J* = 8.7 Hz, PMe_3).

^{13}C NMR (201 MHz, Acetone) δ 196.7 (1C, C8), 145.1 (1C, C6), 144.6 (1C, Tp3B), 142.8 (1C, Tp3A), 142.1 (1C, Tp3C), 138.1 (1C, Tp5C), 137.6 (1C, Tp5B), 137.1 (1C, Tp5A), 127.3 (1C, C5), 107.6 (1C,

Tp4B), 107.4 (1C, Tp4C), 107.0 (1C, Tp4A), 59.7 (1C, C2), 48.1 (1C, d, *J* = 9.1 Hz, C1), 47.3 (1C, C3), 45.3 (1C, C7), 30.8 (1C, C9), 30.2 (1C, C4), 13.6 (3C,d, *J* = 29.1 Hz, PMe_3).

Compound 4

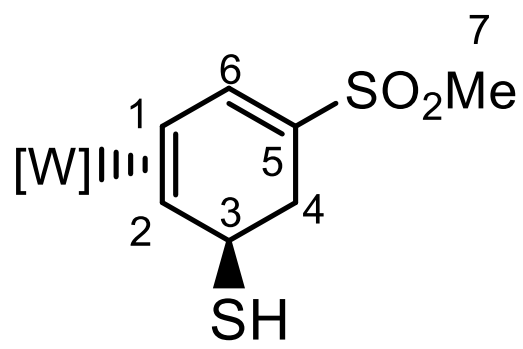

Compound **2** (80 mg; 0.109 mmol) was taken in MeCN (1.5 mL) and cooled to -30°C. To this, 1M HOTf in MeCN (163 µL; 0.163 mmol) cooled to -30°C was added and the reaction stirred for 15 min. Separately, BnSH (40 mg; 0.326 mmol) was added to a solution of 20%wt KOtBu (122 mg; 0.218 mmol) and cooled to -30°C. The allyl solution was then added to the thiol salt and stirred cold for 1.5 hr, during which white precipitate formed. Saturated $NaHCO_3$ solution (1 mL) was added to induce further precipitation, and the crude mixture was filtered through a fine porosity fritted disc. The precipitate was washed with hexane (2x2 mL), yielding Compound **4** as a white solid (44 mg; 0.063 mmol; 58%).

^{1}H NMR (800 MHz, Acetone) δ 8.12 (1H, d, *J* = 2.1 Hz, Tp3B), 8.02 (1H, d, *J* = 2.0 Hz, Tp3A), 8.00 (1H, d, *J* = 2.3 Hz, Tp5C), 7.99 (1H, d, *J* = 2.4 Hz, 1H), 7.87 (1H, d, *J* = 2.4 Hz, Tp5A), 7.72 (1H, dd, *J* = 5.8, 2.5 Hz, H6), 7.69 (1H, d, *J* = 2.2 Hz, Tp3C), 6.42 (1H, t, *J* = 2.2 Hz, Tp4B), 6.39 (1H, t, *J* = 2.2 Hz, Tp4C), 6.35 (1H, t, *J* = 2.2 Hz, Tp4A), 4.46 (1H, s, 3), 3.14 (1H, m, H4), 3.05 (1H, ddd, *J* = 12.0, 9.1, 5.6 Hz, H1), 2.90 (3H, s, H7), 2.70 (1H, m, H4), 1.62 (1H, d, *J* = 9.3 Hz, H2), 1.35 (9H, d, *J* = 8.6 Hz, PMe_3). **^{13}C NMR (201 MHz, Acetone) δ** 144.6 (2C, C6/Tp3B), 142.7 (1C, Tp3A), 142.1 (1C, Tp3C), 138.0 (1C, Tp5C), 137.6 (1C, Tp5B), 137.0 (1C, Tp5A), 126.4 (1C, C5), 107.5 (1C, Tp4B), 107.4 (1C, Tp4C), 107.0 (1C, Tp4A), 65.1 (1C, C2), 47.0 (1C, C1), 45.2 (1C, C7), 41.2 (1C, C3), 33.2 (1C, C4), 13.7 (3C, PMe_3). **HRMS (APCI$^+$):** $[M\text{-}SH]^+$ calcd. for $C_{19}H_{28}BN_7O_3PSW$, 660.1314; found, 660.1340.

Compound 7

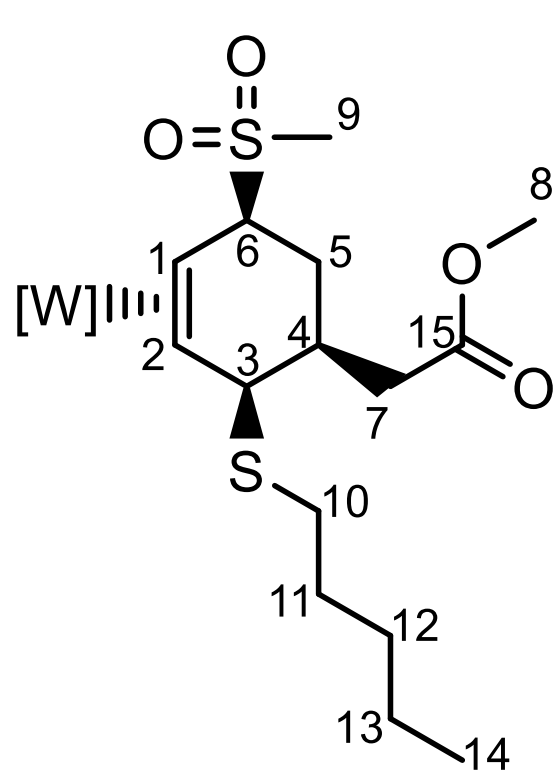

Compound **6** (30 mg; 0.034 mmol) was taken in DCM (1 mL) and cooled to -30°C. $KS(C_5H_{11})$ (5 mg; 0.035 mmol) was taken separately in THF (2 mL) and cooled to -30°C. The solution of **6** was then added to the thiol salt and stirred cold for 18 hrs. The crude reaction mixture was then precipitated into stirring pentane (20 mL) and filtered through a fine porosity fritted disc. The filtrate was evaporated to dryness in a tared vial to yield Compound **7** as a yellow oil (9 mg; 0.011 mmol; 32%).

^{1}H NMR (800 MHz, CDCl3) δ 8.24 (1H, d, *J* = 2.1 Hz,Tp3a), 8.09 (1H, d, *J* = 2.1 Hz, Tp3b), 7.73 (1H, d, *J* = 2.2 Hz, Tp3c), 7.70 (1H, d, J=2.1 Hz, Tp5b), 7.61 (1H, d, *J* = 2.3 Hz, Tp5a), 7.26 (1H, d, *J* = 2.2 Hz, Tp3c), 6.28 (1H, t, *J* = 2.1 Hz, Tp4b), 6.24 (1H, t, *J* = 2.2 Hz, Tp4c), 6.23(1H, t, J =2.1 Hz, Tp4a), 4.47 (1H, t, *J* = 9.5 Hz, H6), 4.01 (1H, s, H3), 3.70 (3H, s, H9), 3.10 (3H, s, H10), 2.94 (1H, ddt, *J* = 13.2, 11.1, 1.9 Hz, H1), 2.82 (1H, ddd, *J* = 16.7, 10.1, 2.1 Hz, H7), 2.44 (1H, ddd, *J* = 16.7, 4.1, 2.1 Hz, H7), 2.34 (2H, ddd, *J* = 10.8, 6.1, 2.4 Hz, H11), 1.89 (1H, ddd, *J* = 11.3, 8.3, 4.3 Hz, H5), 1.76 (1H, tdd, *J* = 12.9, 10.3, 2.1 Hz, H5), 1.60 (1H, m, H1), 1.26 (11H, m, PMe3 & H12), 1.10 (4H, m, H13 & H14), 0.72 (3H, td, *J* = 7.1, 2.2 Hz, H15).**^{13}C NMR (201 MHz, CDCl3) δ** 173.3 (1C, C8), 144.6 (1C, Tp3a), 144.1 (1C, Tp3b), 140.7 (1C, Tp3c), 136.6 (1C, Tp5c), 136.4 (1C, Tp5b), 136.1 (1C, Tp5a), 106.5 (1C, Tp4b), 106.1 (1C, Tp4c), 106.1 (1C, Tp5a), 63.7 (1C, C6), 61.7 (1C, C2), 51.6 (1C, C9), 51.4 (1C, C3), 39.2 (1C, C7), 38.8 (1C, d, *J* = 10.8 Hz, C1), 35.8 (1C, C10), 33.5 (1C, C11), 31.2 (1C, C13), 30.5 (1C, C4), 29.9 (1C, C12), 27.6 (C5), 22.3 (1C, C14), 14.0 (1C, C15), 13.8 (3C, d, *J* = 28.0 Hz, PMe3). **HRMS (APCI$^+$):** [M-S(C_5H_{11})]$^+$ calcd. for $C_{22}H_{34}BN_7O_5PSW$, 734.1682; found, 734.1687.

Compound 8

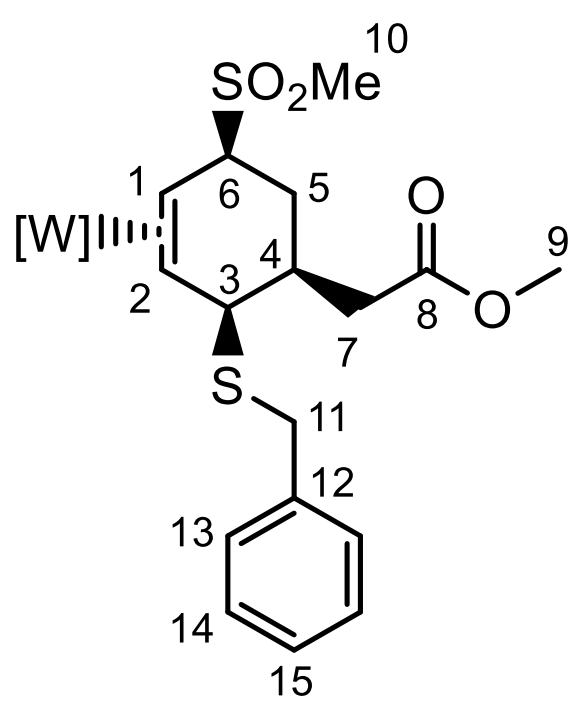

Compound **6** (100 mg; 0.113 mmol) was taken in DCM (3 mL) and cooled to -30°C. KSBn was taken separately in THF (3 mL) and cooled to -30°C, before being added to **6** and stirred 1 hr. The crude reaction mixture was then precipitated directly into stirring pentane (40 mL), and filtered through a fine porosity fritted disc. The precipitate was washed with DCM (2x3 mL) and the combined filtrate was evaporated to dryness in a tared vial to yield compound **8** as a solid (84 mg; 0.098 mmol: 87%).

^{1}H NMR (800 MHz, $CDCl_3$) δ 8.10 (1H, d, *J* = 2.1 Hz, Tp3A), 8.06 (1H, d, *J* = 2.0 Hz, Tp3B), 7.75 (1H, d, *J* = 2.3 Hz, Tp5C), 7.70 (1H, d, *J* = 2.4 Hz, Tp5B), 7.64 (1H, d, *J* = 2.4 Hz, Tp5A), 7.16 (1H, d, J= 2.1 Hz, Tp3C) 6.98 (3H, m, H14/H15), 6.88 (2H, m, H13), 6.27 (1H, t, *J* = 2.2 Hz, Tp4B), 6.24 (1H, t, *J* = 2.2 Hz, Tp4C), 6.21 (1H, t, *J* = 2.3 Hz, Tp4C), 4.47 (1H, t, *J* = 9.4 Hz, H6), 3.94 (1H, s, H3), 3.61 (3H, s, H9), 3.58 (1H, d, *J* = 13.3 Hz, H11), 3.53 (1H, d, *J* = 13.5 Hz, H11), 3.07 (1H, s, H10), 3.04 (1H, m, H4), 2.91 (1H, ddd, *J* = 13.1, 11.0, 1.5 Hz, H1), 2.76 (1H, dd, *J* = 16.5, 10.0 Hz, H7), 2.41 (1H, dd, *J* = 16.5, 4.2 Hz, H7), 1.91 (1H, m, H5), 1.69 (1H, td, *J* = 13.2, 10.3 Hz, H5), 1.50 (1H, d, *J* = 10.9 Hz, H2), 1.24 (9H, d, *J* = 8.5 Hz, PMe_3). **^{13}C NMR (201 MHz, $CDCl_3$) δ** 173.0 (1C, C8), 144.8 (1C, Tp3A), 144.0 (1C,Tp3B), 140.6 (1C,Tp3C), 139.4 (1C, C12), 136.5 (1C, Tp5C), 136.3 (1C, Tp5B), 136.0 (1C, Tp5A), 128.7 (1C, C13), 128.2 (2C, C14), 126.5 (1C, C15), 106.5 (1C, Tp4B), 106.3 (1C, Tp4C), 106.2 (1C, Tp4A), 63.5 (1C, C6), 61.5 (1C, C2), 51.7 (1C, C3), 51.5 (1C, C9), 39.2 (1C, C7), 38.4 (1C, d, *J* = 10.9 Hz, C1), 37.5 (1C, C11), 35.9 (1C, C10), 30.4 (1C, C4), 27.9 (1C, C5), 13.7 (3C, d, *J* = 28.1 Hz, PMe_3).

Compound 9

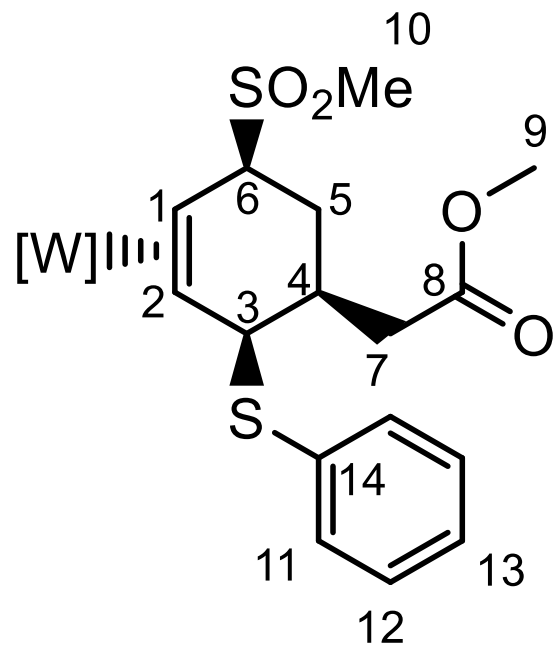

Compound **6** (100 mg; 0.113 mmol) was taken in DCM (1.5 mL) and cooled to -40°C. KSPh (25 mg; 0.170 mmol) was taken separately in THF (3 mL) and cooled to -40°C. The solution of **6** was then added to the thiol salt and stirred cold for 1.5 hrs. The crude reaction mixture was then precipitated into stirring pentane (50 mL) and filtered through a fine porosity fritted disc. The precipitate was washed with DCM (2 mL) and the combined filtrate was evaporated to dryness in a tared vial to yield Compound **9** as a yellow oil (78 mg; 0.092 mmol; 82%).

^{1}H NMR (800 MHz, Acetone) δ 8.48 (1H, d, *J* = 2.0 Hz, Tp3A), 8.14 (1H, d, *J* = 1.9 Hz, Tp3B), 7.96 (1H, dt, *J* = 2.4, 0.7 Hz, Tp5B), 7.94 (1H, d, *J* = 2.3 Hz, Tp5C), 7.86 (1H, dt, *J* = 2.4, 0.7 Hz, Tp5A), 7.45 (1H, d, *J* = 2.2 Hz, Tp3C), 7.02 (5H, m, H11-13), 6.40 (1H, t, *J* = 2.2 Hz, Tp4B), 6.34 (1H, t, *J* = 2.2 Hz, Tp4A), 6.30 (1H, t, *J* = 2.2 Hz, Tp4C), 4.80 (1H, dd, *J* = 3.4, 1.7 Hz, H3), 4.45 (1H, dd, *J* = 10.7, 8.3 Hz, H6), 3.43 (3H, s, H9), 3.16 (1H, m, H4), 3.13 (3H, s, H10), 3.11 (1H, m, H1), 2.76 (1H, dd, *J* = 17.1, 10.5 Hz, H7), 2.40 (1H, dd, *J* = 17.1, 3.9 Hz, H7), 1.88 (1H, td, *J* = 13.0, 10.8 Hz, H5), 1.83 (1H, dddd, *J* = 13.2, 8.2, 3.6, 1.4 Hz, H5), 1.59 (1H, m, H2), 1.29 (9H, d, *J* = 8.8 Hz, PMe_3). **^{13}C NMR (201 MHz, Acetone) δ** 173.5 (1C, C8), 145.4 (1C, Tp3A), 145.1 (1C, Tp3B), 142.1 (1C, Tp3C), 139.5 (1C, C14), 137.6 (1C, Tp5C), 137.5 (Tp5B), 137.4 (1C, Tp5A), 130.6 (2C, C11/C12), 129.4 (2C, C11/12), 126.1 (1C, C13), 107.2 (1C, Tp4B), 107.0 (1C, Tp4C), 106.6 (1C, Tp4A), 64.0 (1C, C6), 61.0 (1C, C2), 56.2 (1C, C3), 51.5 (1C, C9), 39.4 (1C, C7), 39.2 (1C, d, *J* = 11.1 Hz, C1), 35.9 (1C, C10), 31.3 (1C, C4), 28.7 (1C, C5), 13.6 (3C, d, *J* = 28.2 Hz, PMe_3).

Compound 10

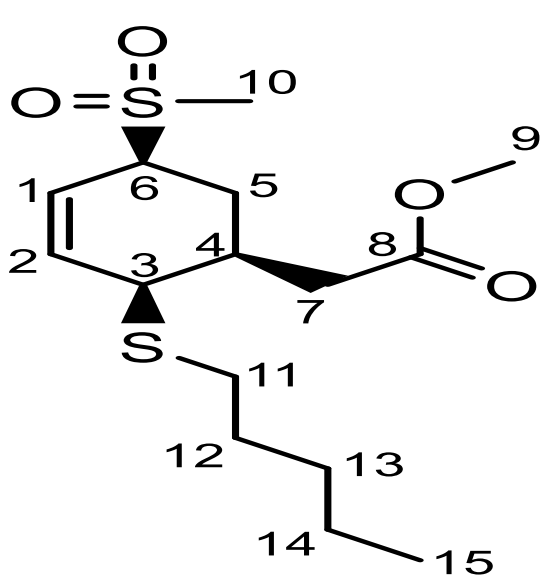

Compound **6** (305 mg; 0.345 mmol) was taken in DCM (3 mL) and cooled to -30°C. KSAc (59 mg; 0.518 mmol) was taken separately in THF (3 mL) and cooled to -30°C. The solution of **6** was then added to the thioacetate salt and stirred cold for 1 hr. The crude reaction mixture was then precipitated into stirring hexane (100 mL) and filtered through a fine porosity fritted disc. The precipitate was washed with DCM (2 mL), the combined filtrate was evaporated to half volume and filtered through another fine porosity fritted disc to yield Compound **10** as a white powder (200 mg; 0.247 mmol; 72%).

^{1}H NMR (800 MHz, CD_3CN) δ 8.33 (1H, d, *J* = 2.1 Hz, Tp3A), 8.06 (1H, d, *J* = 2.0 Hz, Tp3B), 7.87 (1H, d, *J* = 2.6 Hz, Tp5B), 7.86 (1H, d, *J* = 2.3 Hz, Tp5C), 7.76 (1H, d, *J* = 2.4 Hz, Tp5A), 7.31 (1H, d, *J* = 2.2 Hz, Tp3C), 6.38 (1H, t, *J* = 2.2 Hz, Tp4B), 6.31 (1H, t, *J* = 2.2 Hz, Tp4A), 6.29 (1H, t, *J* = 2.2 Hz, Tp4C), 4.87 (1H, s, H3), 4.44 (1H, t, *J* = 9.3 Hz, H6), 3.65 (3H, s, H9), 3.11 (1H, tq, *J* = 10.1, 3.7 Hz, H4), 2.98 (1H, ddd, *J* = 13.8, 11.0, 1.8 Hz, H1), 2.95 (3H, s, H10), 2.43 (1H, dd, *J* = 16.7, 9.9 Hz, H7), 2.34 (1H, m, H7), 2.18 (3H, s, H12), 1.85 (1H, dddd, *J* = 13.6, 8.5, 3.8, 1.6 Hz, H5), 1.53 (1H, td, *J* = 13.2, 10.3 Hz, H5), 1.20 (9H, d, *J* = 8.8 Hz, PMe_3), 1.17 (1H, m, H2).

^{13}C NMR (201 MHz, CD_3CN) δ 196.7 (1C, C11), 173.6 (1C, C8), 145.5 (1C, Tp3A), 145.0 (1C, Tp3B), 142.1 (1C, Tp3C), 138.1 (1C,Tp5C), 137.8 (1C, Tp5B), 137.6 (1C, Tp5A), 107.6 (1C, Tp4B), 107.3 (1C, Tp4C), 107.0 (1C, Tp4A), 63.7 (1C, C6), 60.9 (1C, C2), 52.0 (1C, C9), 51.9 (1C, C3), 39.5 (1C, C7), 38.4 (1C, d, *J* = 11.0 Hz, C1), 37.2 (1C, C10), 31.2 (1C, C12), 30.4 (1C, C4), 29.4 (1C, C5), 13.4 (3C, d, *J* = 28.4 Hz, PMe_3).

Compound 11

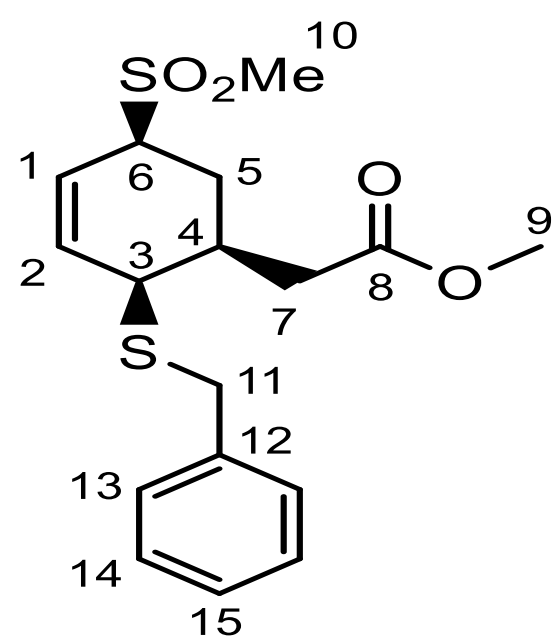

Compound **6** (100 mg; 0.113 mmol) was taken in DCM (1.5 mL) and cooled to -30°C. $KSCH_2CO_2CH_3$ (25 mg; 0.173 mmol) was taken separately in THF (2 mL) and cooled to -30°C. The solution of **6** was then added to the thioacetate salt and stirred cold for 4 hr. The crude reaction mixture was then precipitated into stirring pentane (70 mL) and filtered through a fine porosity fritted disc. The precipitate was washed with DCM (2 mL) and the combined filtrate was evaporated to dryness in a tared vial to yield Compound **11** as a clear oil (80 mg; 0.0.095 mmol; 84%).

(not a 2D)

^{1}H NMR (800 MHz, $CDCl_3$) δ 8.17 (d, *J* = 2.1 Hz, 2H), 8.10 (d, *J* = 2.0 Hz, 1H), 7.75 (d, *J* = 2.3 Hz, 1H), 7.73 (dd, *J* = 2.4, 0.7 Hz, 1H), 7.63 (d, *J* = 2.5 Hz, 1H), 7.26 (d, *J* = 2.2 Hz, 1H), 6.30 (t, *J* = 2.2 Hz, 1H), 6.27 (t, *J* = 2.2 Hz, 1H), 6.26 (t, *J* = 2.2 Hz, 1H), 4.51 (t, *J* = 9.4 Hz, 1H), 4.18 (t, *J* = 2.4 Hz, 1H), 3.73 (s, 3H), 3.29 (s, 2H), 3.20 (d, *J* = 14.6 Hz, 1H), 3.13 (m, 2H), 3.08 (s, 3H), 2.97 (ddd, *J* = 13.7, 10.9, 1.6 Hz, 1H), 2.83 (dd, *J* = 16.5, 10.1 Hz, 1H), 2.48 (dd, *J* = 16.5, 4.2 Hz, 1H), 1.94 (dddd, *J* = 13.6, 8.5, 3.7, 1.4 Hz, 1H), 1.72 (td, *J* = 13.3, 10.3 Hz, 1H), 1.62 (m, 1H), 1.26 (d, *J* = 8.5 Hz, 9H). **^{13}C NMR (201 MHz, $CDCl_3$) δ** 173.0, 171.3, 144.7, 144.1, 140.8, 136.6, 136.4, 136.1, 106.6, 106.2, 63.4, 60.9, 53.8, 51.9, 51.7, 39.1, 38.5 (d, *J* = 10.9 Hz), 35.9, 34.7, 30.6, 27.6, 13.6 (d, *J* = 28.1 Hz).

Compound 13

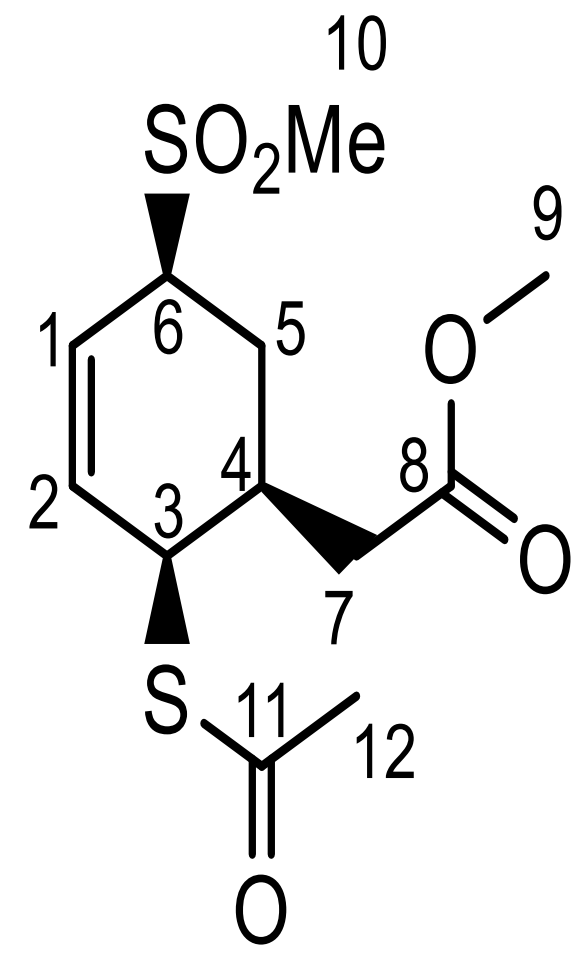

Compound **5** (50 mg; 0.068 mmol) was taken in 2 mL MeCN, cooled to -30°C, and 1M HOTf in MeCN (102 µL; 0.102 mmol) was added slowly. In a separate tube, KSBn was taken in THF (2 mL) and just enough MeOH to dissolve (~0.25 mL) and cooled to -30°C. After 15 min, the KSBn solution was added to the allyl and stirred at -30°C for 18 hrs and at 25°C for 3 hrs. The crude reaction mixture was quenched with Na_2CO_3(aq) and extracted with DCM. The organic layer was dried over Na_2SO_4 and evaporated to dryness in a tared vial to yield compound **13** as a clear oil with no further purification (55 mg; 0.068 mmol; 89%).

^{1}H NMR (800 MHz, Acetone) δ 8.18 (1H, d, *J* = 2.0 Hz, Tp3A), 8.02 (1H, d, *J* = 2.0 Hz, Tp3B), 7.92 (1H, d, *J* = 2.3 Hz, Tp5C), 7.90 (1H, d, *J* = 2.4 Hz, Tp5B), 7.83 (1H, d, *J* = 2.5 Hz, Tp5A), 7.45 (2H, m, H17), 7.31 (2H, m, H18), 7.23 (1H, td, *J* = 7.2, 1.4 Hz, H19), 7.09 (1H, d, *J* = 2.2 Hz, Tp3C), 7.01 (3H, m, H13/H14), 6.93 (2H, m, H12), 6.35 (1H, t, *J* = 2.2 Hz, Tp4B), 6.28 (1H, t, *J* = 2.2 Hz, Tp4A), 6.27 (1H, t, *J* = 2.2 Hz, Tp4C), 4.13 (2H, m, H3/H6), 3.98 (1H, d, *J* = 13.4 Hz, H15), 3.87 (1H, d, *J* = 13.3 Hz, H15), 3.63 (3H, s, H9), 3.59 (3H, s, H10), 2.96 (1H, m, H4), 2.84 (1H, m, H7), 2.59 (1H, ddd, *J* = 14.7, 11.1, 2.1 Hz, H1), 2.40 (1H, dd, *J* = 16.3, 4.2 Hz, H7), 1.84 (2H, m, H5), 1.34 (1H, m, H2), 1.12 (9H, d, *J* = 8.3 Hz, PMe_3). **^{13}C NMR (201 MHz, Acetone) δ** 173.9 (1C, C8), 145.0 (1C, Tp3A), 144.2 (1C, Tp3B), 140.9 (1C, C11) 141.0 (1C, C16), 141.4 (1C, Tp3C), 137.3 (1C, Tp5C), 137.0 (1C, Tp5B), 136.9 (1C, Tp5A), 130.1 (2C, C17), 129.4 (2C, C18), 129.2 (2C, C13), 128.8 (2C, C12), 127.3 (1C, C19), 127.0 (1C, C14), 107.1 (1C, Tp4B), 107.0 (1C, Tp4A), 106.7 (1C, Tp4C), 61.8 (1C, J_W=14.3, C2), 54.1 (1C, C3), 51.5 (1C, C9), 49.7 (1C, d, J=10.1, C1), 43.6 (1C, d, J=2.8, C6), 39.5 (1C, C7), 37.9 (1C, C10), 36.8 (1C, C5), 32.8 (1C, C4), 34.8 (1C, C15), 14.5 (3C, d, J=27.3, PMe3). **HRMS (APCI$^+$):** [M-SBn]$^+$ calcd. for $C_{28}H_{38}BN_7O_3PSW$, 778.2097; found, 778.2100.

Compound 14

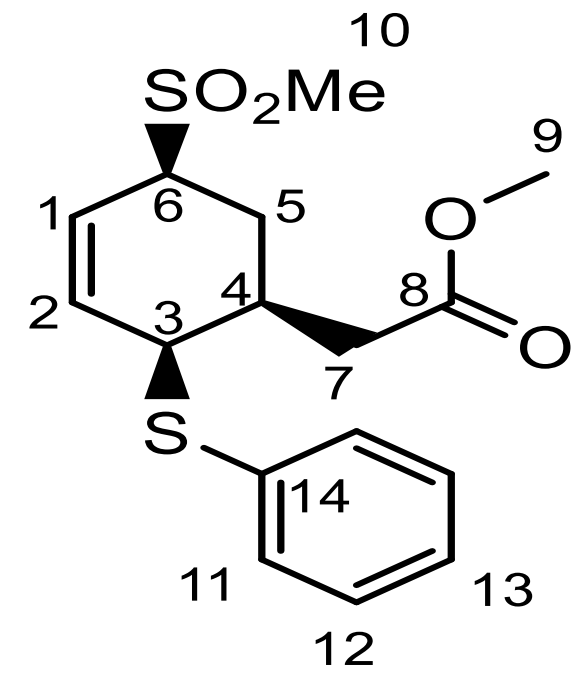

Compound **12** (50 mg; 0.066 mmol) and $KS(C_5H_{11})$ were taken in THF (4 mL) and MeOH (0.5 mL) and the reaction was stirred at 25°C for 5 hrs. The crude reaction was then precipitated into 30 mL of stirring Et_2O and filtered through a medium porosity fritted disc. The precipitate was washed with DCM (2x2 mL) and the combined filtrate was evaporated to dryness. The crude product was dissolved in DCM (2 mL), precipitated into 30 mL stirring pentane, and compound **14** was collected as a white powder on a fine porosity fritted disc (11 mg; 0.014 mmol; 21%)

^{1}H NMR (600 MHz, CD_3CN) δ 8.02 (1H, d, *J* = 2.1 Hz, Tp3B), 7.95 (1H, d, *J* = 2.2 Hz, Tp3A), 7.86 (2H, m, Tp5B/Tp5C), 7.80 (1H, dt, *J* = 2.5, 0.8 Hz, Tp5A), 7.31 (1H, d, *J* = 2.2 Hz, Tp3C), 6.37 (1H, t, *J* = 2.2 Hz, Tp4B), 6.31 (1H, t, *J* = 2.2 Hz, Tp4A), 6.28 (1H, t, *J* = 2.2 Hz, Tp4C), 4.11 (1H, t, *J* = 8.6 Hz, H6), 3.81 (1H, d, *J* = 4.3 Hz, H3), 3.70 (3H, s, H9), 2.71 (2H, m, H11), 2.61 (2H, m, H1/H4), 2.55 (1H, dd, *J* = 16.6, 4.8 Hz, H7), 2.48 (1H, dd, *J* = 16.8, 10.2 Hz, H7), 1.90 (1H, d, *J* = 6.9 Hz, H5), 1.67 (2H, m, H12), 1.60 (1H, td, *J* = 13.1, 11.0 Hz, H5), 1.44 (2H, dddd, *J* = 14.2, 8.2, 7.2, 5.9 Hz, H13), 1.37 (2H, m, H14), 1.29 (9H, d, *J* = 8.7 Hz, PMe_3), 1.04 (1H, d, *J* = 11.1 Hz, H2), 0.93 (3H, t, *J* = 7.3 Hz, H15). **^{13}C NMR (201 MHz, CD_3CN) δ** 173.6 (1C, C9), 144.7 (1C, Tp3B), 144.0 (1C, Tp3A), 141.8 (1C, Tp3C), 137.9 (1C, Tp5A/B/C), 137.8 (1C, Tp5A/B/C), 137.7 (1C, Tp5A/B/C), 126.5 (1C, C10), 107.6 (1C, Tp4B), 107.4 (1C, Tp4C), 107.3 (1C, Tp4A), 55.9 (1C, C2), 52.3 (1C, C9), 49.0 (1C, d, *J* = 11.1 Hz, C1), 43.6 (1C, d, *J* = 2.7 Hz, C6), 39.5 (1C, C7), 37.7 (1C, C5), 35.8 (1C, C3), 32.1 (1C, C13), 30.8 (1C, C4), 30.7 (1C, C11), 30.3 (1C, C12), 23.1 (1C, C14), 14.6 (3C, d, *J* = 28.5 Hz, PMe_3), 14.3 (1C, C15).

Compound 15

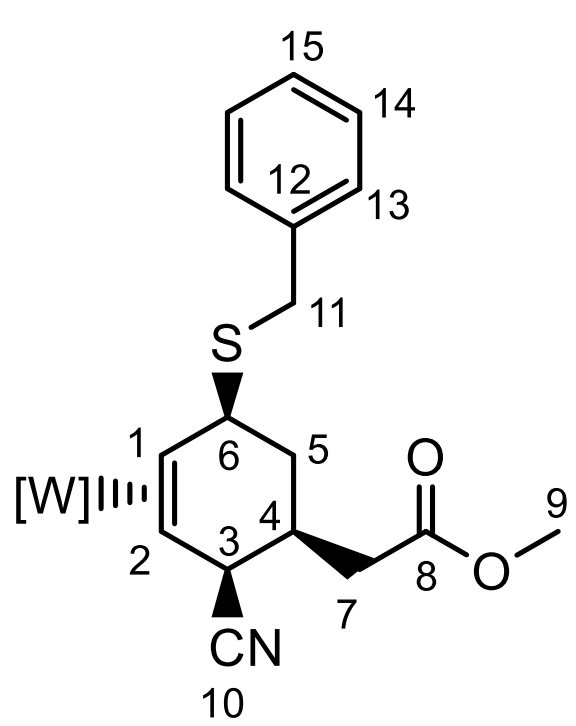

Compound **12** (150 mg; 0.197 mmol) was taken in 3 mL MeOH. To this a, solution of BnSH (110 mg; 0.888 mmol) and NaOtBu (56.9 mg; 0.592 mmol) in THF (3 mL) was added and stirred at 25°C for 7 hrs. 4 mL was H_2O was then added to induce precipitation, the precipitate was collected on a fine porosity fritted disc to yield compound **15** as a white powder (110 mg; 0.137 mmol; 69%).

^{1}**H NMR (800 MHz, Acetone) δ** 8.14 (1H, d, *J* = 2.0 Hz, Tp3A), 8.05 (1H, d, *J* = 2.0 Hz, Tp3B), 7.93 (2H, m, Tp5B/Tp5C), 7.84 (1H, dt, *J* = 2.4, 0.7 Hz, Tp5A), 7.47 (2H, m, H13), 7.33 (2H, dd, *J* = 8.4, 6.9 Hz, H14), 7.25 (2H, m, Tp3C/H15), 6.38 (1H, t, *J* = 2.2 Hz, Tp4B), 6.32 (2H, td, *J* = 2.3, 1.5 Hz, Tp4A/Tp4C), 4.12 (1H, m, H6), 3.98 (2H, m, H11/H3), 3.92 (1H, d, *J* = 13.3 Hz, H11), 3.72 (3H, s, H9), 2.75 (1H, dddd, *J* = 17.1, 8.5, 4.3, 2.8 Hz, H4), 2.62 (1H, dd, *J* = 16.9, 4.5 Hz, H7), 2.56 (2H, m, H1/H7), 2.06 (1H, m, H5), 1.82 (1H, m, H5), 1.17 (9H, d, *J* = 8.6 Hz, PMe_3), 1.09 (1H, m, H2). ^{13}**C NMR (201 MHz, Acetone) δ** 173.3 (1C, C8), 144.5 (1C, Tp3A), 144.2 (1C, Tp3B), 141.7 (1C, Tp3C), 140.6 (1C, C12) 137.7 (1C, Tp5C), 137.4 (1C, Tp5A/Tp5B), 130.1 (2C, C13), 129.3 (2C, C14), 127.5 (1C, C15), 126.2 (1C, C10), 107.3/107.0 (2C, Tp4A/Tp4C), 107.2 (1C, Tp4B), 55.6 (1C, C2), 51.8 (1C, C9), 48.6 (1C, d, J = 11.5, C1), 43.0 (1C, d, J = 2.7, C6), 39.2 (1C, C7), 37.2 (1C, C5), 35.7 (1C, C3), 35.0 (1C, C11), 30.6 (1C, C4), 14.5 (3C, d, J = 28.3, PMe3).

Compound 16

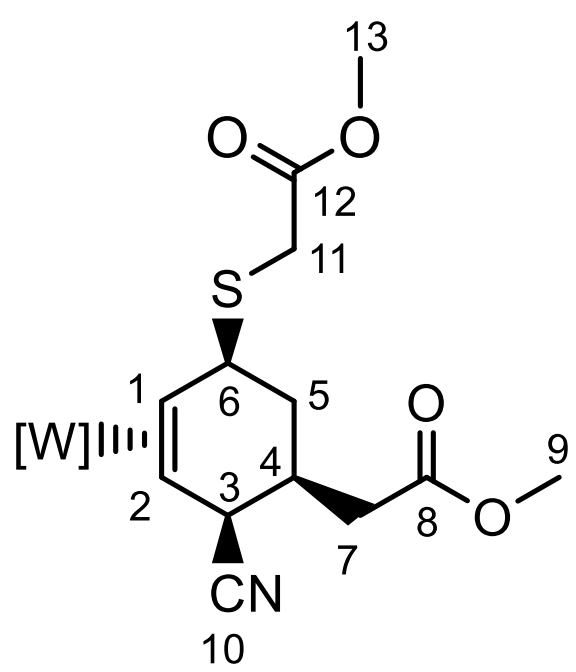

Compound **12** (112 mg; 0.147 mmol) and $KSCH_2CO_2CH_3$ were taken in THF (5 mL) and MeOH (0.5 mL) and the reaction was stirred at 25°C for 4 hrs. The crude reaction was then precipitated into 50 mL of stirring Et_2O and filtered through a medium porosity fritted disc. The precipitate was washed with DCM (2x3 mL) and the combined filtrate was evaporated to dryness. The crude product was dissolved in DCM (2 mL), precipitated into 40 mL stirring pentane, and compound **16** was collected as a white powder on a fine porosity fritted disc (95 mg; 0.121 mmol; 82%)

^{1}**H NMR (800 MHz, $CDCl_3$) δ** 8.12 (1H, d, *J* = 2.0 Hz, Tp3A), 8.04 (1H, d, *J* = 2.0 Hz, Tp3B), 7.71 (1H, d, *J* = 2.3 Hz, Tp5C), 7.70 (1H, dt, *J* = 2.4, 0.7 Hz, Tp5A), 7.63 (1H, d, *J* = 2.5 Hz, Tp5B), 7.21 (1H, d, *J* = 2.1 Hz, Tp3C), 6.29 (1H, t, *J* = 2.1 Hz, Tp4B), 6.27 (1H, t, *J* = 2.2 Hz, Tp4A), 6.24 (1H, t, *J* = 2.2 Hz, Tp4C), 4.25 (1H, m, H6), 4.05 (1H, dd, *J* = 3.9, 1.9 Hz, H3), 3.75 (3H, s, H13), 3.71 (3H, s, H9), 3.51 (1H, d, *J* = 14.8 Hz, H11), 3.46 (1H, d, *J* = 14.8 Hz, H11), 2.69 (1H, dddd, *J* = 14.5, 6.9, 5.3, 2.6 Hz, H4), 2.60 (2H, m, H1/H7), 2.55 (1H, dd, *J* = 17.1, 4.2 Hz, H7), 1.93 (1H, dddd, *J* = 13.2, 6.6, 2.7, 1.2 Hz, H5), 1.75 (1H, td, *J* = 12.7, 10.4 Hz, H5), 1.31 (9H, d, *J* = 8.5 Hz, PMe_3), 1.28 (1H, m, H2). ^{13}**C NMR (201 MHz, $CDCl_3$) δ** 172.6 (1C, C8), 171.7 (1C, C12), 143.9 (1C, Tp3A), 143.6 (1C, Tp3B), 140.4 (1C, Tp3C), 136.6 (1C, Tp5C), 136.4 (1C, Tp5A), 136.3 (1C, Tp5B), 126.0 (1C, C10), 106.6 (1C, Tp4B), 106.5 (1C, Tp4A), 106.4 (1C, Tp4C), 55.0 (1C, C2), 52.5 (1C, C13), 51.7 (1C, C9), 47.9 (1C, d, *J* = 11.1 Hz, C1), 43.4 (1C, d, J= 2.6 Hz, C6), 38.5 (1C, C7), 35.9 (1C, C5), 35.0 (1C, C3), 31.9 (1C, C11), 29.8 (1C, C4), 14.7 (3C, d, *J* = 28.1 Hz, PMe_3).

Compound 17

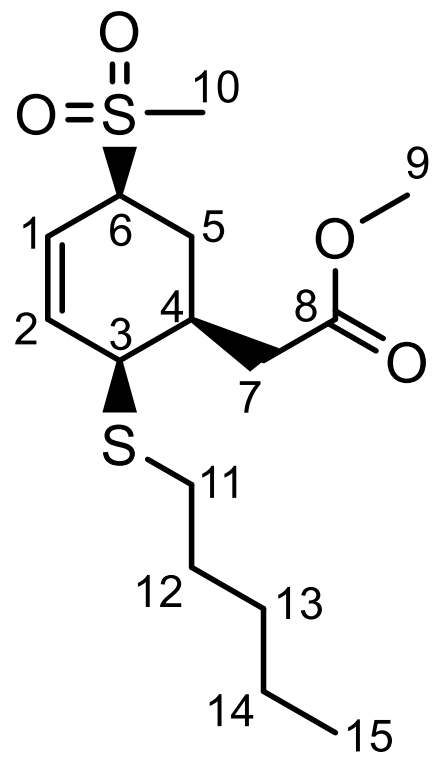

Compound **5** (148 mg; 0.168 mmol) was taken in DCM (2 mL) and cooled to -30°C. $KS(C_5H_{11})$ was taken in THF (1.5 mL) and cooled to -30°C, before being added to **5** and stirred cold 18 hrs. The crude reaction mixture was then precipitated directly into stirring pentane (75 mL), and filtered through a medium porosity fritted disc. The precipitate was washed with DCM (2x3 mL) and the combined filtrate was evaporated to dryness to yield compound **6** as a crude solid. This was then redissolved in DCM (2 mL) and cooled to -30°C. Solid CAN (101 mg; 0.184 mmol) was added to this followed by cold (-30°C) ACN (3mL) and the mixture was stirred 6 hr. The reaction was quenched with 20 mL 5% Na_2SO_3(aq) and extracted with DCM (20 mL). The organic layer was dried over Na_2SO_4, evaporated to dryness, and further purified by flash chromatography (CombiFlash, 4g silica, Hex -> EtOAc). Compound **17** was collected from the 30% EtOAc/Hex portion into a weighed vial, and evaporated to a clear oil (13 mg; 0.039 mmol; 23%).

^{1}H NMR (600 MHz, CD3CN) δ 6.28 (1H, ddd, *J* = 10.0, 5.9, 2.6 Hz, H2), 5.77 (1H, ddt, *J* = 9.9, 2.4, 1.3 Hz, H1), 3.80 (1H, ddt, *J* = 8.8, 6.4, 2.4 Hz, H6), 3.64 (3H, s, H9), 3.45 (1H, ddt, *J* = 5.9, 4.1, 1.8 Hz, H3), 2.76 (3H, s, H10), 2.70 (1H, dd, *J* = 16.4, 8.0 Hz, H7), 2.56 (2H, m, H11), 2.48 (1H, dd, *J* = 16.4, 6.4 Hz, H7), 2.41 (1H, m, H4), 1.98 (1H, m, H5), 1.70 (1H, td, *J* = 12.8, 11.3 Hz, H5), 1.54 (2H, m, H12), 1.32 (4H, m, H13/14), 0.89 (3H, t, *J* = 7.1 Hz, H15). **^{13}C NMR (151 MHz, CD_3CN) δ** 173.3 (1C, C8), 136.3 (1C, C2), 119.0 (1C, C1), 62.3 (1C, C6), 52.2 (1C, C9), 44.9 (1C, C3), 38.4 (1C, C7), 37.4 (1C, C10), 34.9 (1C, C4), 33.8 (1C, C11), 31.6 (1C, C13/14), 30.6 (1C, C12), 25.5 (1C, C5), 22.9 (1C, C13/14), 14.3 (1C, C15). **HRMS (APCI$^+$, deconvoluted):** [M] calcd. for $C_{17}H_{22}O_4S_2$, 334.1273; found, 334.1269.

Compound 18

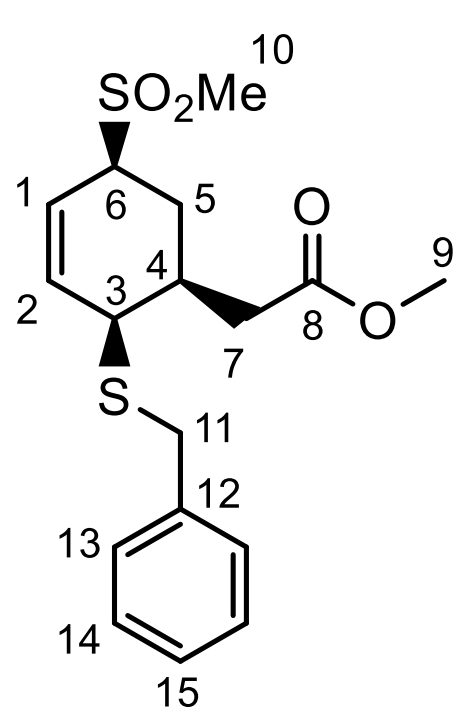

Compound **8** (84 mg; 0.098 mmol) was taken in MeCN (5 mL). To this, a solution of DDQ (48.9 mg; 0.215 mmol) in ACN (2 mL) was added. After 5 min the reaction was then quenched with saturated

NaHCO3(aq) (10 mL) and extracted with DCM (15 mL). The organic layer was dried over Na_2SO_4, evaporated to dryness, redissolved in minimal DCM (2 mL) and precipitated into 30 mL stirring hexane. The resulting solution was filtered through a fine porosity fritted disc, the remaining solids washed with Et2O (2x2 mL) and the combined filtrates evaporated in vacuo. The product was further purified by preparatory thin-layer chromatography (20x20 cm; 80% Hex: 20% EtOAc). A uv band (Rf ~0.33) was collected, the silica extracted with EtOAc into a weighed vial and evaporated to yield Compound **18** as a clear oil (8 mg; 0.023 mmol: 23%).

^{1}H NMR (800 MHz, $CDCl_3$) δ 7.32 (m, 5H), 7.26 (dt, *J* = 13.9, 1.7 Hz, 1H), 6.12 (ddd, *J* = 10.0, 6.0, 2.5 Hz, 1H), 5.81 (ddt, *J* = 9.9, 2.4, 1.2 Hz, 1H), 3.77 (m, 2H), 3.70 (s, 5H), 3.35 (m, 1H), 2.73 (d, *J* = 0.8 Hz, 3H), 2.68 (dd, *J* = 16.2, 7.5 Hz, 1H), 2.49 (dd, *J* = 16.2, 6.5 Hz, 1H), 2.45 (dddd, *J* = 14.3, 6.8, 3.9, 1.9 Hz, 1H), 2.12 (m, 1H), 1.77 (td, *J* = 12.9, 11.3 Hz, 1H), 1.56 (d, *J* = 12.9 Hz, 1H). **^{13}C NMR (201 MHz, $CDCl_3$) δ** 172.4, 138.0, 135.7, 129.0, 128.8, 127.6, 118.9, 62.1, 52.0, 43.3, 37.8, 37.6, 35.7, 34.2, 25.7. **HRMS (APCI$^+$, deconvoluted):** [M] calcd. for $C_{17}H_{22}O_4S_2$, 354.0960; found, 354.0956.

Compound 20

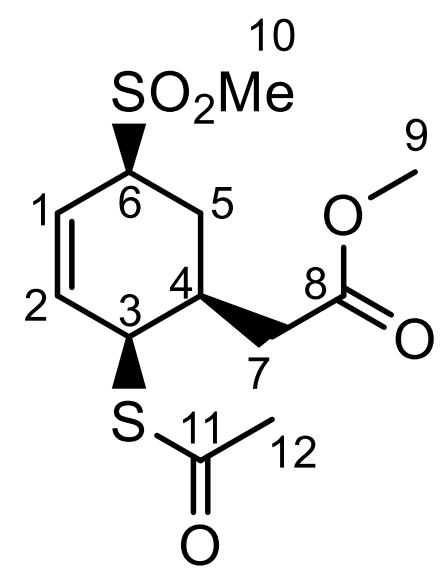

Compound **10** (100 mg; 0.124 mmol) was taken in MeCN (5 mL) and cooled to -30°C. To this, a solution of DDQ (61.7 mg; 0.272 mmol) in MeCN (2 mL) at -30°C was added. The combined reaction was then brought to 25°C. After 5 min, the reaction was quenched with saturated NaHCO3(aq) (15 mL) and extracted with 20 mL DCM. The organic layer was dried over Na_2SO_4, evaporated to dryness, redissolved in minimal DCM (2 mL) and precipitated into 40 mL stirring hexane. The resulting solution was filtered through a fine porosity fritted disc, the remaining solids washed with Et2O (2x4 mL) and the combined filtrates evaporated in vacuo. The product was further purified by preparatory thin-layer chromatography (20x20 cm; 40% Hex: 60% EtOAc). A uv band (Rf ~0.25) was collected, the silica extracted with EtOAc into a weighed vial and evaporated to yield Compound **20** as a clear oil (11 mg; 0.036 mmol: 29%).

^{1}H NMR (800 MHz, CD_3CN) δ 6.16 (1H, ddd, *J* = 9.9, 5.9, 2.5 Hz, H2), 5.90 (1H, ddt, *J* = 9.9, 2.5, 1.2 Hz, H1), 4.29 (1H, m, H3), 3.89 (1H, ddt, *J* = 11.1, 6.6, 2.3 Hz, H6), 3.62 (3H, s, H10), 2.79 (3H, s, H7), 2.55 (1H, m, H4), 2.44 (1H, dd, *J* = 16.7, 7.1 Hz, H8), 2.34 (1H, m, H8), 2.32 (3H, d, *J* = 6.5 Hz, H12), 2.10 (1H, dddd, *J* = 12.8, 6.3, 2.8, 1.5 Hz, H5), 1.53 (1H, td, *J* = 13.0, 11.2 Hz, H5). **^{13}C NMR (201 MHz, CD_3CN) δ** 195.2 (1C, C11), 172.8 (1C, C9), 134.2 (1C, C2), 121.6 (1C, C1), 61.9 (1C, C6), 52.1 (1C, C10), 43.0 (1C, C3), 37.7 (1C, C7), 37.6 (1C, C8), 33.8 (1C, C4), 30.6 (1C, C12), 26.2 (1C, C5). **HRMS (APCI$^+$, deconvoluted):** [M] calcd. for $C_{12}H_{18}O_5S_2$, 306.0596; found, 306.0592.

Compound 19

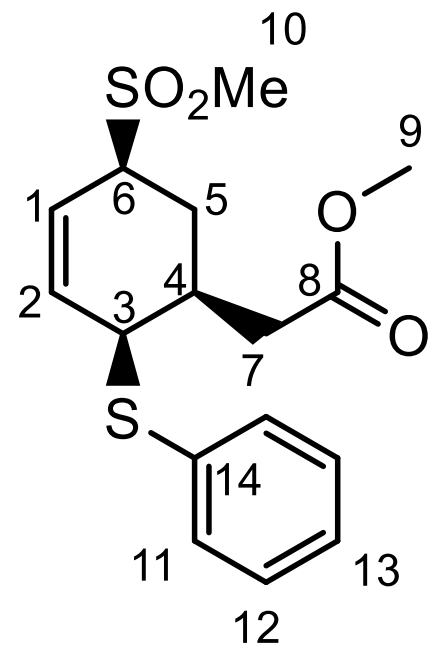

Compound **9** (110 mg; 0.130 mmol) was taken in MeCN (10 mL) and cooled to -30°C. To this a solution of DDQ (56.0 mg; 0.248 mmol) in 2 mL of ACN at -30°C was added, and the combined reaction warmed to 25°C over 5 min. The reaction was then quenched with 10 mL saturated $NaHCO_3$(aq) and extracted with 15 mL DCM. The organic layer was dried over Na_2SO_4, evaporated to dryness, redissolved in minimal DCM (2 mL) and precipitated into 40 mL stirring hexane. The resulting solution was filtered through a fine porosity fritted disc, the remaining solids washed with Et_2O (2x2 mL) and the combined filtrates evaporated in vacuo. A column was prepared by filling a 30 mL medium porosity fritted disc with 20 mL with silica. The product was then purified by flash chromatography (Hex -> EtOAc) and compound **19** was collected from the 40- 50% EtOAc/Hex into a weighed vial and evaporated to a clear oil (10 mg; 0.030 mmol: 34%).

^{1}H NMR (800 MHz, $CDCl_3$) δ 7.44 (2H, dt, *J* = 8.0, 1.3 Hz, H12), 7.29 (2H, ddt, *J* = 7.8, 6.2, 1.3 Hz, H13), 7.25 (1H, m, H14), 6.36 (1H, ddt, *J* = 9.8, 5.8, 1.9 Hz, H2), 5.94 (1H, ddd, *J* = 9.9, 2.6, 1.4 Hz, H1), 4.01 (1H, ddt, *J* = 6.0, 4.1, 1.7 Hz, H3), 3.71 (1H, m, H6), 3.50 (3H, d, *J* = 1.4 Hz, H10), 2.69 (4H, m, H7/H8), 2.56 (1H, dddq, *J* = 12.6, 6.2, 4.2, 2.5 Hz, H4), 2.43 (1H, ddd, *J* = 16.8, 6.2, 1.3 Hz, H8), 2.10 (1H, m, H5), 1.83 (1H, tdd, *J* = 13.0, 11.4, 1.4 Hz, H5). **^{13}C NMR (201 MHz, $CDCl_3$) δ** 171.9 (1C C9), 134.8 (1C, C2), 134.6 (1C, C11), 132.7 (1C, C12), 129.2 (1C, C13), 127.6 (1C, C14), 120.2 (1C, C1), 62.0 (1C, C6), 51.6 (1C, C10), 48.2 (1C, C3), 37.6 (1C, C8), 35.4 (1C, C7), 34.4 (1C, C4), 25.06 (1C, C5). **HRMS (APCI$^+$, deconvoluted):** [M] calcd. for $C_{16}H_{20}O_4S_2$, 340.0803; found, 340.0802.

Compound 21

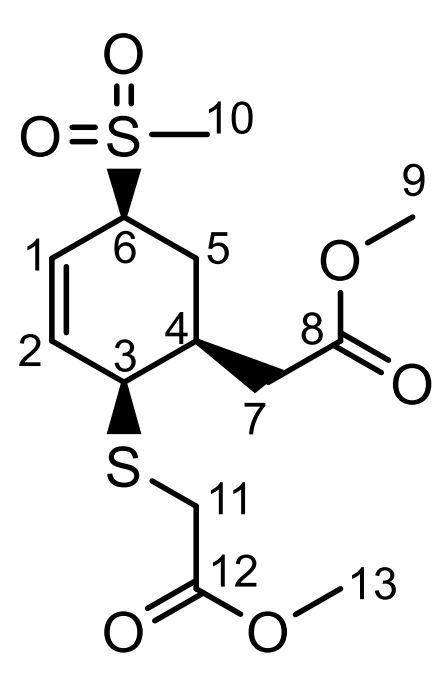

Compound **11** (73 mg; 0.087 mmol) was taken in MeCN (5 mL). To this, a solution of DDQ (43.4 mg; 0.191 mmol) in MeCN (2 mL) was added. After five minutes the reaction was then quenched saturated NaHCO3(aq) (10 mL) and extracted with DCM (15 mL). The organic layer was dried over Na_2SO_4, evaporated to dryness, redissolved in minimal DCM (2 mL) and precipitated into 30 mL stirring hexane. The resulting solution was filtered through a fine porosity fritted disc, the remaining solids washed with

Et2O (2x2 mL) and the combined filtrates evaporated in vacuo. A column was prepared by filling a 30 mL medium porosity fritted disc with 20 mL silica. The product was then purified by flash chromatography (Hex -> EtOAc) and the 50% EtOAc/Hex portion was evaporated into a weighed vial to yield Compound **21** as a clear oil (10 mg; 0.030 mmol: 34%).

^{1}H NMR (800 MHz, $CDCl_3$) δ 6.34 (ddd, *J* = 9.9, 5.9, 2.5 Hz, 1H), 5.92 (ddd, *J* = 9.9, 2.5, 1.2 Hz, 1H), 3.74 (s, 3H), 3.72 (m, 0H), 3.70 (s, 3H), 3.68 (m, 1H), 3.35 (d, *J* = 15.1 Hz, 1H), 3.29 (d, *J* = 15.1 Hz, 1H), 2.76 (d, *J* = 0.8 Hz, 3H), 2.73 (m, 1H), 2.50 (m, 2H), 2.12 (dddd, *J* = 11.9, 6.7, 2.6, 1.3 Hz, 1H), 1.76 (td, *J* = 13.0, 11.3 Hz, 1H). **^{13}C NMR (201 MHz, $CDCl_3$) δ** 172.3, 170.7, 134.9, 120.0, 62.0, 52.8, 52.0, 44.5, 37.7, 36.0, 34.2, 34.1, 25.2. **HRMS (APCI$^+$, deconvoluted):** [M] calcd. for $C_{13}H_{20}O_6S_2$, 336.0701; found, 336.0700.

Compound 22

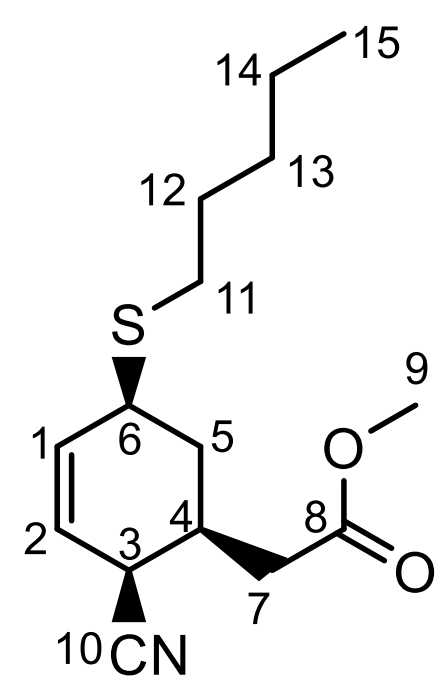

Compound **12** (50 mg; 0.66 mmol) and $KS(C_5H_{11})$ was taken in THF (4 mL) and MeOH (0.5 mL) and the reaction was stirred at 25°C for 5 hrs. The crude reaction was then precipitated into stirring Et_2O (30 mL) and filtered through a medium porosity fritted disc. The precipitate was washed with DCM (2x2 mL) and the combined filtrate was evaporated to dryness to yield compound **13** as a crude solid. This was then redissolved in DCM (2 mL) and cooled to -30°C. Solid CAN (40 mg; 0.072 mmol) was added to this followed by cold (-30°C) ACN (3mL) and the mixture was stirred 1 hr. The reaction was quenched with 7 mL 10% Na_2SO_3(aq) and extracted with DCM (15 mL). The organic layer was dried over Na_2SO_4, evaporated to dryness, and further purified by flash chromatography (CombiFlash, 4g silica, Hex -> 10% EtOAc/Hex -> 25% EtOAc/Hex). Compound **21** was collected from the 10% EtOAc/Hex portion into a weighed vial, and evaporated to a clear oil (7 mg; 0.025 mmol; 38%).

^{1}H NMR (600 MHz, CD_3CN) δ 5.94 (1H, ddt, *J* = 9.7, 2.5, 1.4 Hz, H1), 5.79 (1H, ddd, *J* = 9.7, 5.5, 2.3 Hz, H2), 3.66 (3H, s, H9), 3.48 (1H, ddt, *J* = 6.6, 3.1, 1.5 Hz, H3), 3.45 (1H, ddt, *J* = 10.8, 6.6, 2.2 Hz, H6), 2.55 (2H, m, H11), 2.49 (2H, m, H7), 2.28 (1H, m, H4), 2.11 (1H, m, H5), 1.57 (2H, m, H12), 1.49 (1H, td, *J* = 13.0, 10.8 Hz, H5), 1.34 (4H, m, H13/H14), 0.89 (3H, t, *J* = 7.2 Hz, H15). **^{13}C NMR (201 MHz, CD_3CN) δ** 172.6 (1C, C9), 134.8 (1C, C1), 123.0 (1C, C2), 119.4 (1C, C10), 52.3 (1C, C9), 40.8 (1C, C6), 38.6 (1C, C7), 34.1 (1C, C5), 33.6 (1C, C4), 31.8 (1C, C13), 31.5 (1C, C3), 30.3 (1C, C12), 30.1 (1C, C11), 22.9 (1C, C14), 14.3 (1C, C15). **HRMS (APCI$^+$, deconvoluted):** [M] calcd. for $C_{15}H_{23}NO_2S$, 281.1449; found, 281.1450.

Compound 23

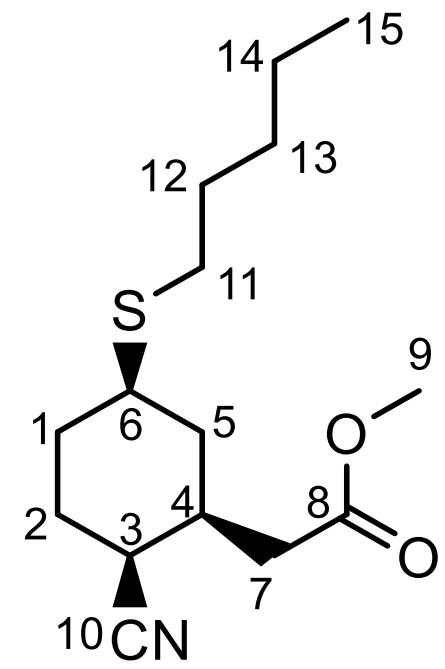

Compound **22** (7 mg; 0.025 mmol) was taken in iPrOH (15 mL) and reacted using a ThalesNano H-cube® Mini Plus (10% Pd/C,1 mL/min, 30°C, 28 atm) for 2 hr. The system was flushed with additional iPrOH (10 mL) and the combined solutions were evaporated to dryness in a weighed vial (6 mg; 0.021 mmol; 85%)

^{1}H NMR (600 MHz, CD_3CN) δ 3.64 (3H, s, H9), 3.10 (1H, dtd, *J* = 4.6, 3.1, 1.5 Hz, H3), 2.67 (1H, tt, *J* = 12.1, 3.8 Hz, H6), 2.54 (2H, m, H11), 2.41 (1H, dd, *J* = 16.1, 8.1 Hz, H7), 2.36 (1H, dd, *J* = 16.1, 6.5 Hz, H7), 2.10 (1H, m, H4), 2.00 (3H, m, H1/H2/H5), 1.68 (1H, m, H2), 1.55 (2H, dq, *J* = 8.9, 7.4 Hz, H12), 1.43 (1H, tdd, *J* = 13.6, 12.1, 3.9 Hz, H1), 1.34 (4H, m, H13/H14), 1.23 (1H, dt, *J* = 13.3, 12.2 Hz, H5), 0.89 (3H, t, *J* = 7.1 Hz, 15H). **^{13}C NMR (201 MHz, CD_3CN) δ** 172.7 (1C, C8), 120.8 (1C, C10), 52.3 (1C, C9), 41.9 (1C, C6), 39.4 (1C, C7), 36.9 (1C, C5), 36.3 (1C, C4), 32.5 (1C, C3), 31.8 (1C, C13), 30.5 (1C, C11/C12), 30.3 (1C, C1), 29.2 (1C, C2), 23.0 (1C, C14), 14.3 (1C, C15). **HRMS (APCI$^+$, deconvoluted):** [M] calcd. for $C_{15}H_{25}NO_2S$, 283.1606 found, 283.1607.

Compound 24

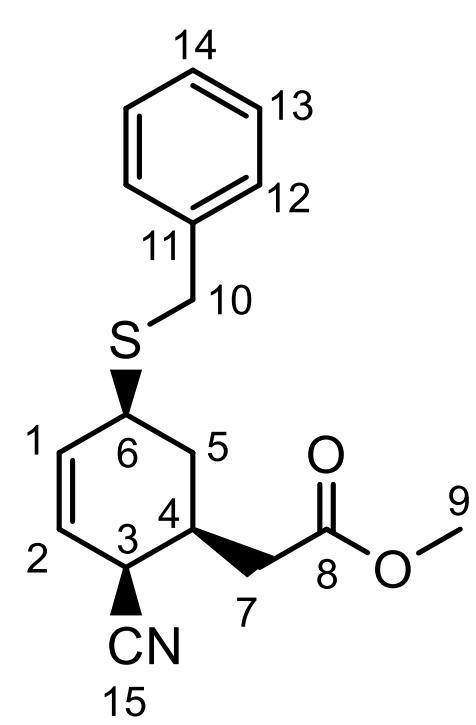

Compound **15** (100 mg; 0.124 mmol) was taken in MeCN (10 mL) and cooled to -30°C. To this a solution of DDQ (56.0 mg; 0.248) in 2 mL of ACN was added, and the combined reaction warmed to 25°C over 10 min. The reaction was then quenched with 15 mL saturated $NaHCO_3$(aq) and extracted with 15 mL DCM. The organic layer was dried over Na_2SO_4, evaporated to dryness, redissolved in minimal DCM (2 mL) and precipitated into 30 mL stirring hexane. The resulting solution was filtered through a fine porosity fritted disc, the remaining solids washed with Et_2O (2x3 mL) and the combined filtrates evaporated in vacuo. The product was further purified by flash chromatography (CombiFlash, 12g silica, Hex -> EtOAc) and compound **24** was collected from the 50% EtOAc/Hex portion into a weighed vial as a clear oil (29 mg; 0.096: 78%)

¹H NMR (600 MHz, Acetone) δ 7.38 (2H, m, H12), 7.32 (2H, m, H13), 7.24 (1H, m, H14), 5.95 (1H, ddt, *J* = 9.7, 2.4, 1.3 Hz, H1), 5.84 (1H, ddd, *J* = 9.7, 5.5, 2.3 Hz, H2), 3.85 (2H, s, H10), 3.67 (3H, s, H9), 3.59 (1H, m, H3), 3.45 (1H, ddt, *J* = 8.5, 6.2, 2.2 Hz, H6), 2.56 (1H, dd, *J* = 16.5, 8.0 Hz, H7), 2.51 (1H, dd, *J* = 16.5, 6.8 Hz, H7), 2.31 (1H, m, H4), 2.15 (1H, dddt, *J* = 13.4, 6.1, 2.7, 1.4 Hz, H5), 1.56 (1H, td, *J* = 13.0, 10.9 Hz, H5). **¹³C NMR (201 MHz, Acetone) δ** 172.2 (1C, C8), 139.5 (1C, C11), 134.1 (1C, C1), 129.9 (2C, C12), 129.3 (2C, C13), 127.8 (1C, C14), 123.3 (1C, C2), 118.9 (1C, C15), 51.9 (1C, C9), 40.8 (1C, C6), 38.4 (1C, C7), 34.7 (1C, C10), 33.8 (1C, C5), 33.5 (1C, C4), 31.4 (1C, C3). **HRMS (APCI⁺, deconvoluted):** [M] calcd. for $C_{17}H_{19}NO_2S$, 301.1136; found, 301.1135.

Compound 25

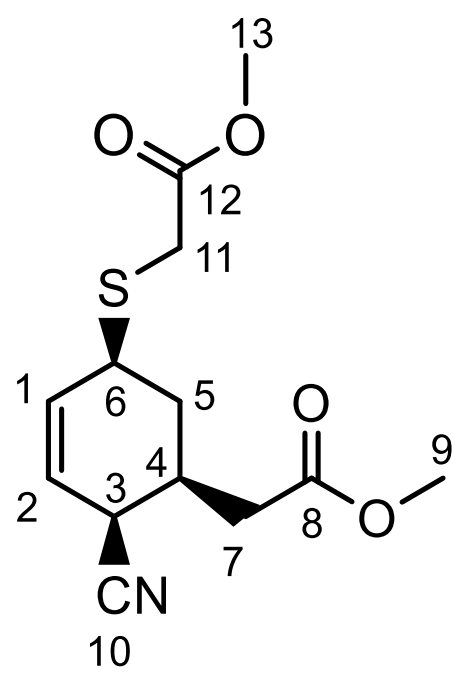

Compound **16** (95 mg; 0.121 mmol) taken in DCM (2 mL) and cooled to -30°C. Solid CAN (73 mg; 0.133 mmol) was added to this followed by cold (-30°C) ACN (4mL) and the mixture was stirred 1 hr. The reaction was quenched with 10 mL 5% Na_2SO_3(aq) and extracted with DCM (15 mL). The organic layer was dried over Na_2SO_4, evaporated to dryness, and further purified by flash chromatography (CombiFlash, 4g silica, Hex -> 25% EtOAc/Hex -> EtOAC). Compound **25** was collected from the 25% EtOAc/Hex portion into a weighed vial, and evaporated to a clear oil (14 mg; 0.049 mmol; 41%).

¹H NMR (600 MHz, CD_3CN) δ 5.94 (1H, ddt, *J* = 9.8, 2.5, 1.3 Hz, H1), 5.83 (1H, ddd, *J* = 9.8, 5.5, 2.3 Hz, H2), 3.67 (3H, s, H13), 3.66 (3H, s, H9), 3.59 (1H, ddq, *J* = 10.8, 6.5, 2.1 Hz, H6), 3.50 (1H, tq, *J* = 5.3, 1.7 Hz, H3), 3.33 (2H, d, *J* = 2.6 Hz, H11), 2.50 (2H, m, H7), 2.30 (1H, m, H4), 2.14 (1H, m, H5), 1.48 (1H, td, *J* = 13.1, 10.9 Hz, H5). **¹³C NMR (201 MHz, CD_3CN) δ** 172.5 (1C, C8), 171.8 (1C, C12), 133.6 (1C, C1), 124.0 (1C, C2), 119.2 (1C, C10), 52.9 (1C, C13), 52.4 (1C, C9), 41.5 (1C, C6), 38.5 (1C, C7), 33.6 (1C, C5), 33.5 (1C, C4), 32.3 (1C, C11), 31.4 (1C, C3). **HRMS (APCI⁺, deconvoluted):** [M] calcd. for $C_{13}H_{17}NO_4S$, 283.0878; found, 283.0881.

Compound 28

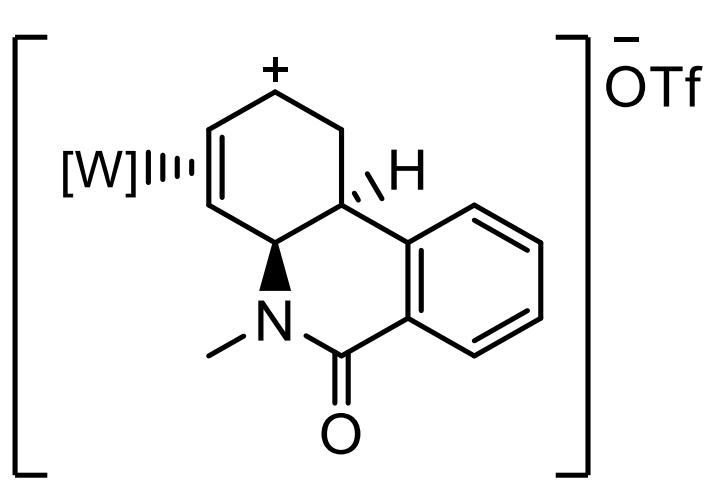

Compound **27** (134 mg; 0.188 mmol) was taken in MeCN (4 mL) and cooled to -30°C. To this, 1M HOTf (413 µL) was added and the reaction stirred cold for 30 min. The crude reaction mixture was then precipitated into chilled (-10°C) Et_2O (50 mL) and the precipitate collected on a fine porosity fritted disc to yield Compound **28** as a white powder (155 mg; 0.179 mmol; 95%)

^{1}H NMR (600 MHz, CD_3CN) δ 8.48 (d, *J* = 2.3 Hz, 1H), 8.07 (dd, *J* = 7.9, 1.3 Hz, 1H), 7.99 (dd, *J* = 2.3, 0.6 Hz, 1H), 7.98 (dt, *J* = 2.5, 0.8 Hz, 1H), 7.97 (d, *J* = 2.4 Hz, 1H), 7.87 (d, *J* = 2.4 Hz, 1H), 7.82 (m, 2H), 7.68 (dt, *J* = 8.1, 1.2 Hz, 1H), 7.56 (tt, *J* = 7.6, 1.0 Hz, 1H), 6.56 (t, *J* = 2.4 Hz, 1H), 6.50 (t, *J* = 2.4 Hz, 1H), 6.35 (t, *J* = 2.4 Hz, 1H), 5.66 (ddd, *J* = 7.4, 2.5, 1.3 Hz, 1H), 5.18 (dd, *J* = 7.6, 6.6 Hz, 1H), 5.03 (dq, *J* = 6.1, 1.3 Hz, 1H), 4.57 (dt, *J* = 14.9, 7.4 Hz, 1H), 3.77 (ddt, *J* = 16.6, 7.7, 1.8 Hz, 1H), 3.70 (s, 1H), 3.65 (s, 3H), 2.72 (ddd, *J* = 16.9, 5.1, 2.1 Hz, 1H), 1.24 (d, *J* = 10.0 Hz, 9H).

Compound 29

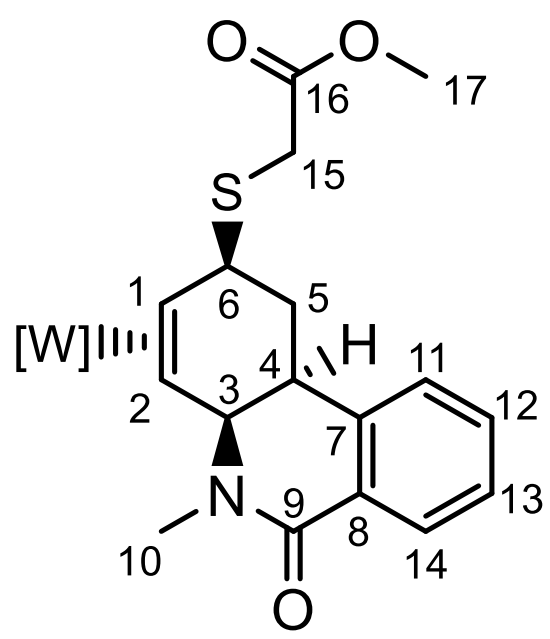

Compound **28** (73 mg; 0.085 mmol) was taken in MeCN (3 mL) and cooled to -30°C. $KSCH_2CO_2CH_3$ (24 mg; 0.169 mmol) was taken in MeCN (1.5 mL) with 5 drops of MeOH to solubilize the salt, and cooled to -30°C. The two solutions were combined and stirred cold for 30 min. The crude reaction was then evaporated under reduced pressure, redissolved in DCM (2.5 mL) and precipitated into stirring pentane (30 mL). The precipitate was collected on a medium porosity fritted disc and washed with DCM (2 mL) back into the pentane, forming a precipitate. This precipitate was collected on a fine porosity fritted disc to yield compound **29** as a white powder (26 mg; 0.032 mmol; 38%)

^{1}H NMR (600 MHz, CD_2Cl_2) δ 8.11 (1H, d, *J* = 2.1 Hz, Tp3B), 8.00 (1H, dd, *J* = 7.7, 1.5 Hz, H14), 7.78 (2H, m, Tp5B/C), 7.65 (1H, dt, *J* = 2.4, 0.8 Hz, Tp5A), 7.63 (1H, d, *J* = 2.1 Hz, Tp3A), 7.43 (1H, td, *J* = 7.4, 1.4 Hz, H12), 7.35 (1H, d, *J* = 2.2 Hz, Tp3C), 7.31 (2H, m, H11/H13), 6.36 (1H, t, *J* = 2.2 Hz, Tp4B), 6.30 (1H, t, *J* = 2.2 Hz, Tp4C), 6.12 (1H, t, *J* = 2.2 Hz, Tp4A), 4.56 (1H, m, H3), 4.38 (1H, t, *J* = 8.1 Hz, H6), 3.69 (4H, s, H4/H17), 3.37 (1H, d, *J* = 14.9 Hz, H15), 3.26 (1H, d, *J* = 14.9 Hz, H15), 3.15 (3H, s, H10), 2.67 (1H, ddd, *J* = 14.6, 10.9, 1.6 Hz, H1), 2.04 (1H, ddd, *J* = 13.4, 7.6, 4.5 Hz, H5), 1.86 (1H, td, *J* = 12.8, 8.6 Hz, H5), 1.47 (1H, ddd, *J* = 10.9, 2.5, 1.2 Hz, H2), 1.30 (9H, d, *J* = 8.5 Hz, PMe_3). **^{13}C NMR (201 MHz, CD_2Cl_2) δ** 171.7 (1C, C16), 166.0 (1C, C9), 144.9 (1C, C7), 144.1 (1C, Tp3A), 143.5 (1C, Tp3B), 140.5 (1C, Tp3C), 137.1 (1C, Tp5B/C), 136.7 (1C, Tp5B/C), 136.6 (1C, Tp5A), 131.8 (1C, C12), 129.1 (1C, C8), 128.5 (1C, C14), 127.9 (1C, C11), 126.9 (1C, C13), 107.0 (1C, Tp4B), 106.7 (1C, Tp4C), 106.3 (1C, Tp4A), 59.2 (1C, C3), 52.5 (1C, C17), 50.2 (1C, d, *J* = 10.6 Hz, C1), 43.0 (1C, C6), 36.1 (1C, C4), 35.1 (1C, C5), 32.6 (1C, C15), 29.8 (1C, C10), 14.3 (3C, d, *J* = 27.9 Hz, PMe_3). *C2 under CD_2Cl_2 ~53.7 ppm.* **HRMS ($APCI^+$, deconvoluted):** [M] calcd. for $C_{29}H_{38}BN_8O_4PSW$, 820.2082; found, 820.2091.

Compound 30

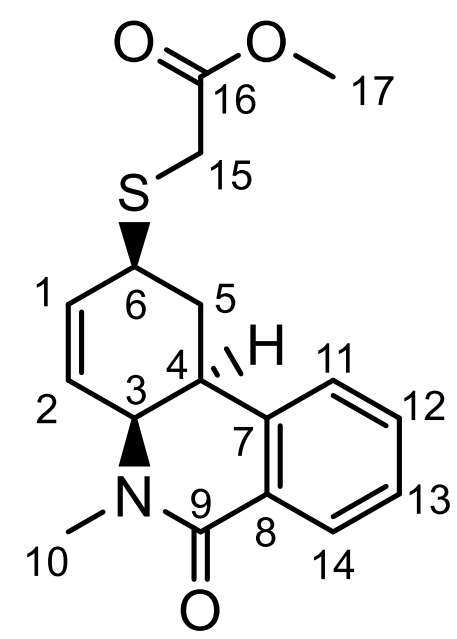

Compound **28** (150 mg; 0.174 mmol) was taken in MeCN (5 mL) and cooled to -30°C. $KSCH_2CO_2CH_3$ (50 mg; 0.347 mmol) was taken in MeCN (3 mL) with 10 drops of MeOH to solubilize the salt, and cooled to -30°C. The two solutions were combined and stirred cold for 20 min. The crude reaction was then precipitated into stirring Et_2O (30 mL) and filtered through a medium porosity fritted disc. The precipitate was washed with DCM (2x2 mL) and the combined filtrate was evaporated to dryness to yield compound **29** as a crude film. This was then redissolved in DCM (2 mL) and cooled to -30°C. To this, CAN (105 mg; 0.191 mmol) in MeCN (3 mL) at -30°C was added and the mixture was stirred 10 min. The reaction was quenched with 15 mL 10% Na_2SO_3(aq) and extracted with DCM (3x10 mL). The organic layer was dried over Na_2SO_4, evaporated to dryness, and further purified by flash chromatography (4g silica, Hex -> 80% EtOAc/Hex gradient). Compound **30** was collected from the 50% EtOAc/Hex portion into a weighed vial, and evaporated to a clear oil (20 mg; 0.063 mmol; 36%).

^{1}H NMR (600 MHz, CD_3CN) δ 7.96 (1H, ddt, *J* = 7.8, 1.1, 0.5 Hz, H14), 7.49 (1H, td, *J* = 7.5, 1.4 Hz, H12), 7.36 (1H, td, *J* = 7.6, 1.3 Hz, H13), 7.31 (1H, ddt, *J* = 7.6, 1.3, 0.6 Hz, H11), 6.12 (1H, ddd, *J* = 10.1, 5.3, 2.1 Hz, H2), 6.07 (1H, m, H1), 4.21 (1H, t, *J* = 4.6 Hz, H3), 3.70 (1H, m, H6), 3.64 (3H, s, H17), 3.29 (2H, s, H15), 3.07 (1H, ddd, *J* = 12.7, 4.4, 3.2 Hz, H6), 3.03 (3H, s, H10), 2.02 (1H, dddt, *J* = 13.3, 6.4, 3.0, 1.2 Hz, H5), 1.86 (1H, td, *J* = 13.0, 10.6 Hz, H5). **^{13}C NMR (201 MHz, CD_3CN) δ** 172.0 (1C, C16), 164.6 (1C, C9), 142.5 (1C, C7), 134.5 (1C, C1), 133.0 (1C, C12), 128.9 (1C, C8), 128.8 (1C, C14), 128.3 (1C, C11), 128.1 (1C, C13), 127.8 (1C, C2), 52.9 (1C, C17), 52.6 (1C, C3), 42.0 (1C, C6), 38.8 (1C, C4), 33.5 (1C, C5), 32.0 (1C, C15), 30.5 (1C, C10). **HRMS (APCI$^+$, deconvoluted):** [M] calcd. for $C_{17}H_{19}NO_3S$, 317.1086; found, 317.1085.

NMR Spectra

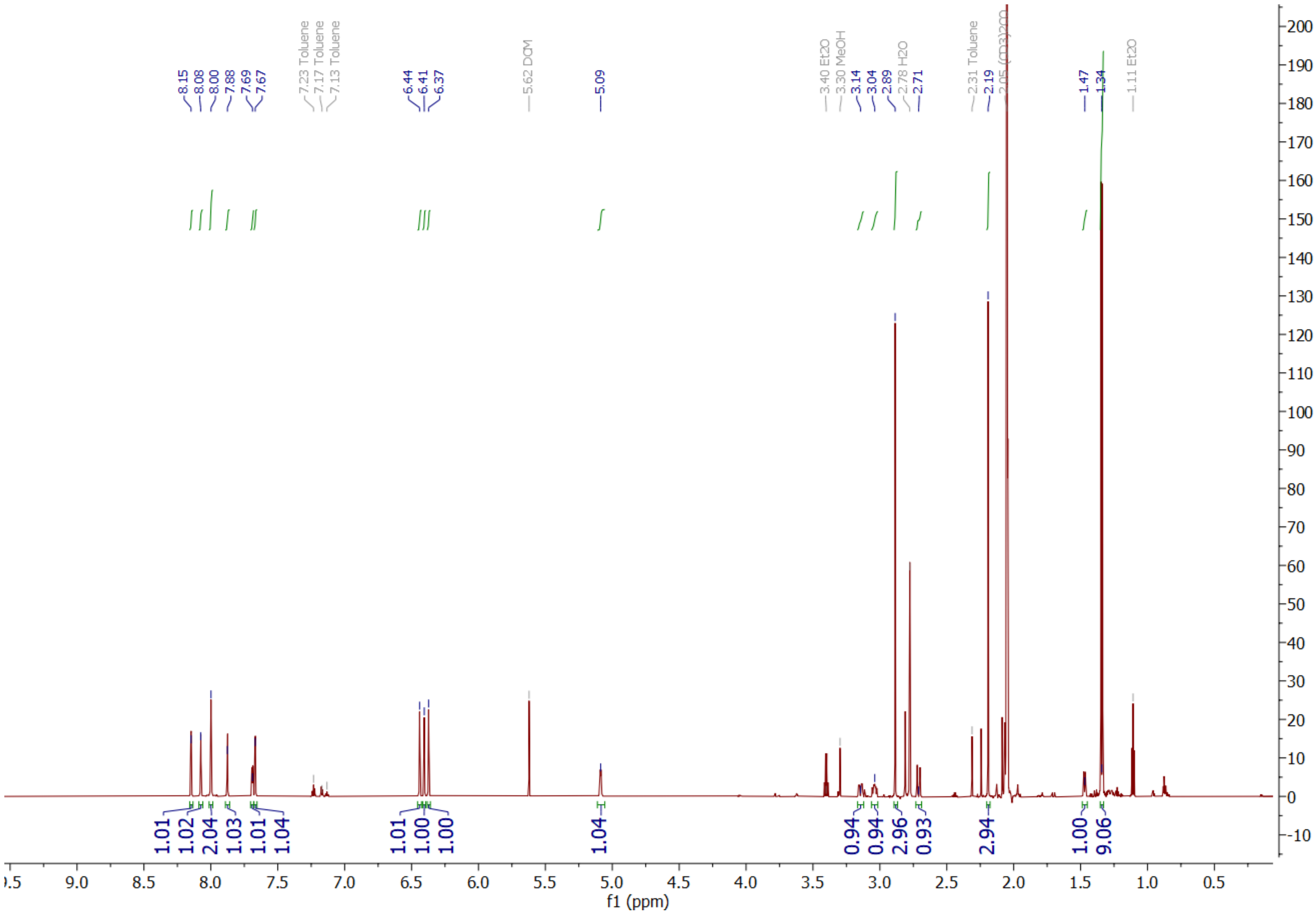

Supplementary Figure 2. ^{1}H NMR ($(CD_3)_2CO$) of Compound **2.**

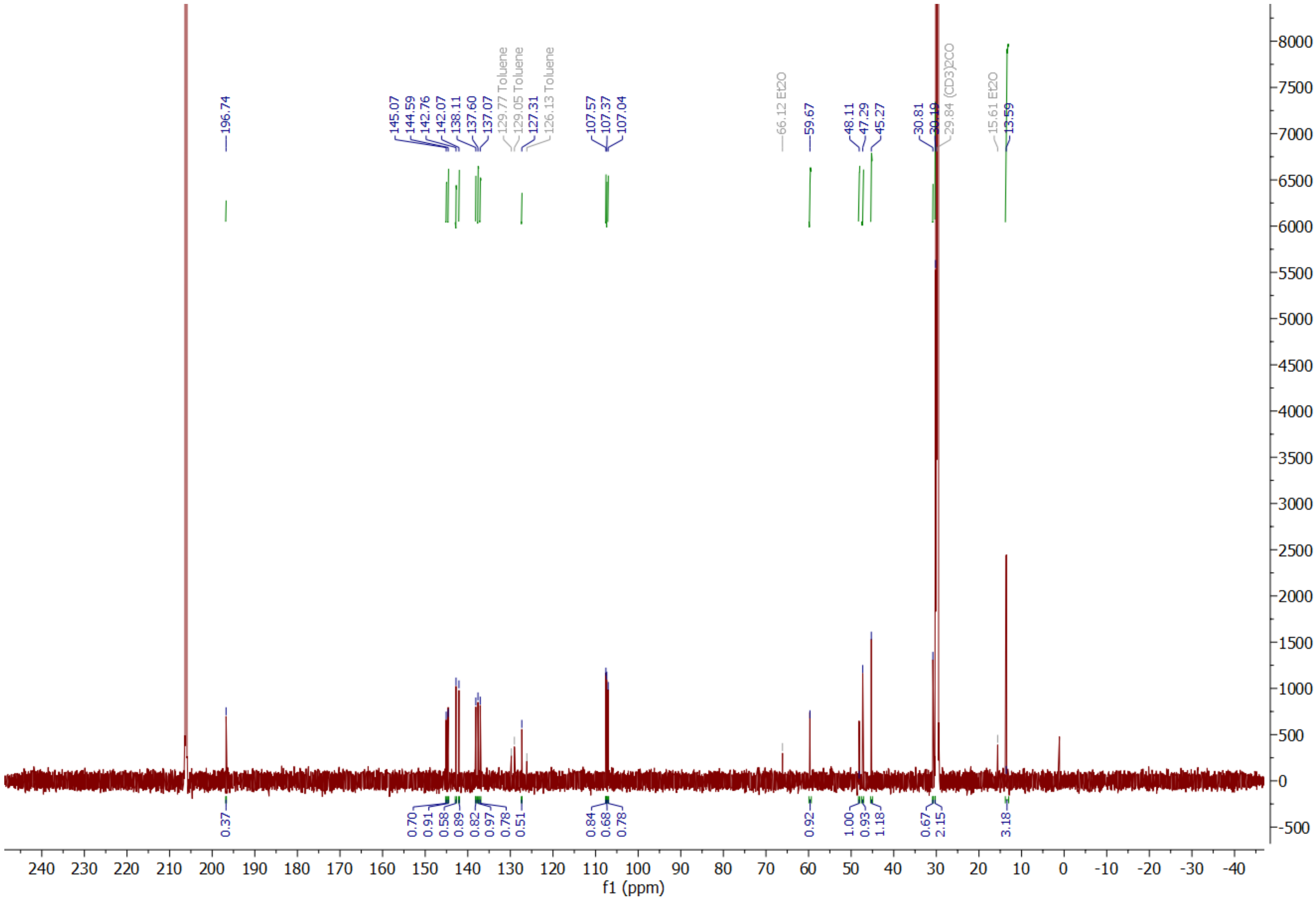

Supplementary Figure 3. ^{13}C NMR ($(CD_3)_2CO$) of Compound **2.**

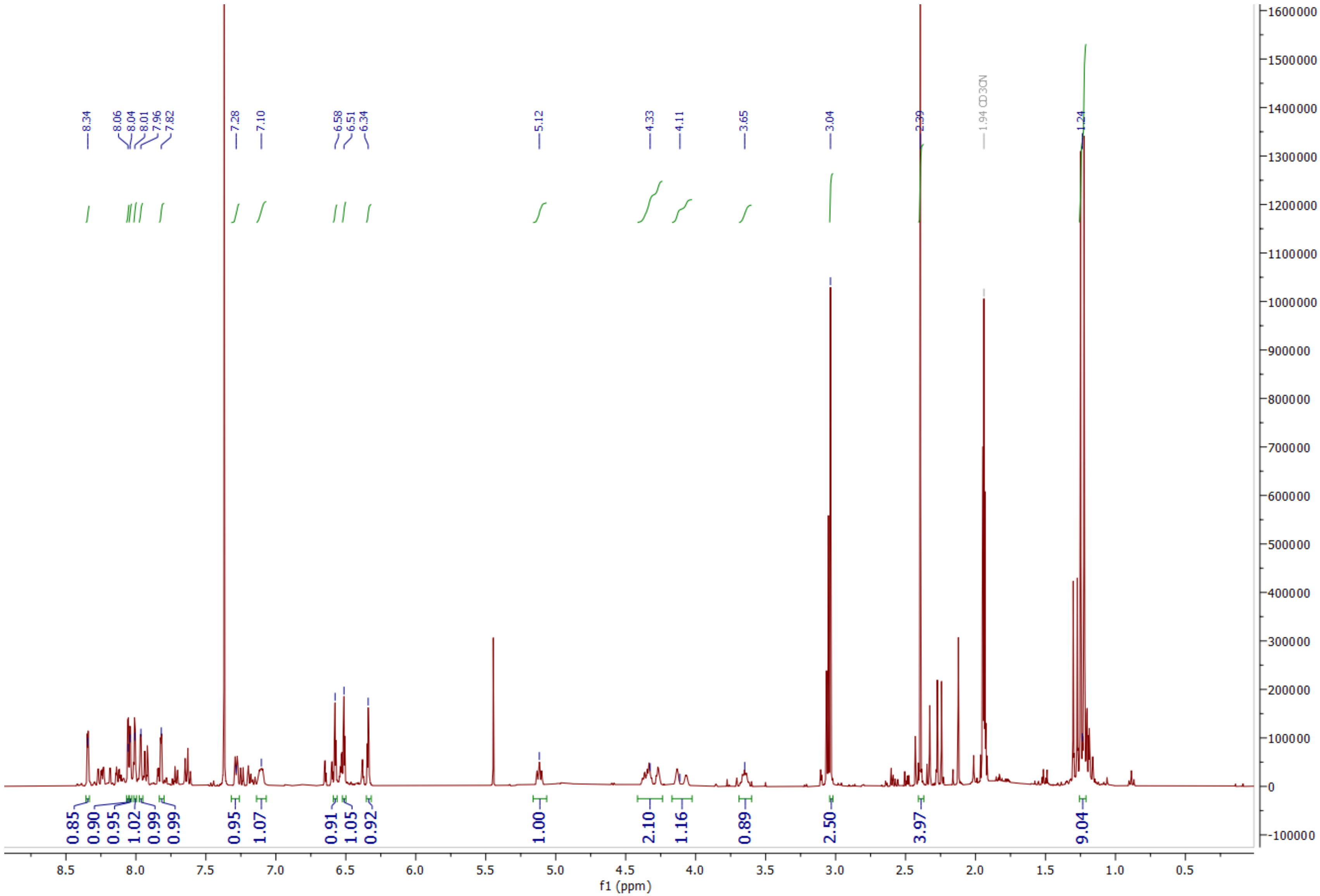

Supplementary Figure 4. ^{1}H NMR (CD_3CN) of Compound **3.** Unknown decomposition peaks can be seen growing in ~6.3 – 8.4 ppm and 1.29 ppm as the sample warms above -30°C.

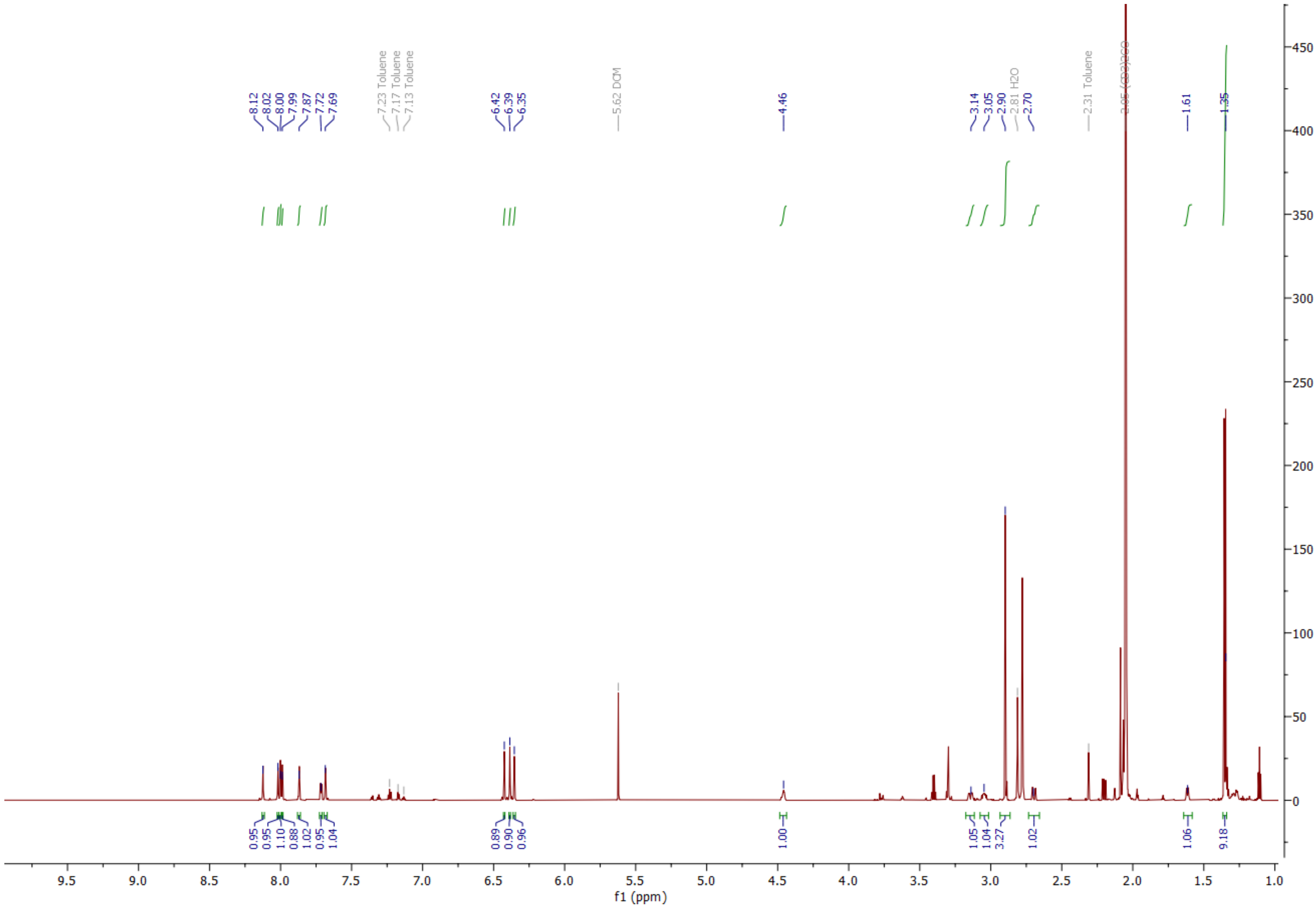

Supplementary Figure 5. ^{1}H NMR ($(CD_3)_2CO$) of Compound **4**.

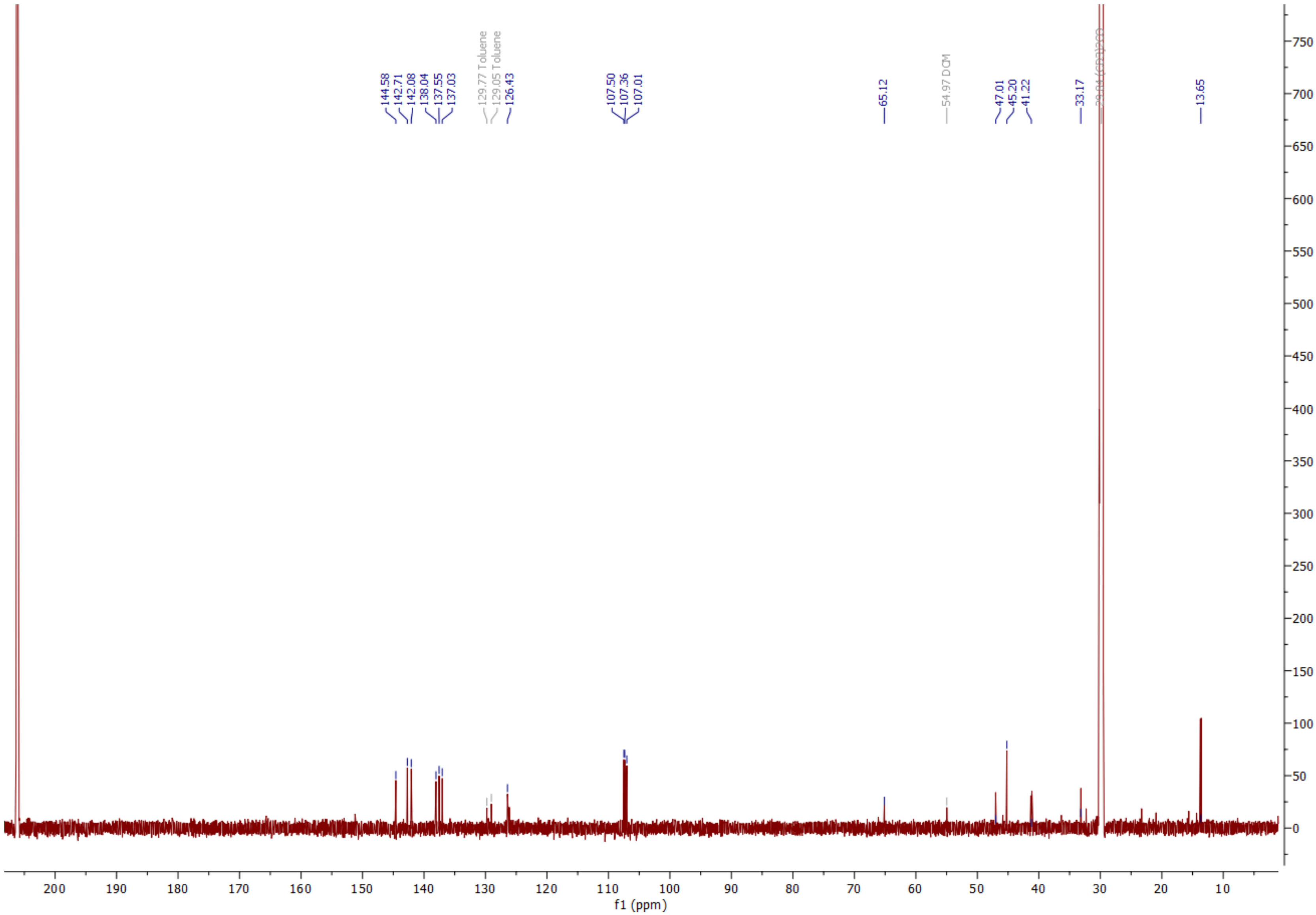

Supplementary Figure 6. ^{13}C NMR ($(CD_3)_2CO$) of Compound **4**.

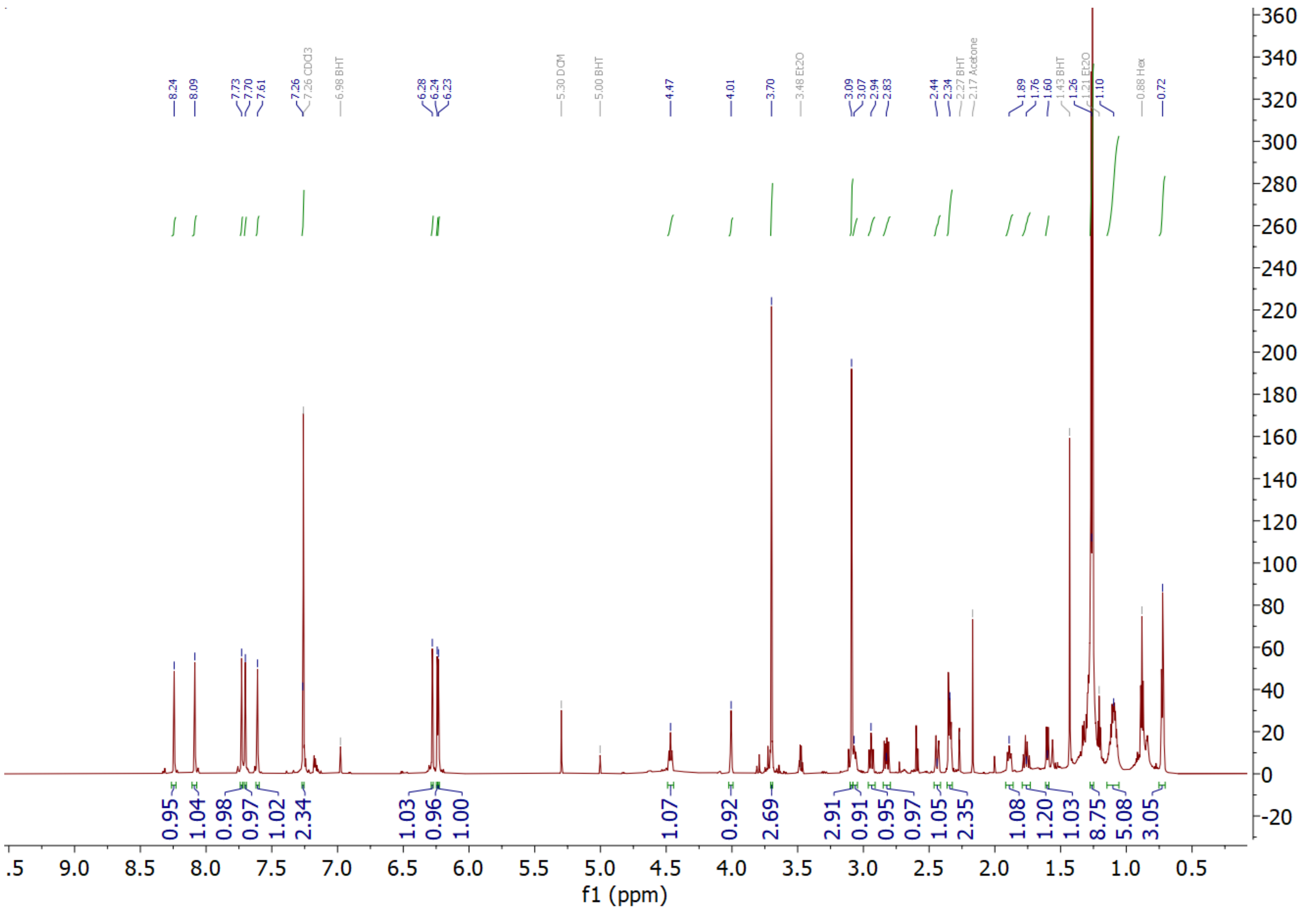

Supplementary Figure 7. ^{1}H NMR (CD_3Cl) of Compound **7.**

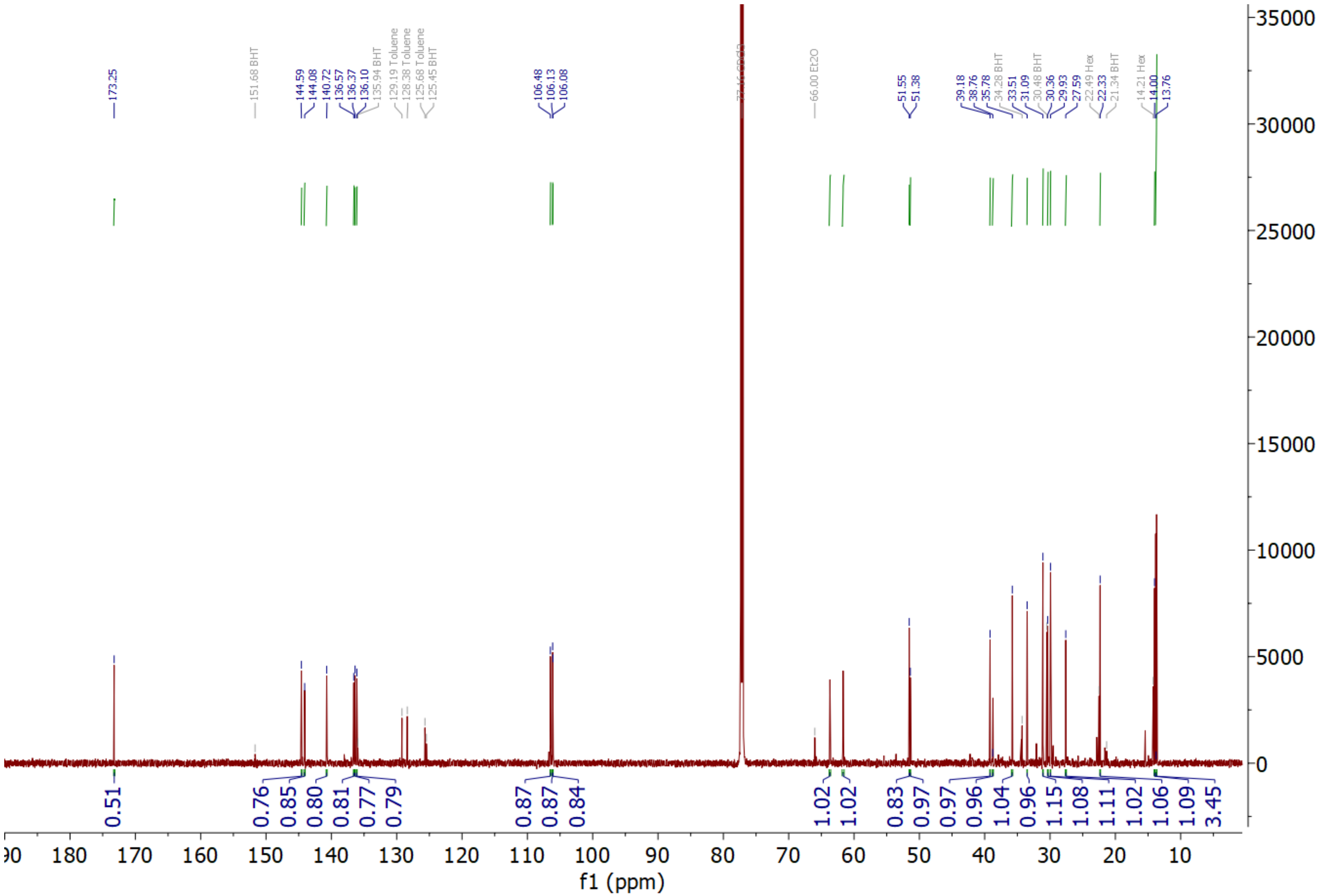

Supplementary Figure 8. ^{13}C NMR (CD_3Cl) of Compound **7.**

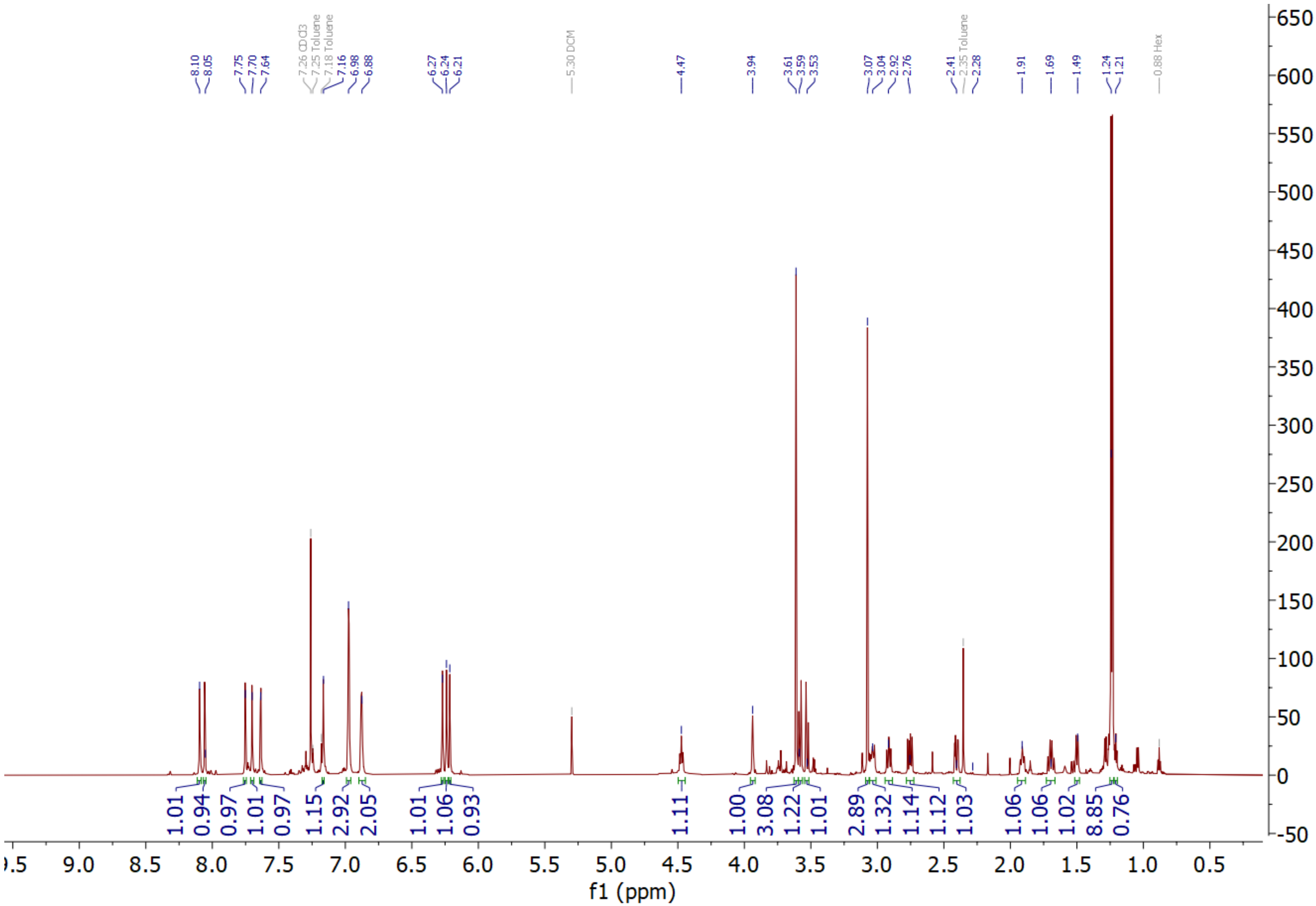

Supplementary Figure 9. ^{1}H NMR (CD_3Cl) of Compound **8.**

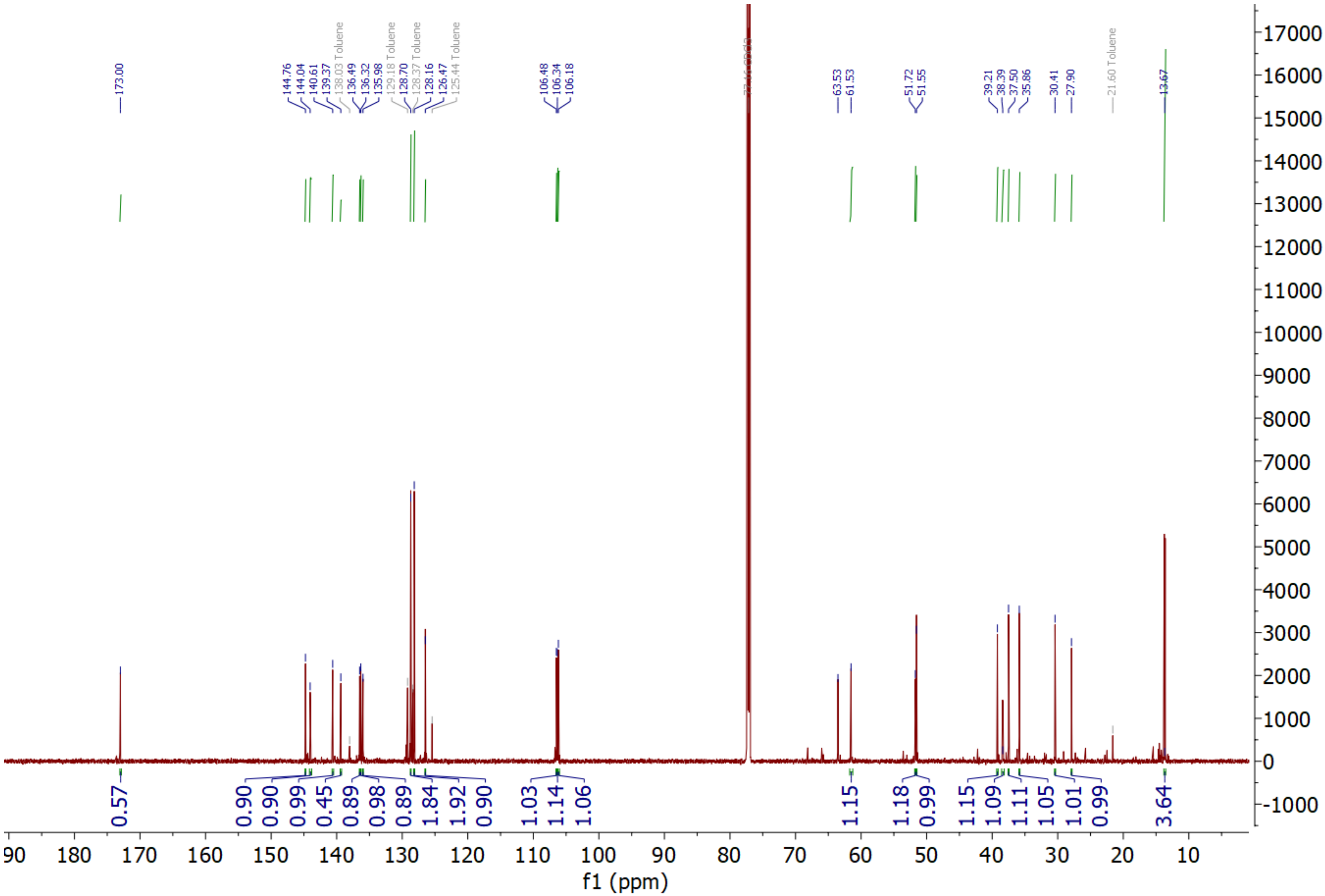

Supplementary Figure 10. ^{13}C NMR (CD_3Cl) of Compound **8.**

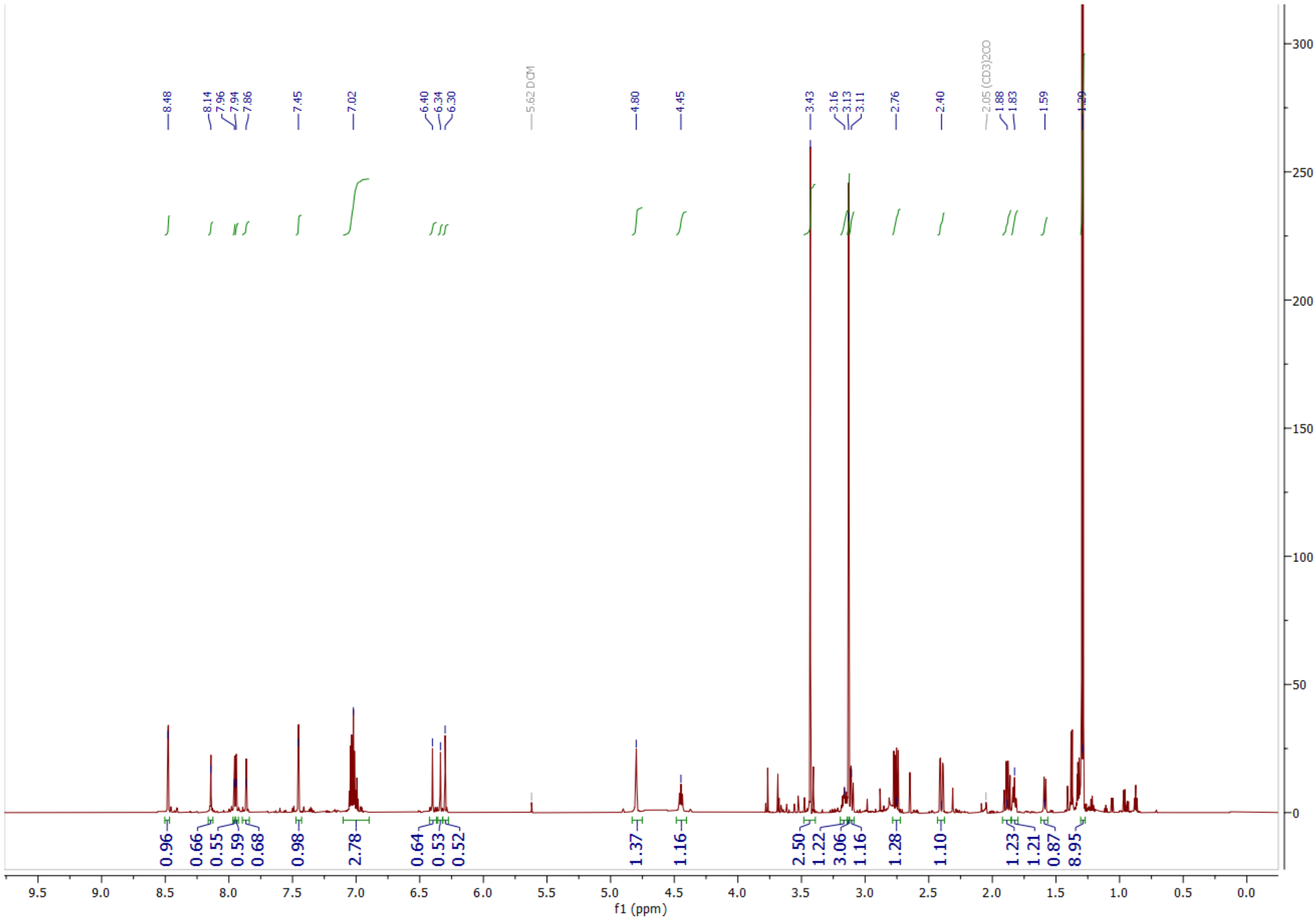

Supplementary Figure 11. ^{1}H NMR ($(CD_3)_2CO$) of Compound **9.**

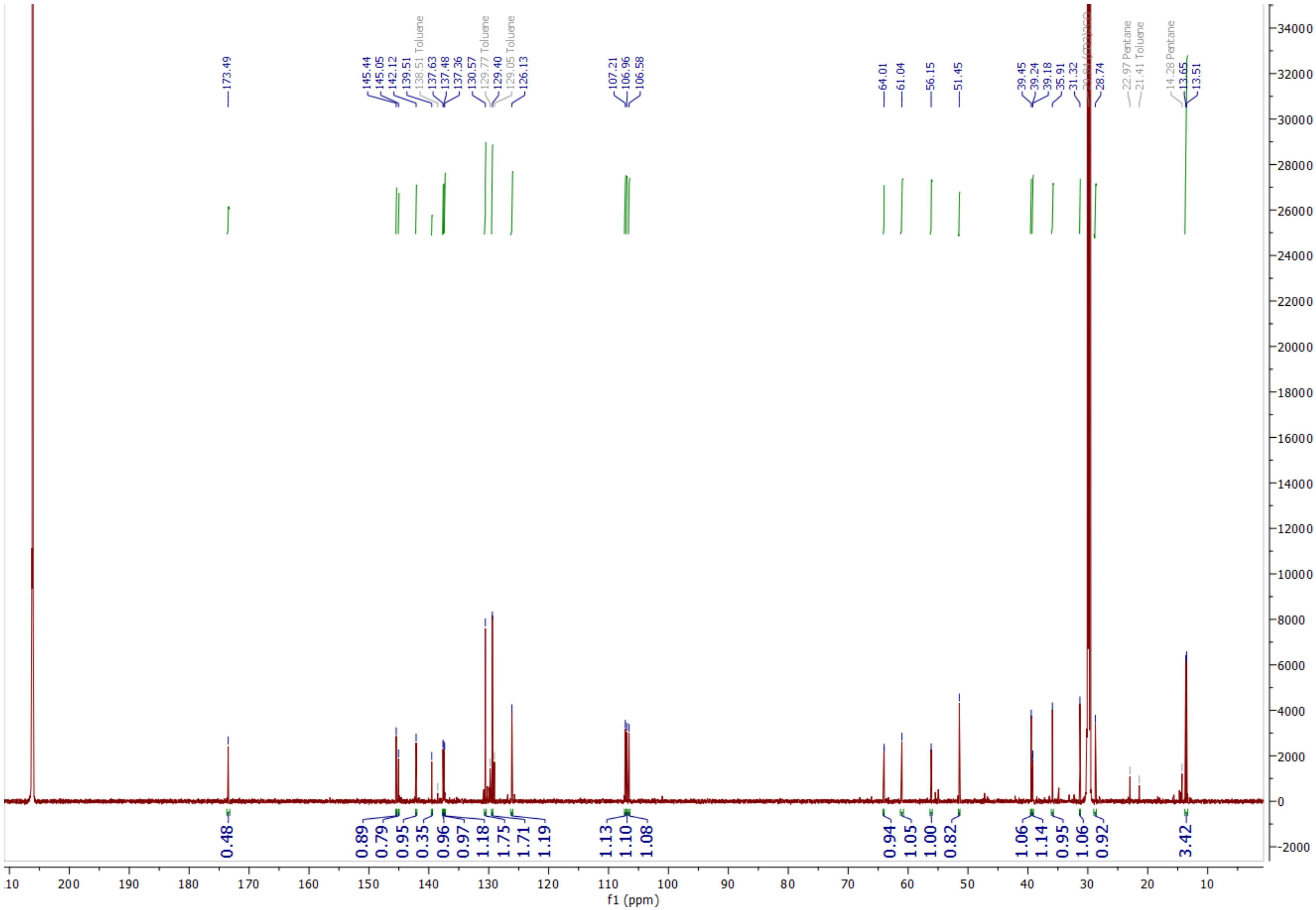

Supplementary Figure 12. ^{13}C NMR ($(CD_3)_2CO$) of Compound **9.**

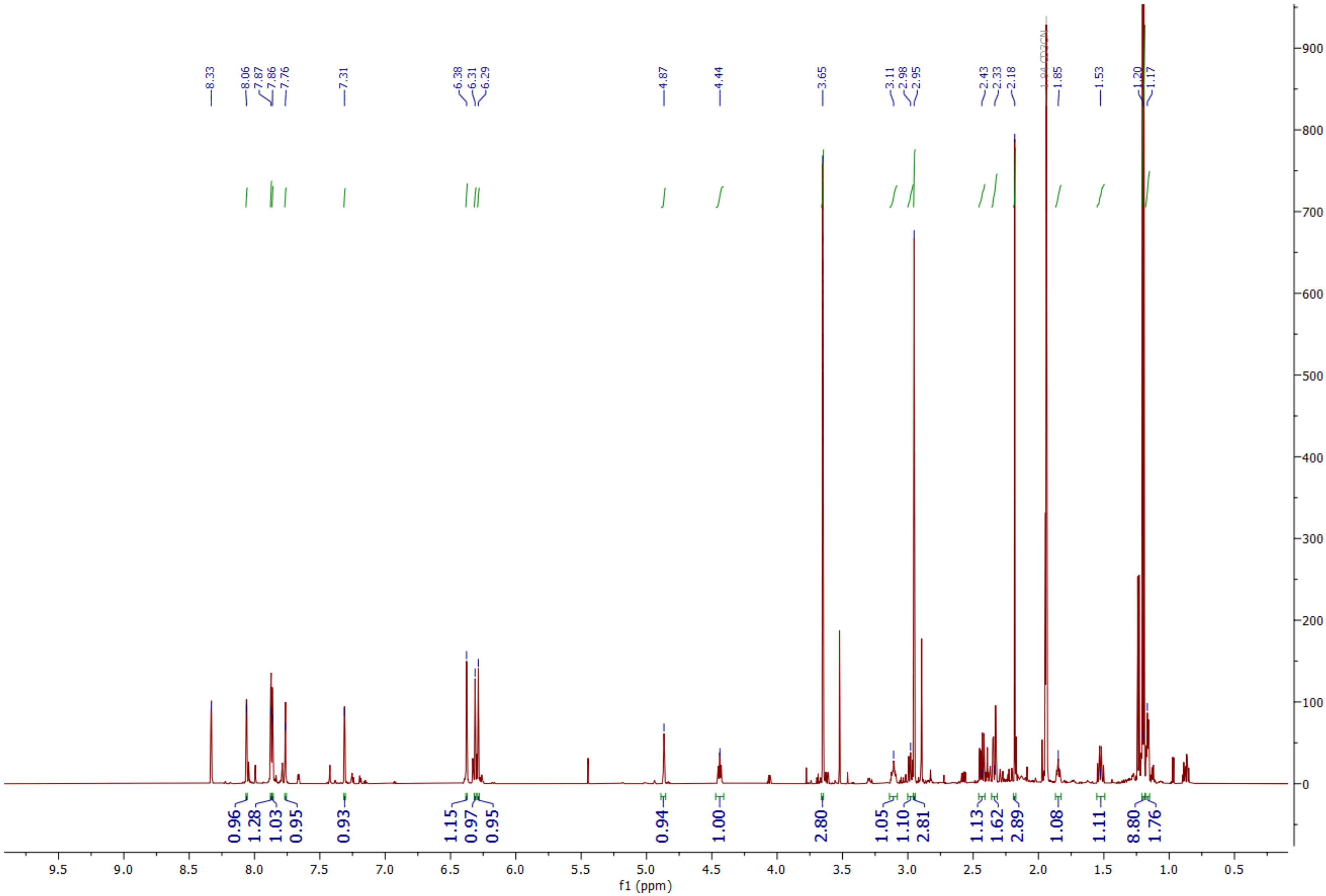

Supplementary Figure 13. ^{1}H NMR (CD_3CN) of Compound **10** (with ~0.15 residual Compound **5**).

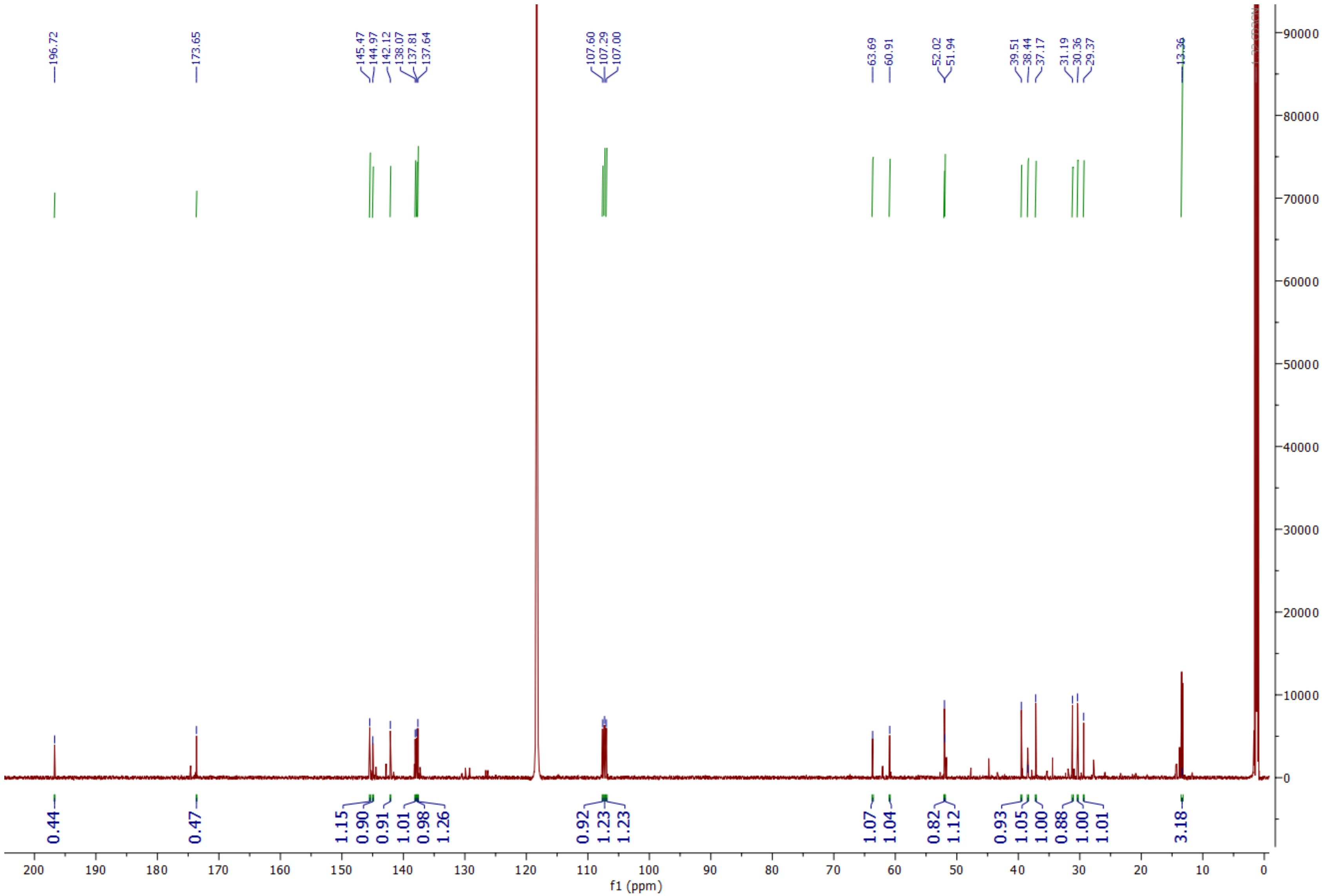

Supplementary Figure 14. ^{13}C NMR (CD_3CN) of Compound **10** (with ~0.15 residual Compound **5**).

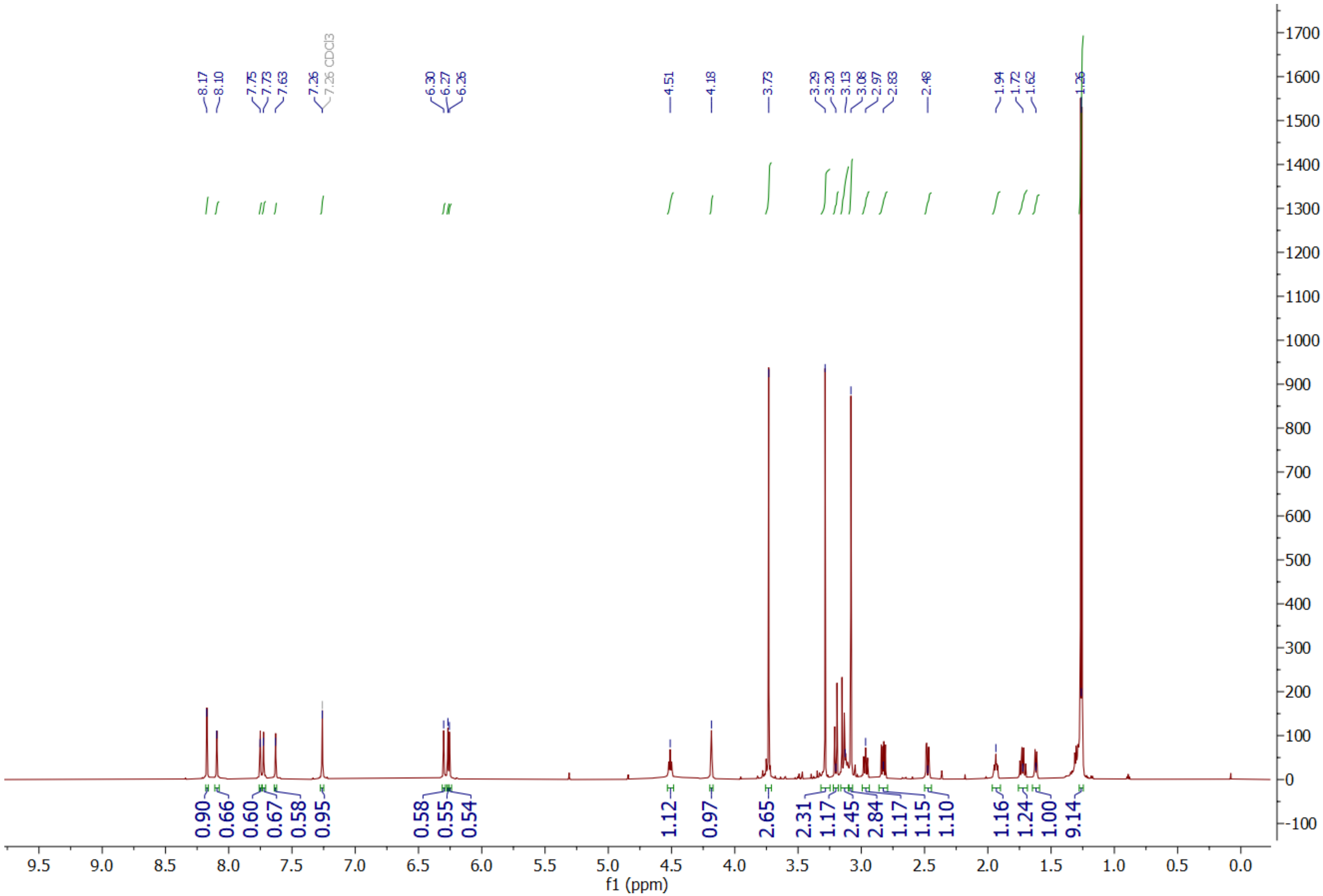

Supplementary Figure 15. ^{1}H NMR (CD_3Cl) of Compound **11.**

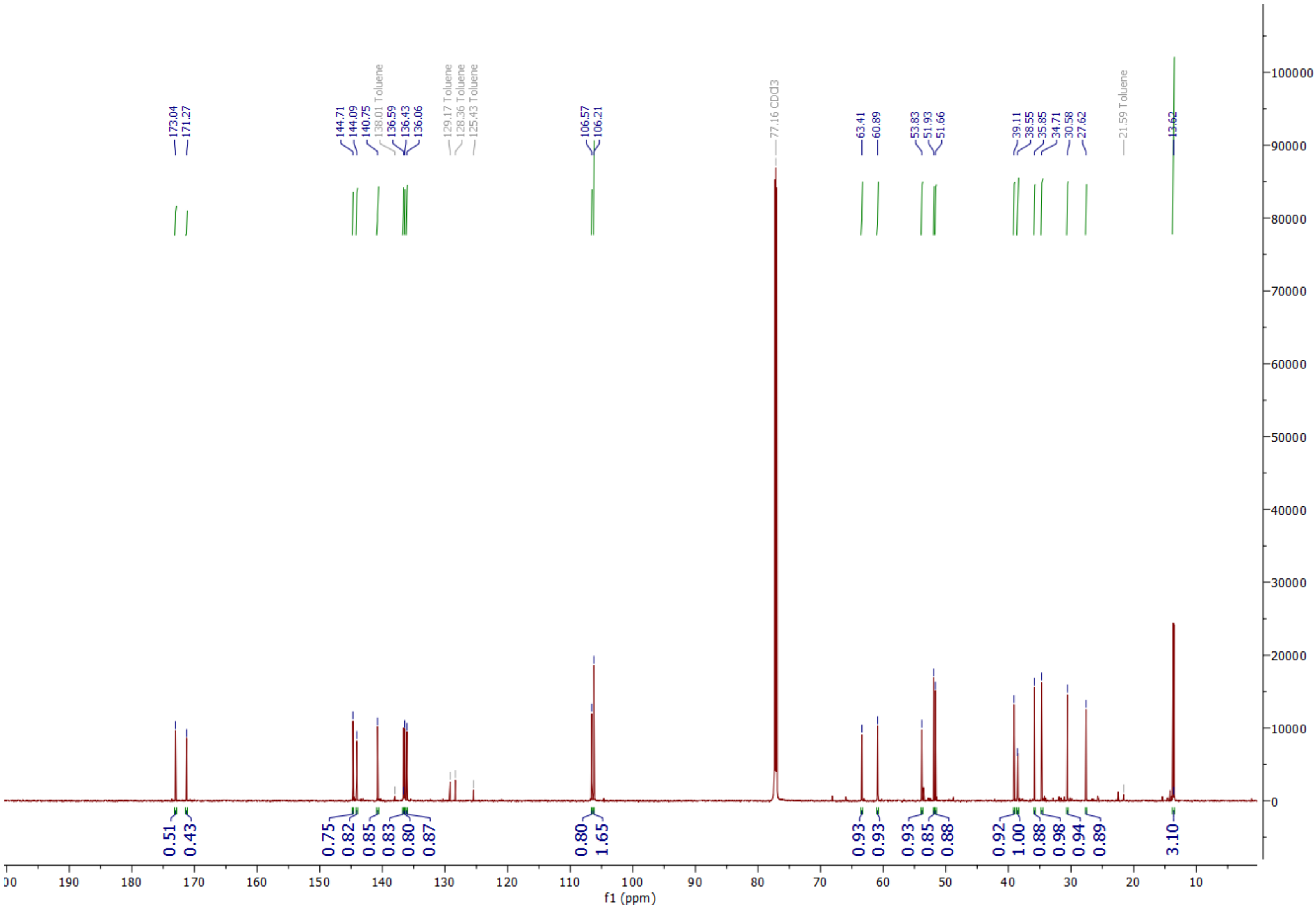

Supplementary Figure 16. ^{13}C NMR (CD_3Cl) of Compound **11.**

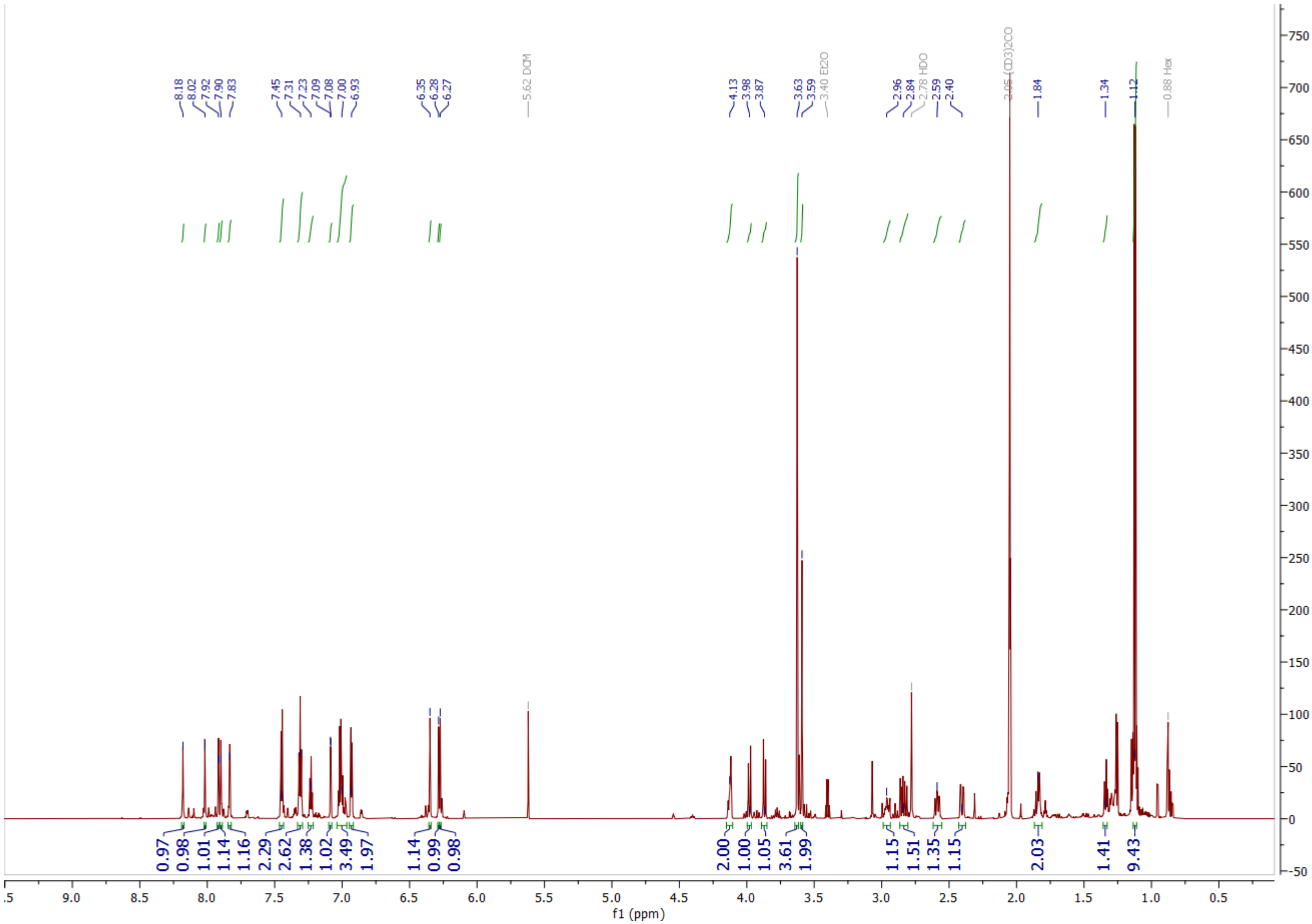

Supplementary Figure 17. ¹H NMR ($(CD_3)_2CO$) of Compound **13**.

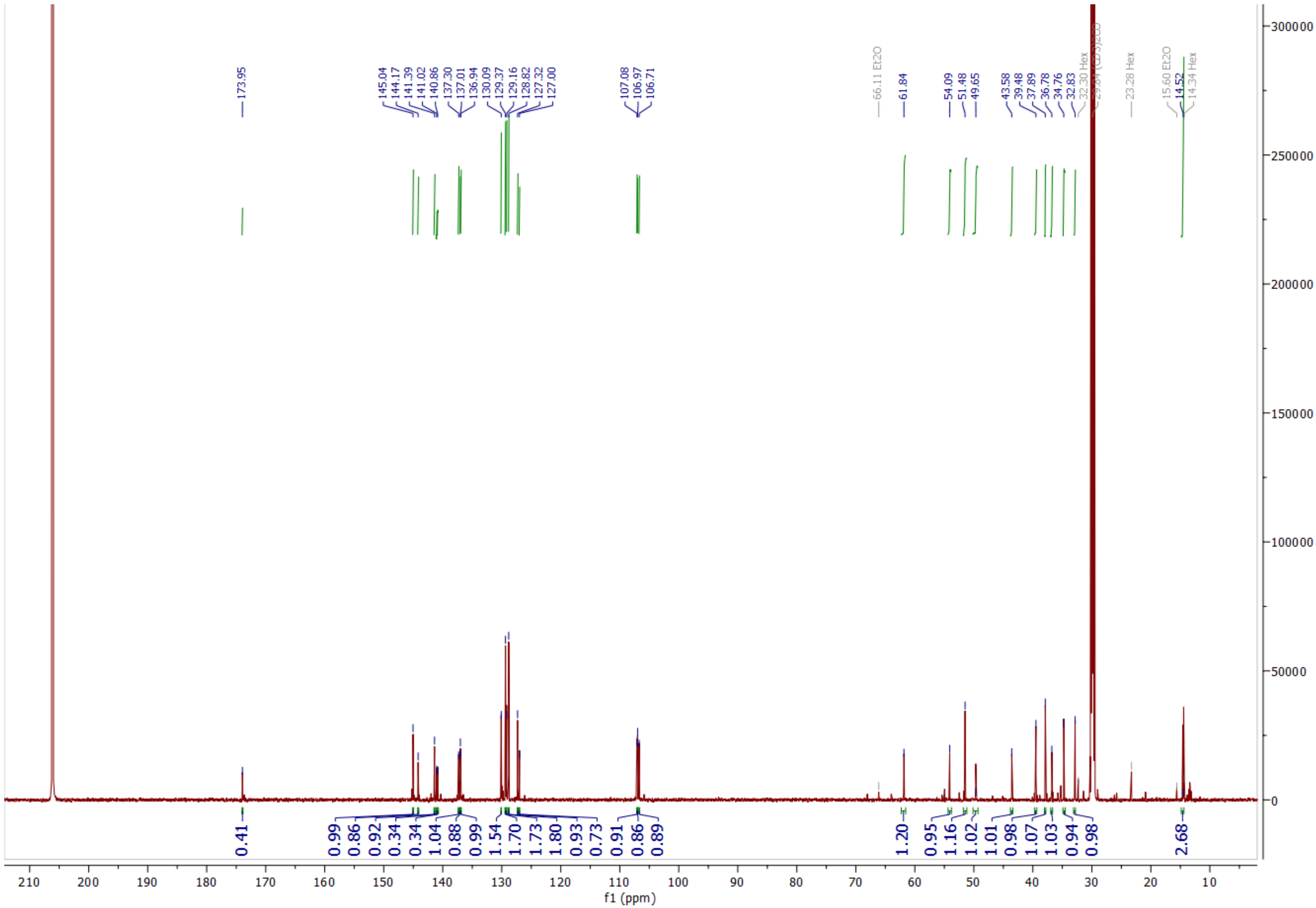

Supplementary Figure 18. ^{13}C NMR ($(CD_3)_2CO$) of Compound **13**.

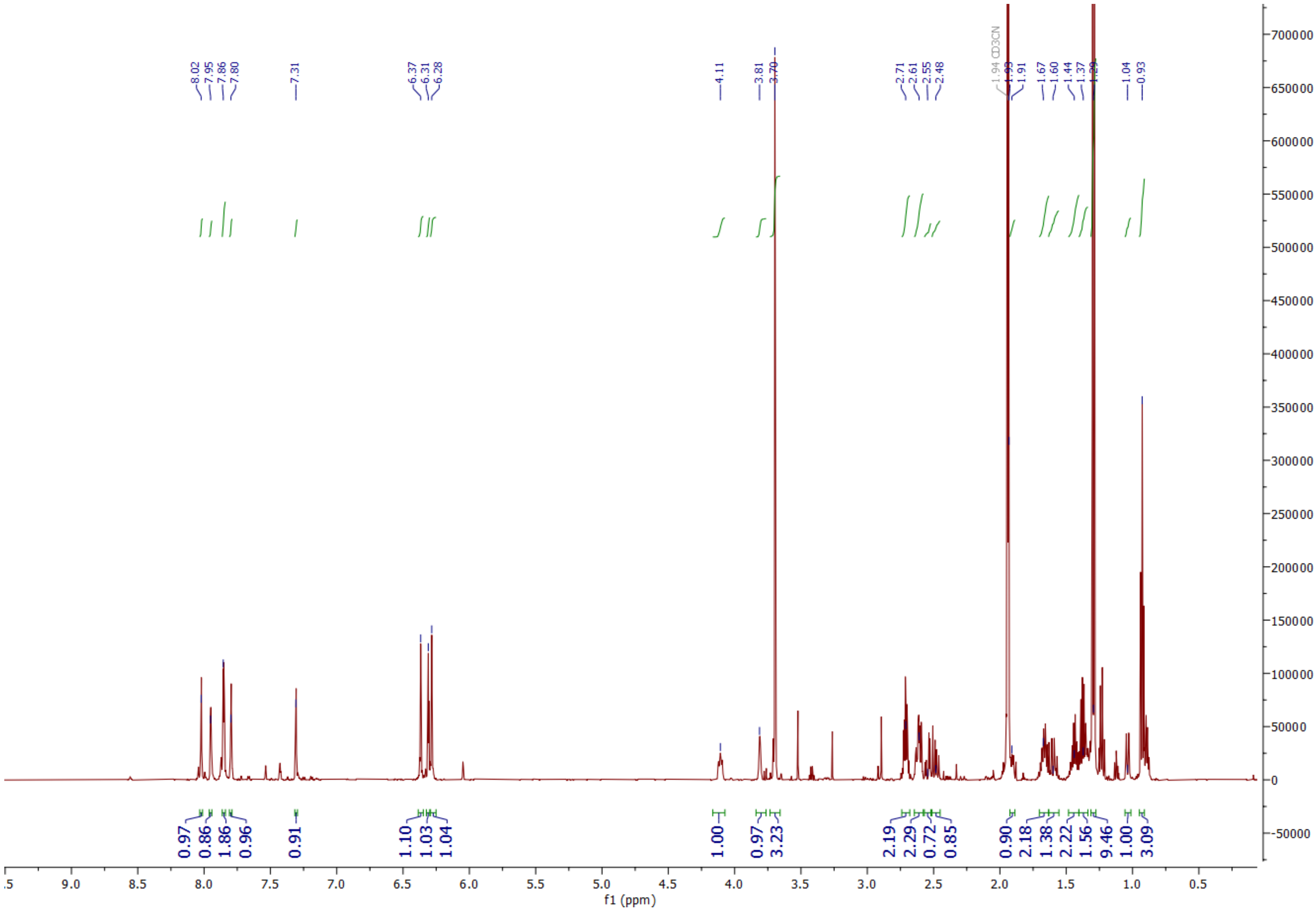

Supplementary Figure 19. ^{1}H NMR (CD_3Cl) of Compound **14.**

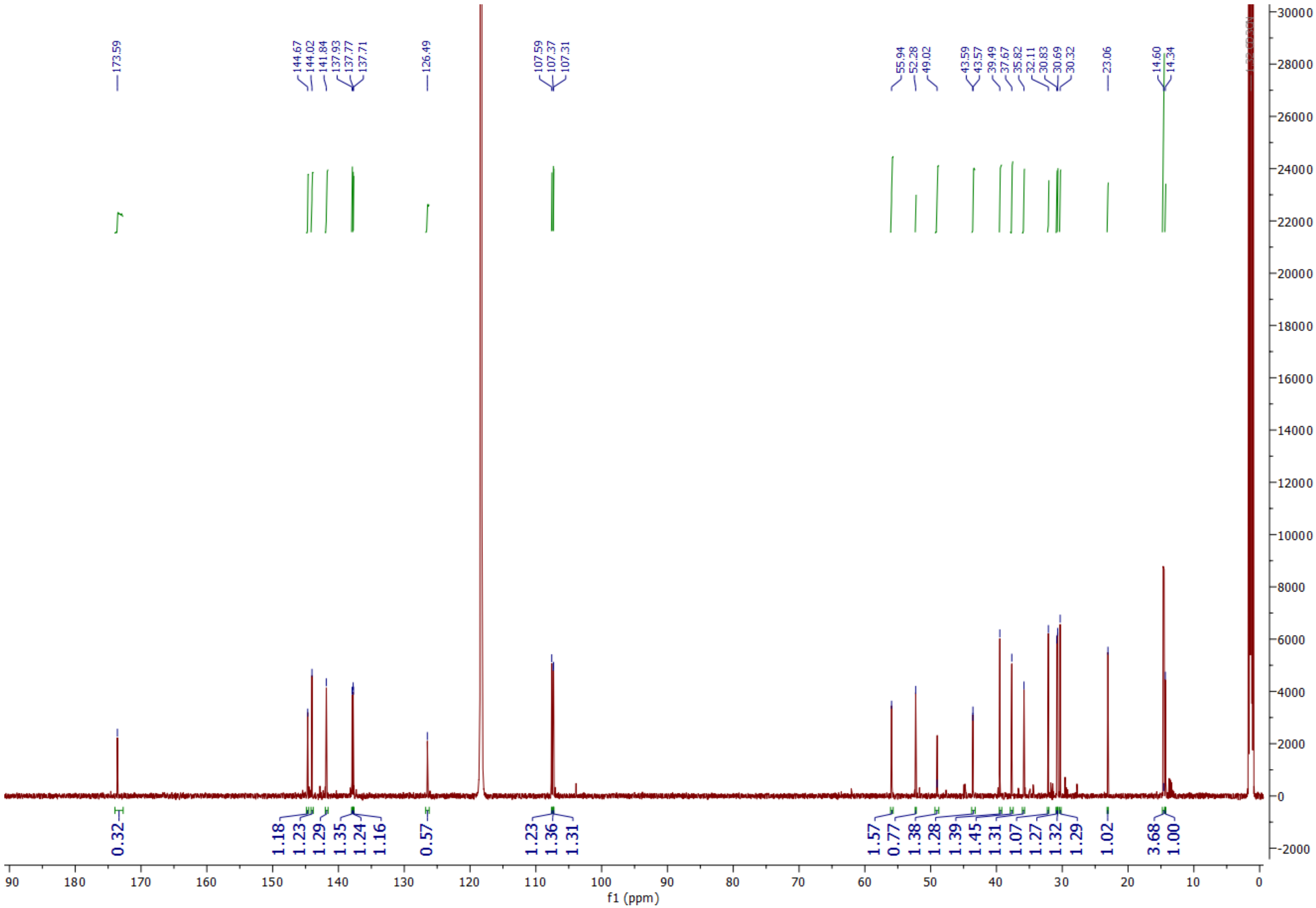

Supplementary Figure 20. ^{1}H NMR (CD_3Cl) of Compound **14**.

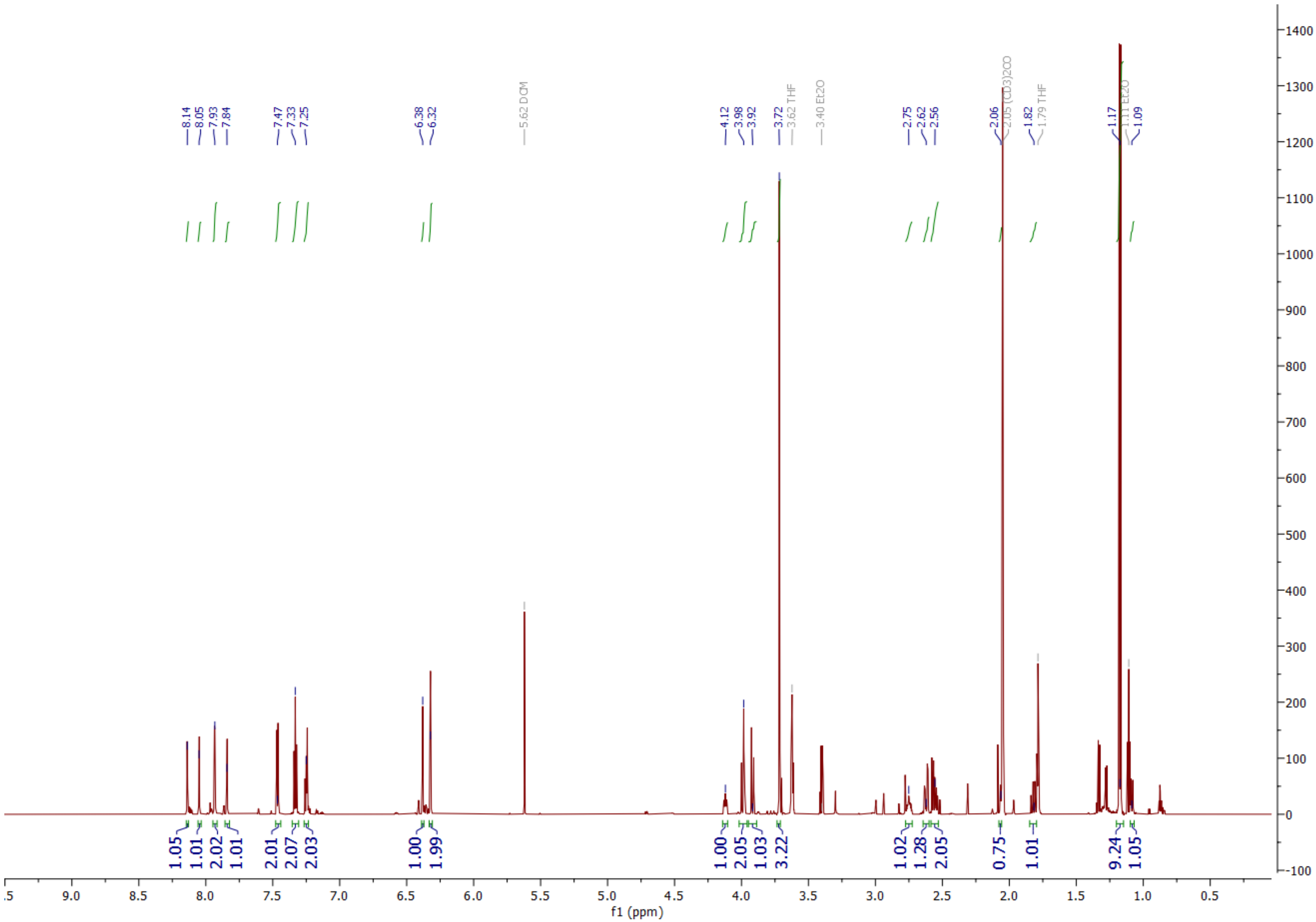

Supplementary Figure 21. ^{1}H NMR ($(CD_3)_2CO$) of Compound **15**.

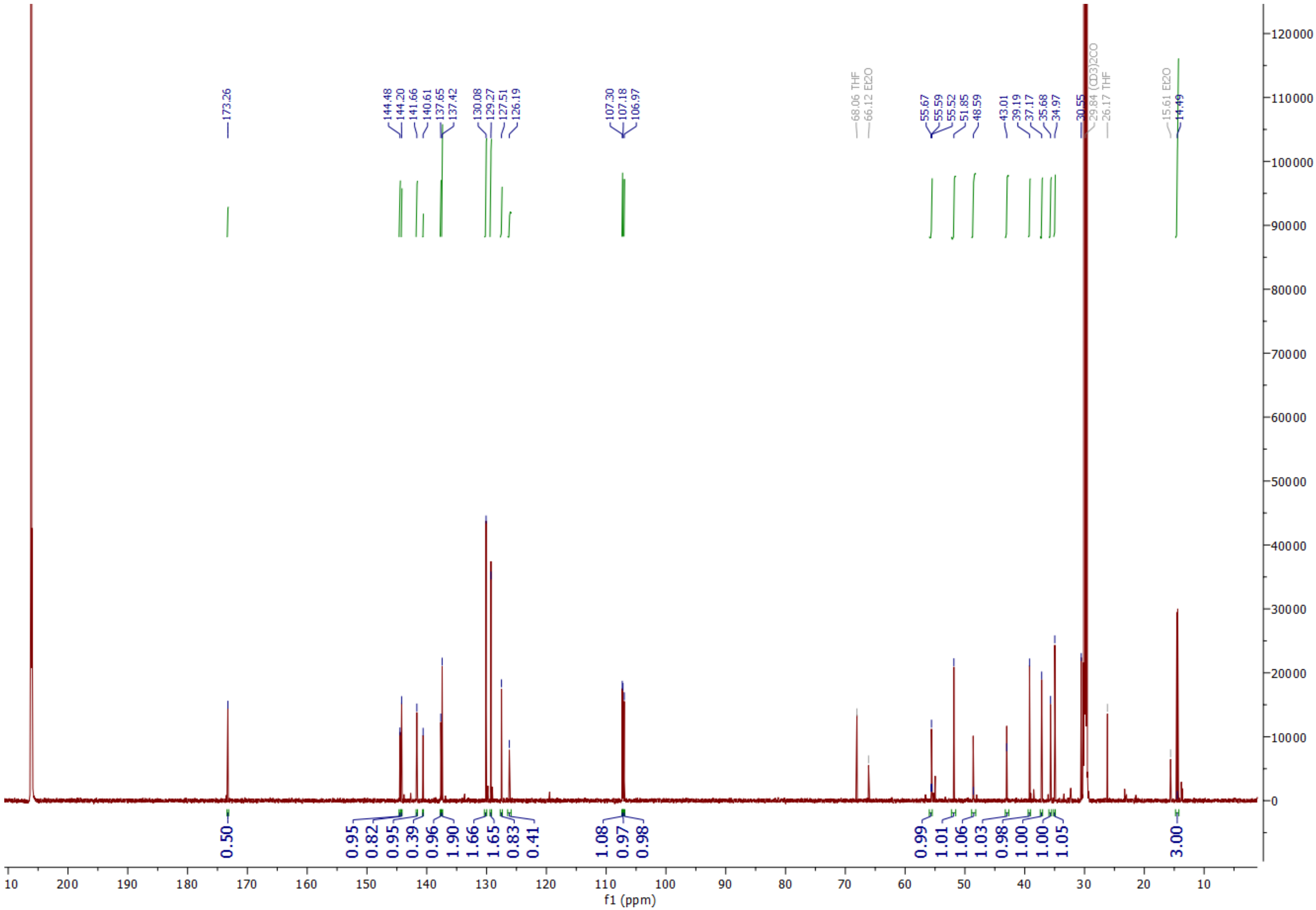

Supplementary Figure 22. ^{13}C NMR ($(CD_3)_2CO$) of Compound **15**.

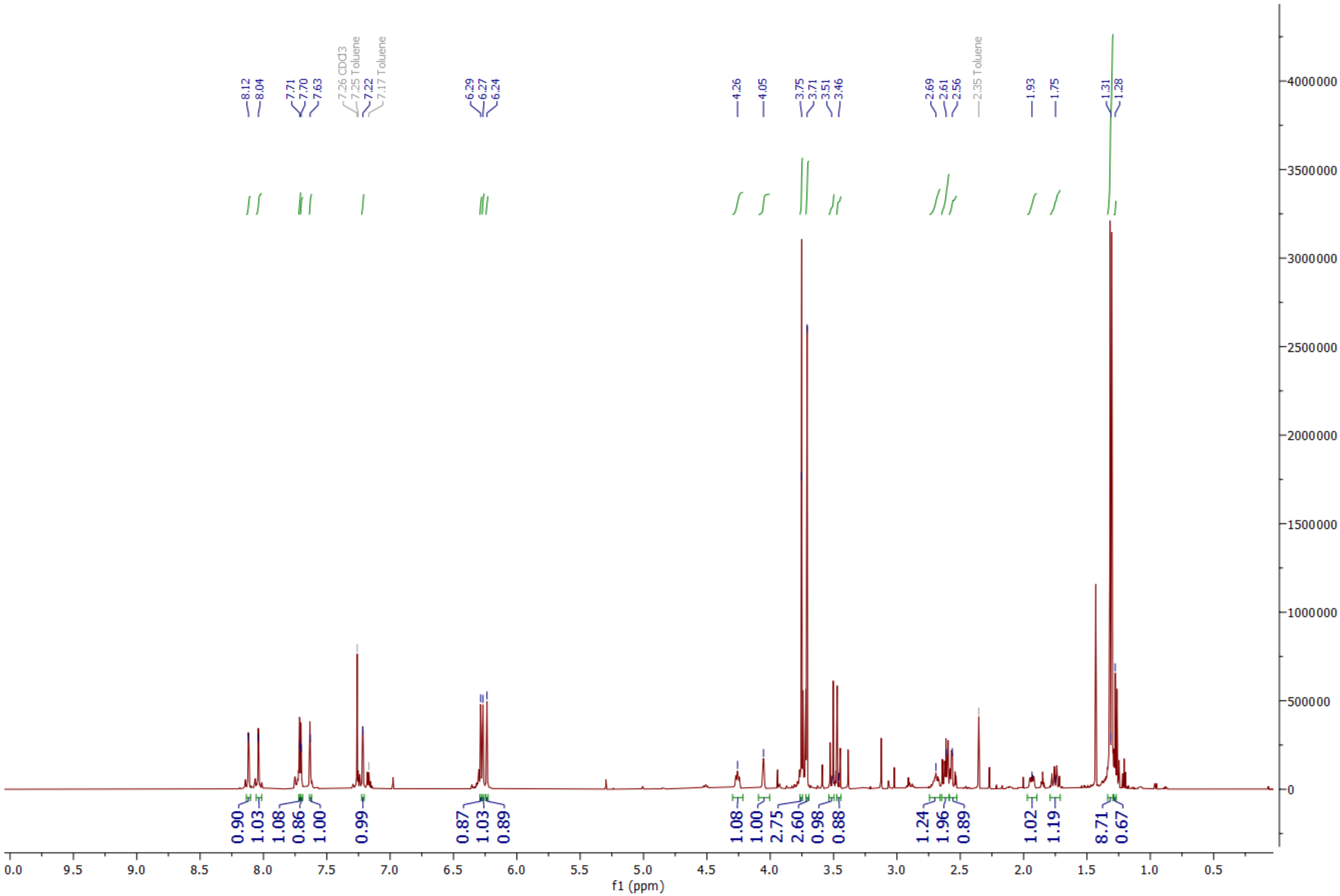

Supplementary Figure 23. ^{1}H NMR ($CDCL_3$) of Compound **16**.

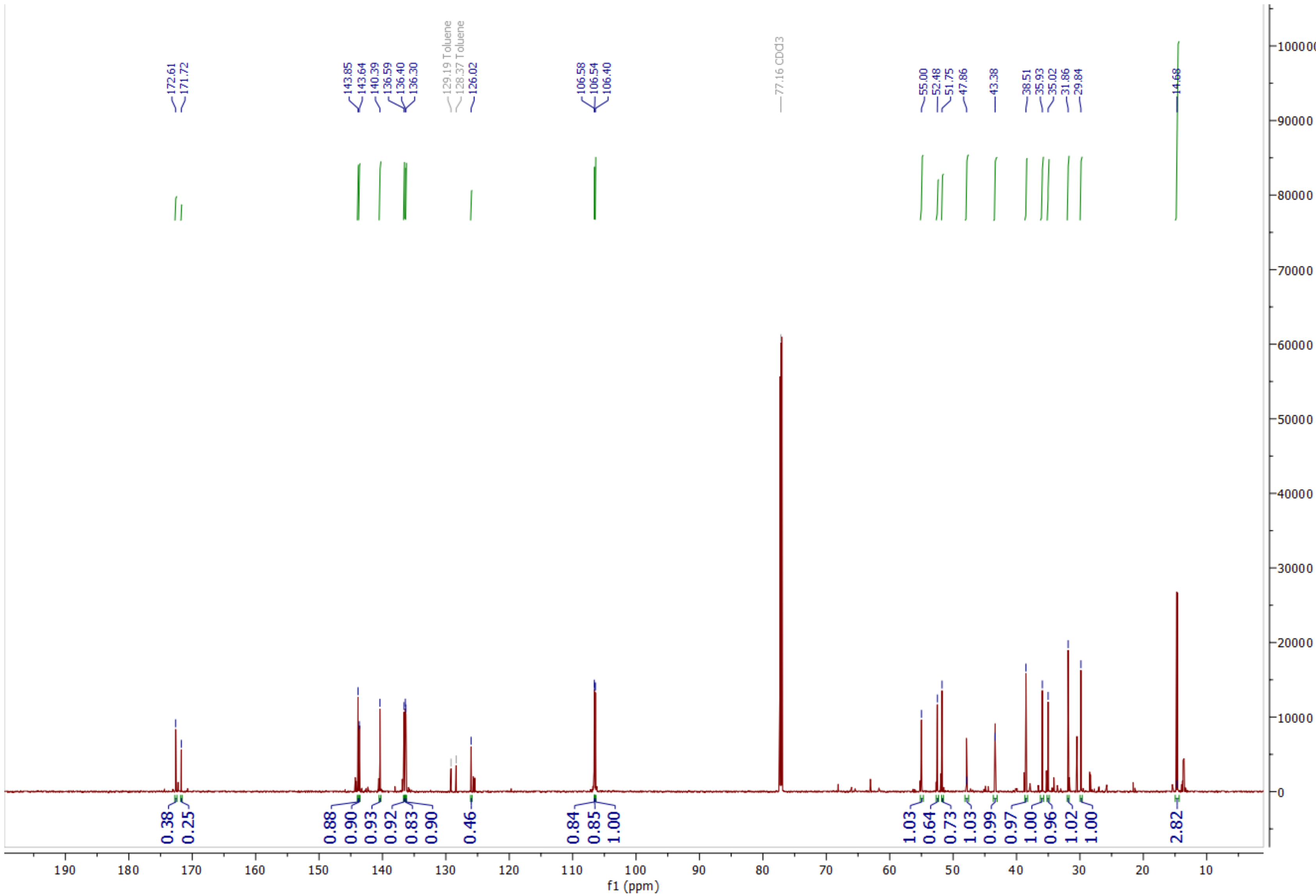

Supplementary Figure 24. ^{13}C NMR ($CDCL_3$) of Compound **16**.

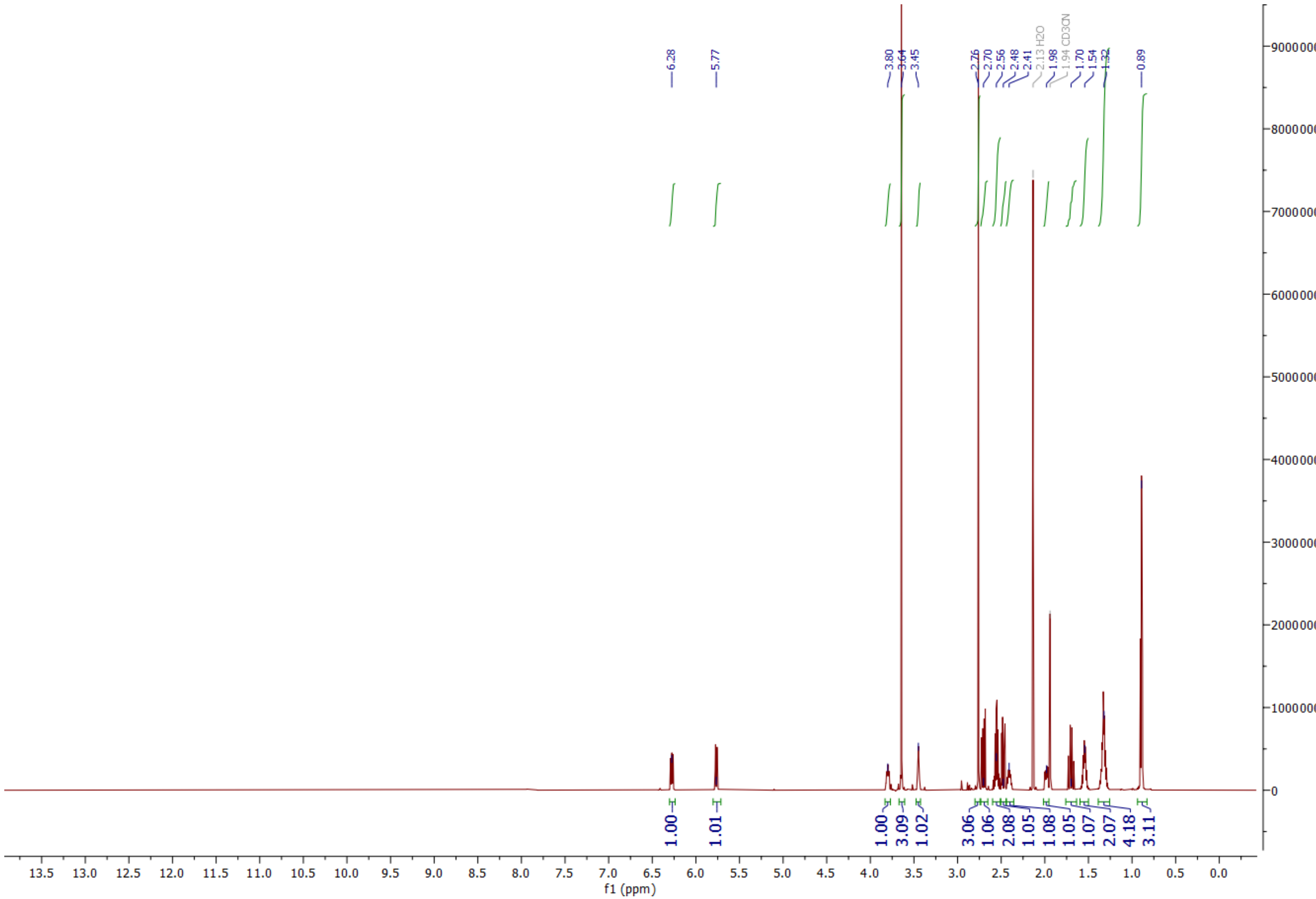

Supplementary Figure 25. ^{1}H NMR (CD_3CN) of Compound **17**.

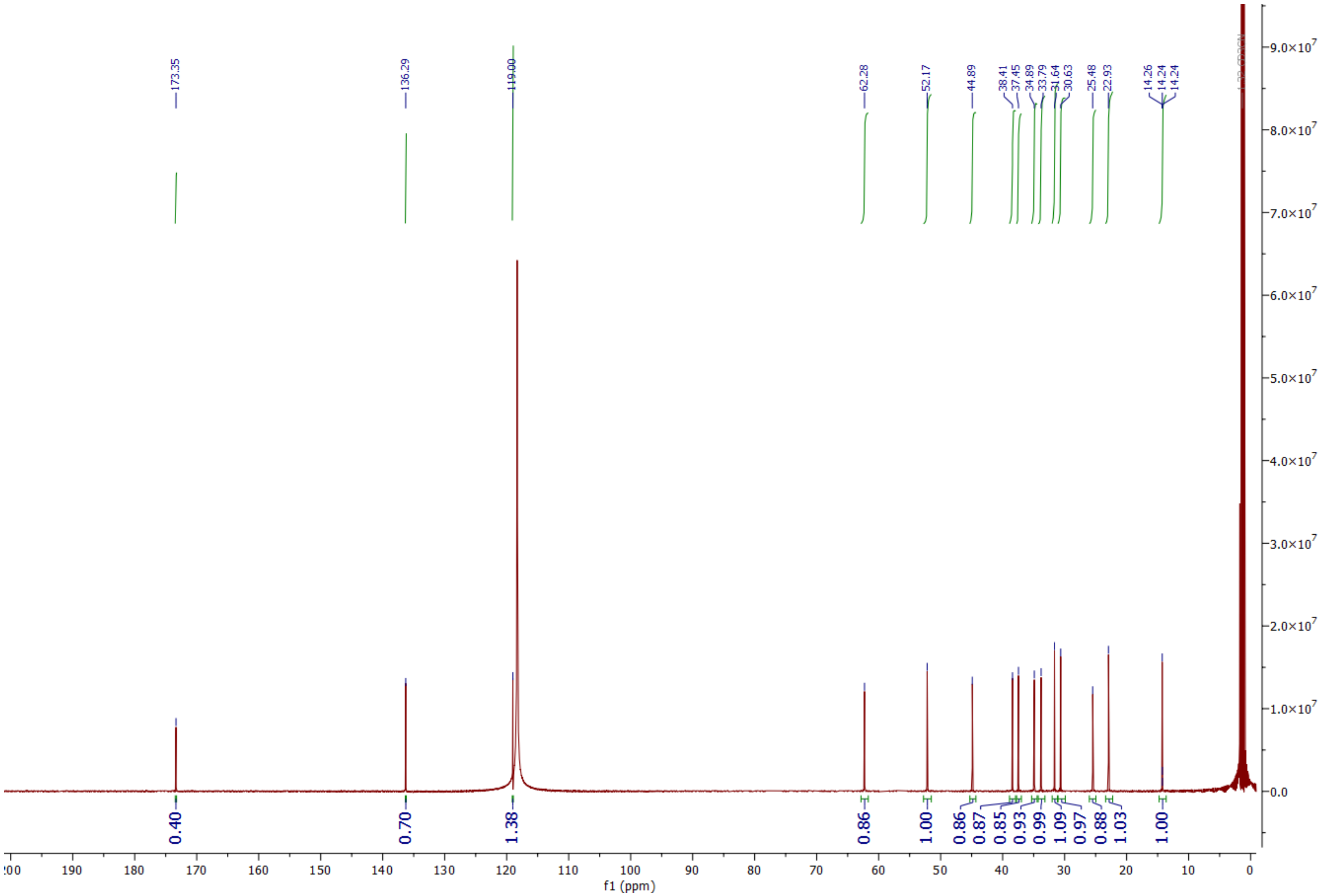

Supplementary Figure 26. ^{13}C NMR (CD_3CN) of Compound **17**.

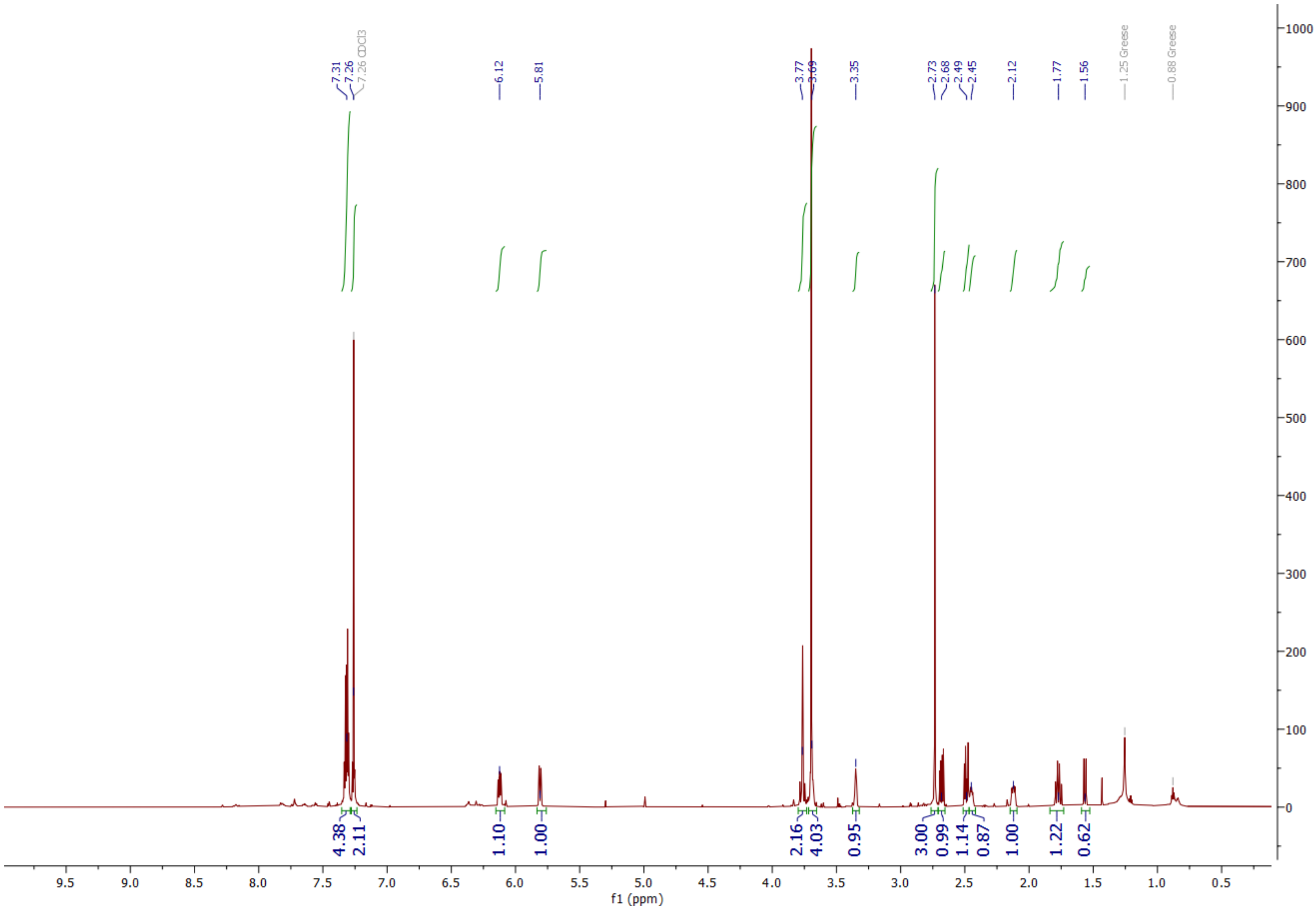

Supplementary Figure 27. ^{1}H NMR ($CDCl_3$) of Compound **18**.

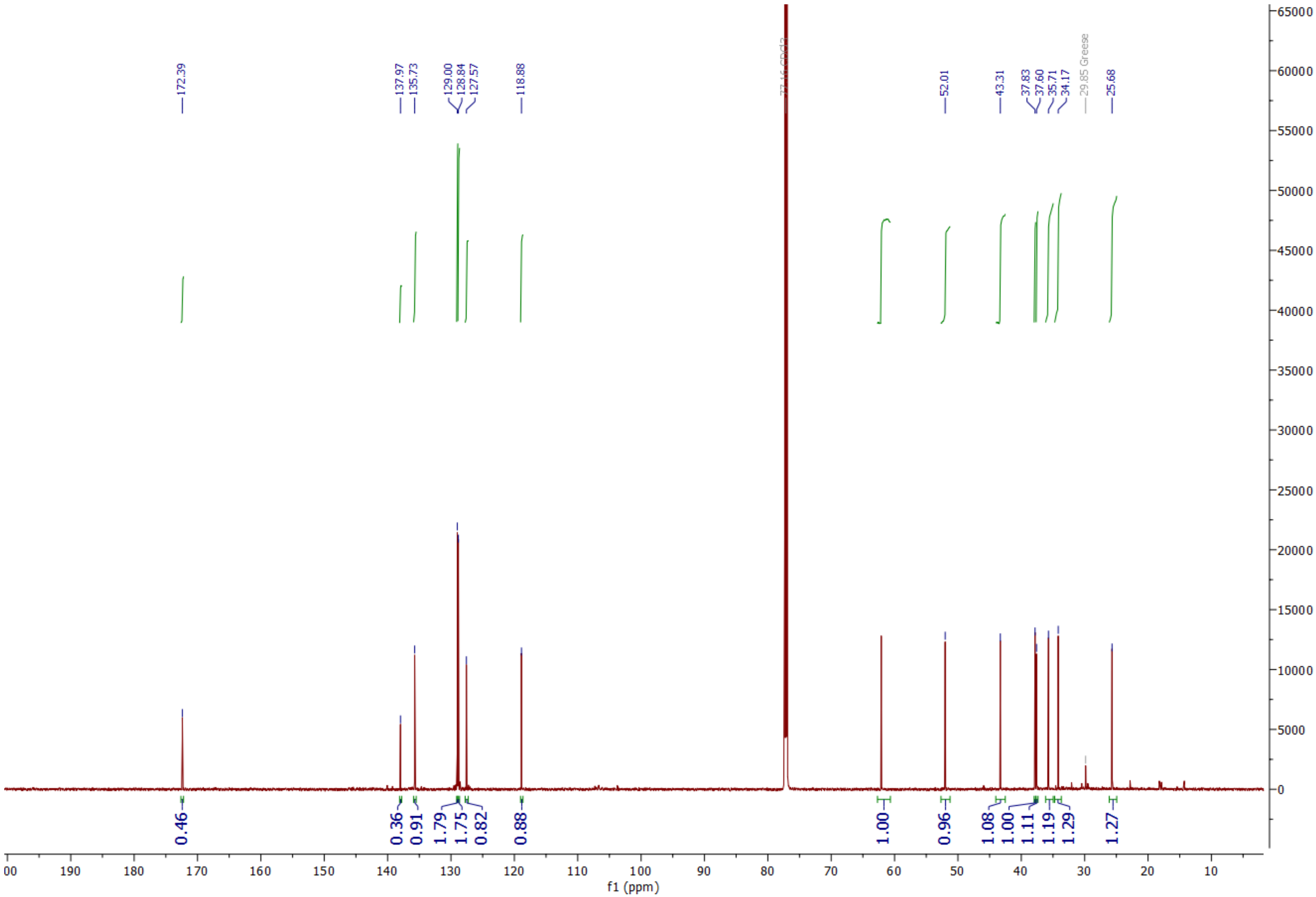

Supplementary Figure 28. ^{13}C NMR ($CDCl_3$) of Compound **18**.

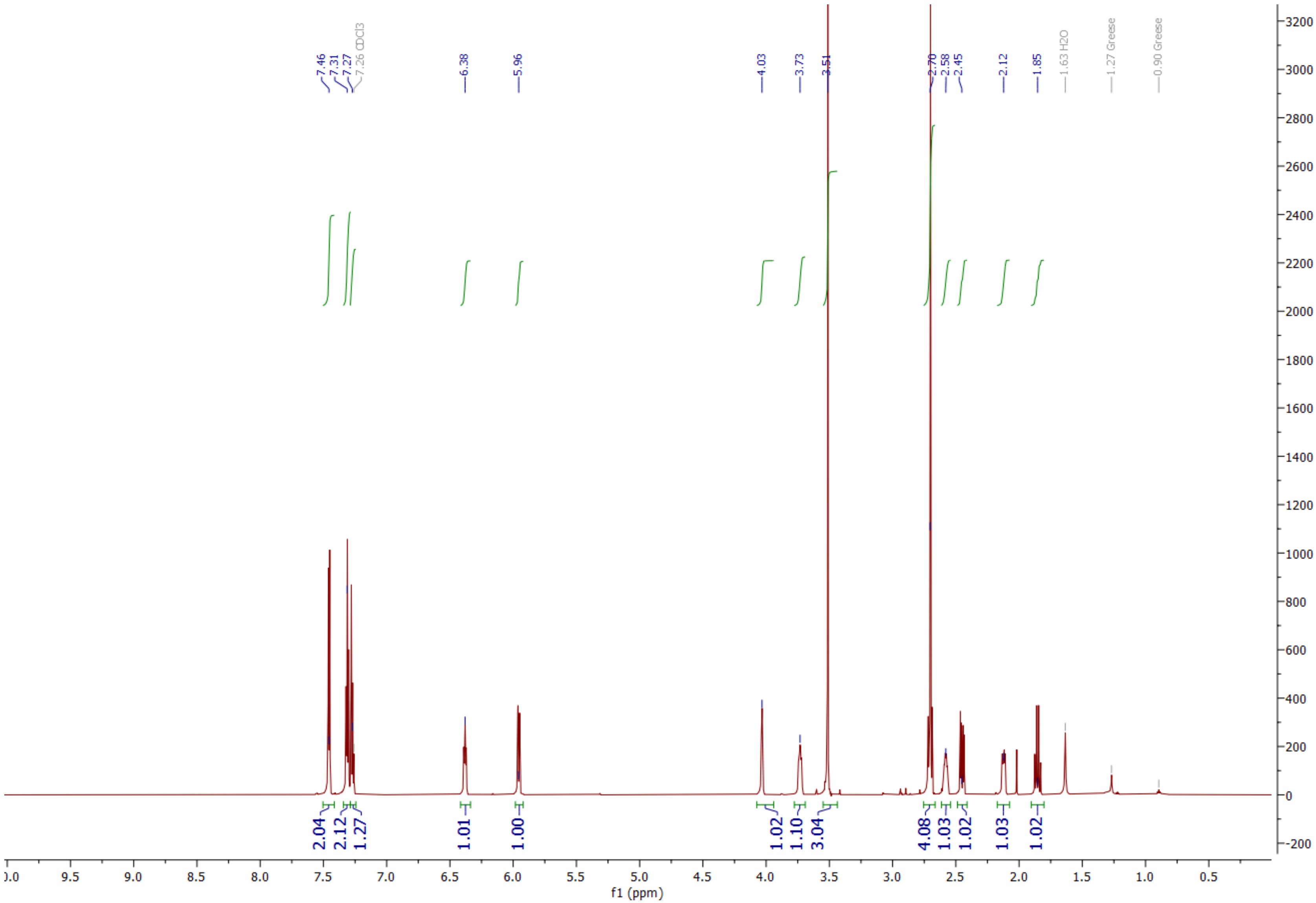

Supplementary Figure 29. ^{1}H NMR ($CDCl_3$) of Compound **19**.

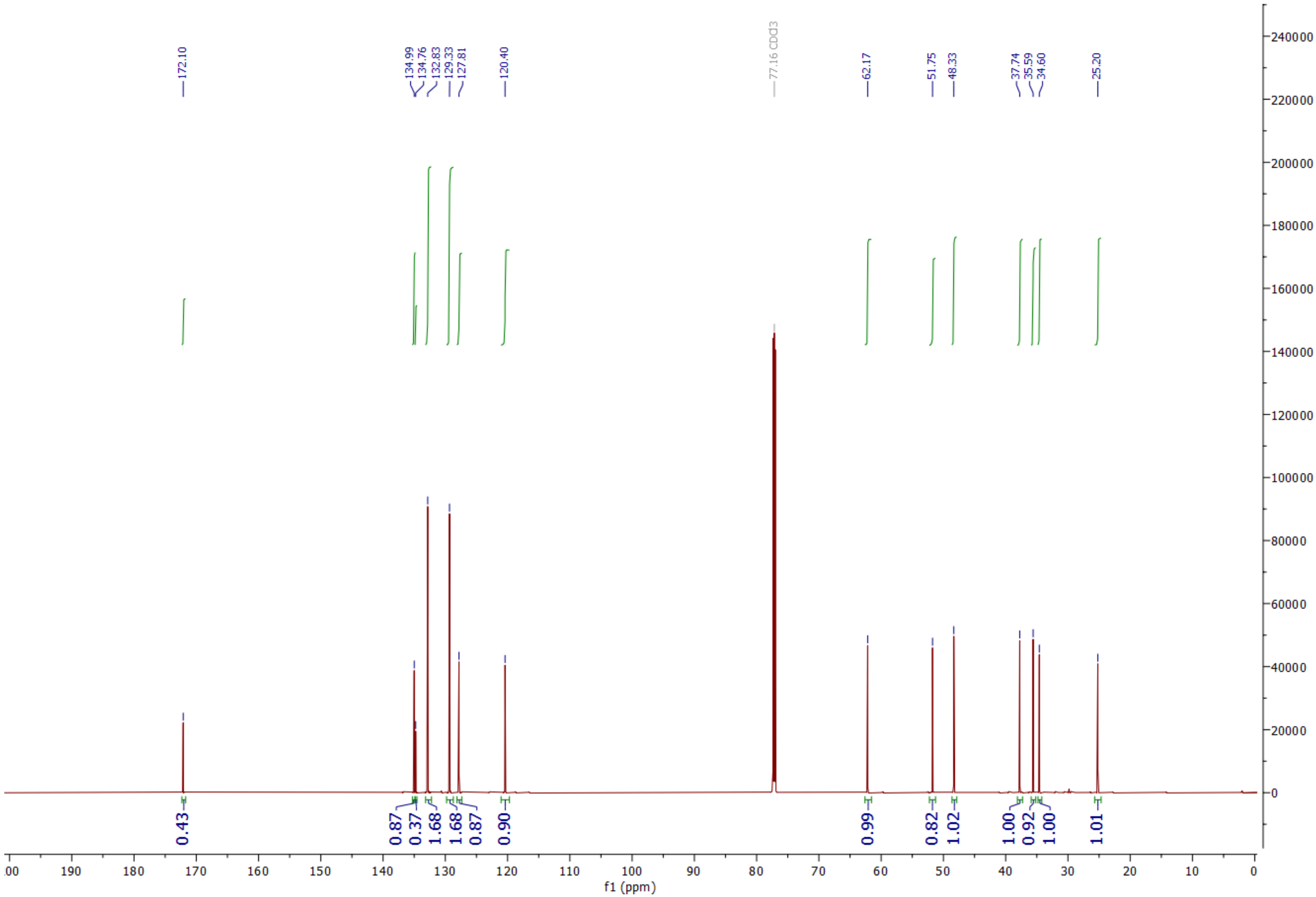

Supplementary Figure 30. ^{13}C NMR ($CDCl_3$) of Compound **19**.

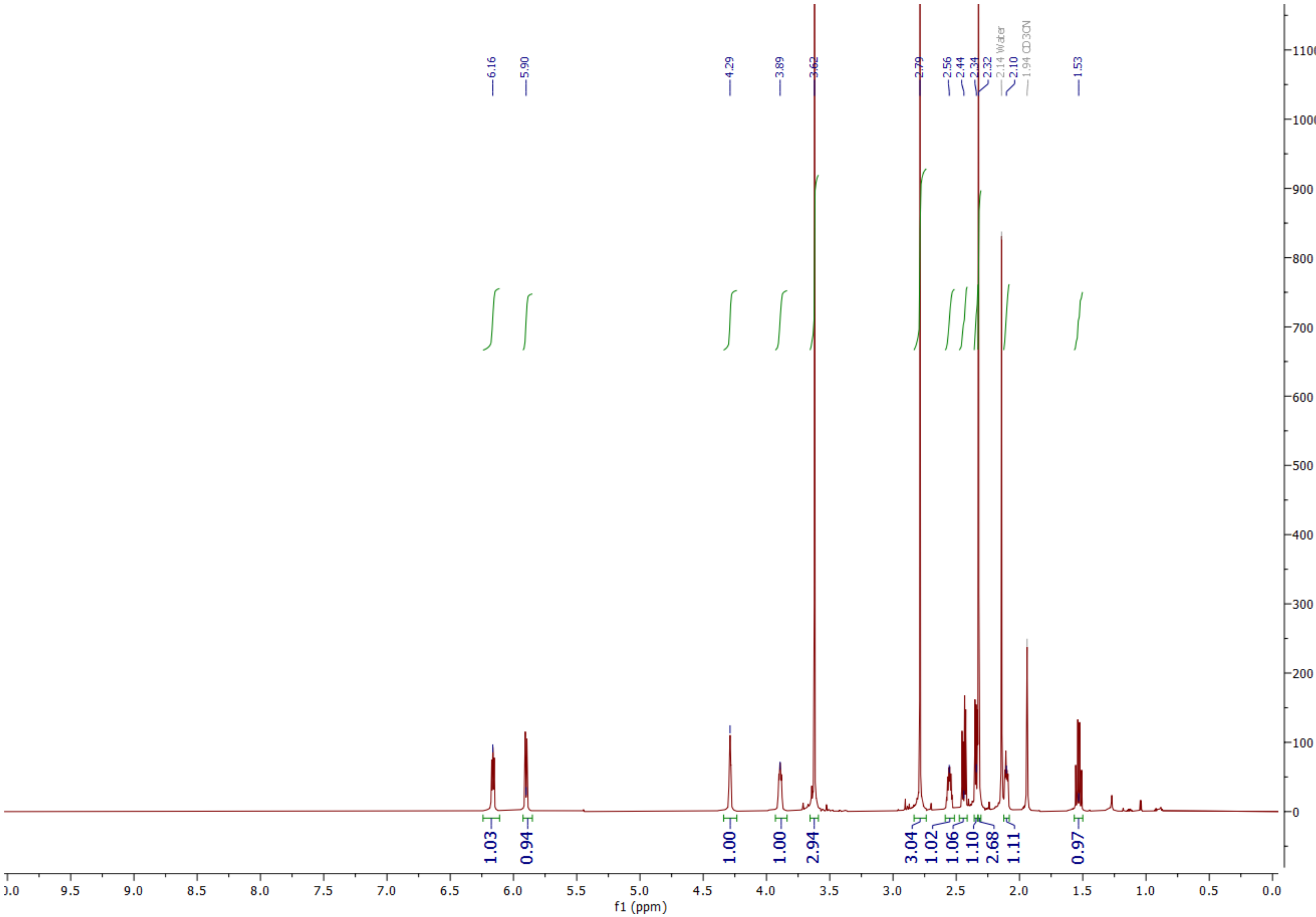

Supplementary Figure 31. ^{1}H NMR (CD_3CN) of Compound **20**.

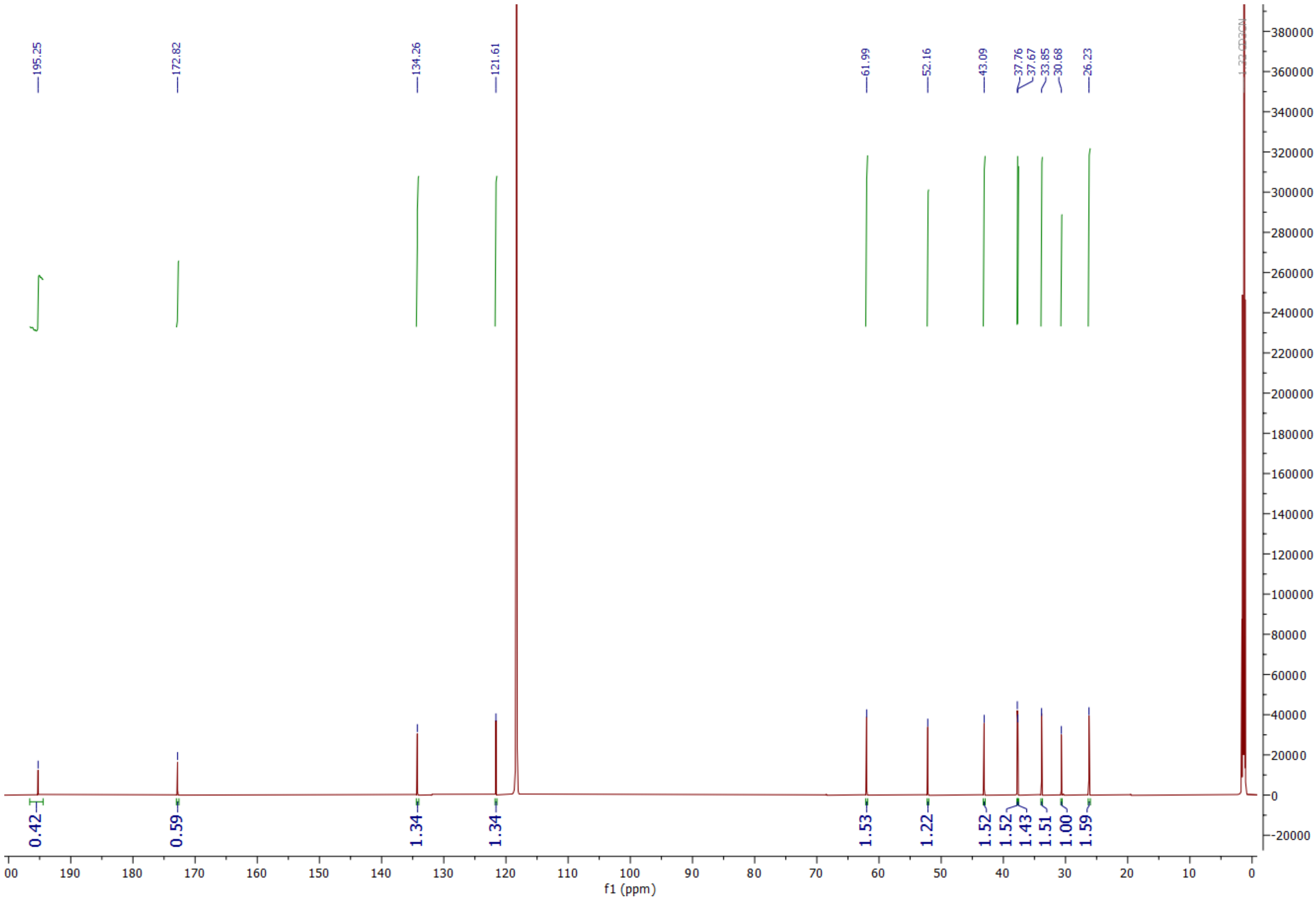

Supplementary Figure 32. ^{13}C NMR (CD_3CN) of Compound **20**.

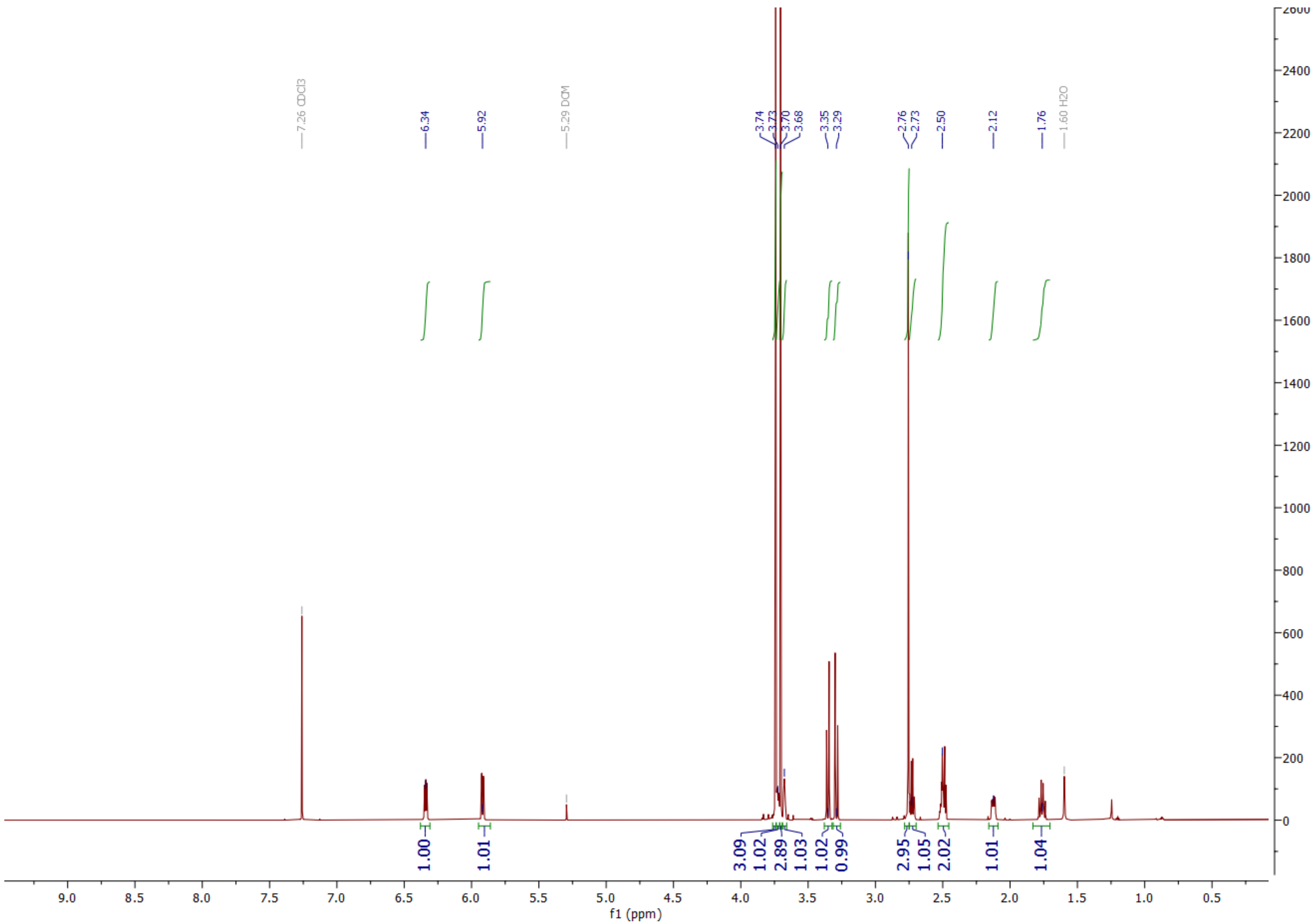

Supplementary Figure 33. ^{1}H NMR ($CDCl_3$) of Compound **21**.

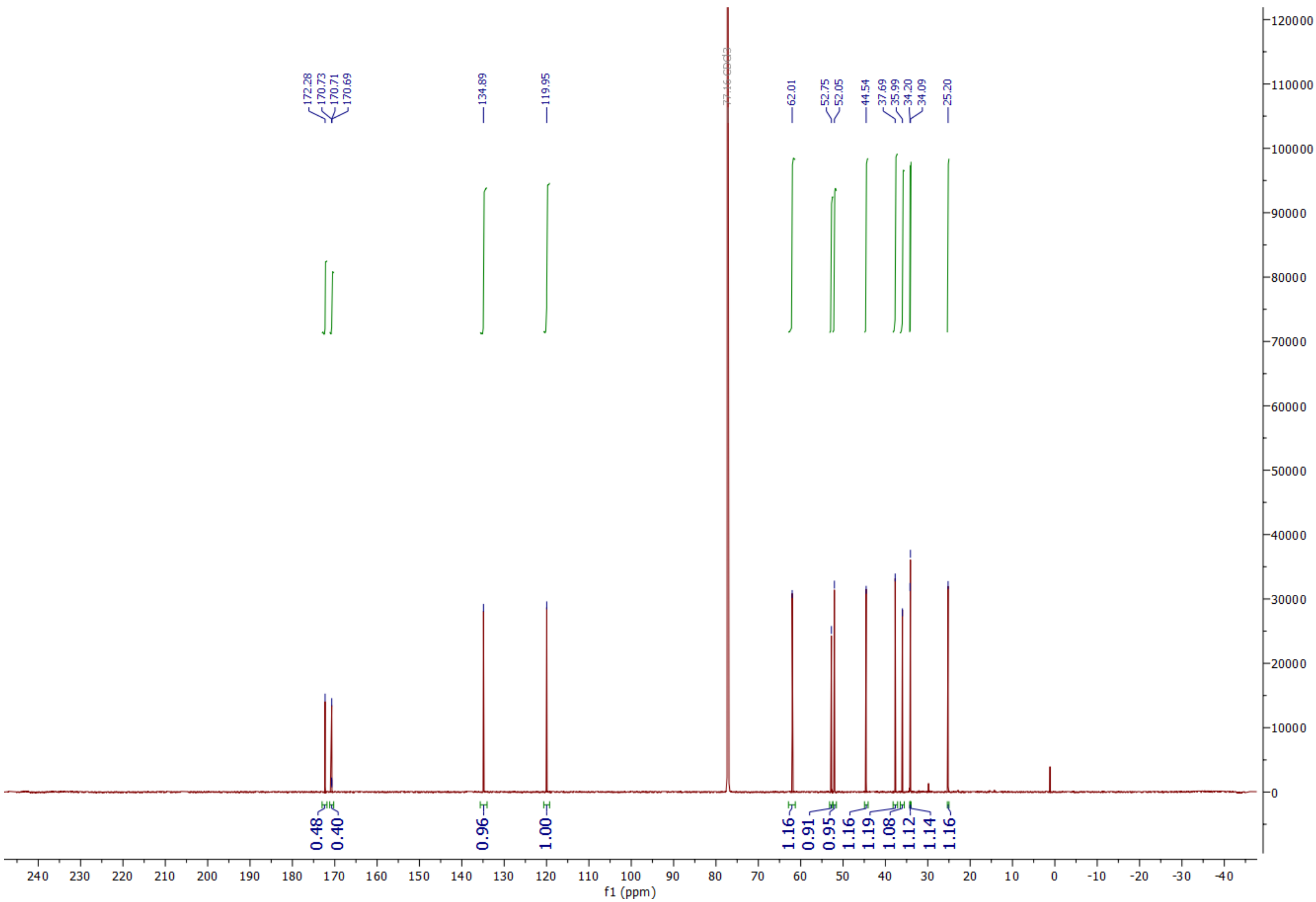

Supplementary Figure 34. ^{13}C NMR ($CDCl_3$) of Compound **21**.

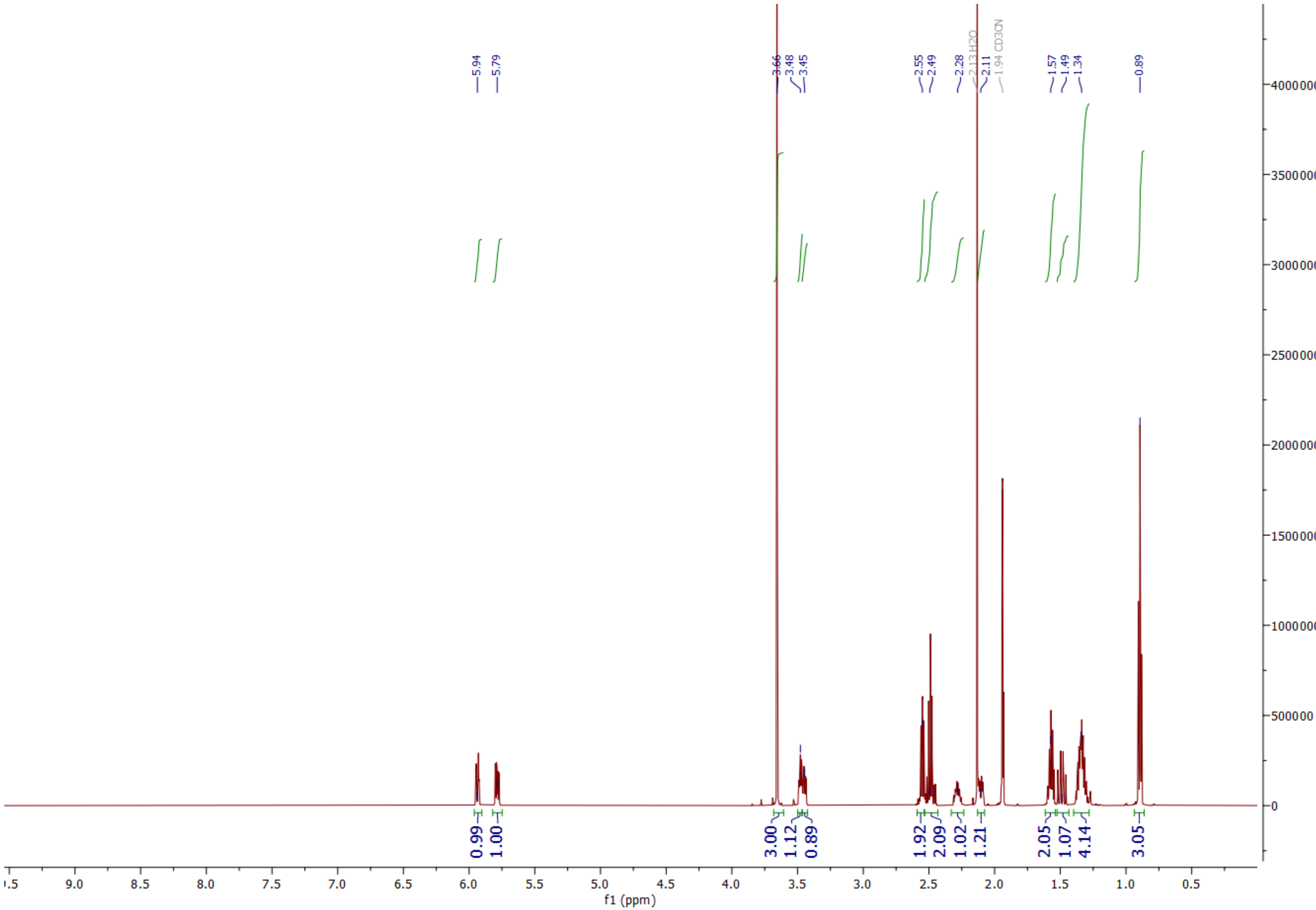

Supplementary Figure 35. ^{1}H NMR (CD_3CN) of Compound **22**.

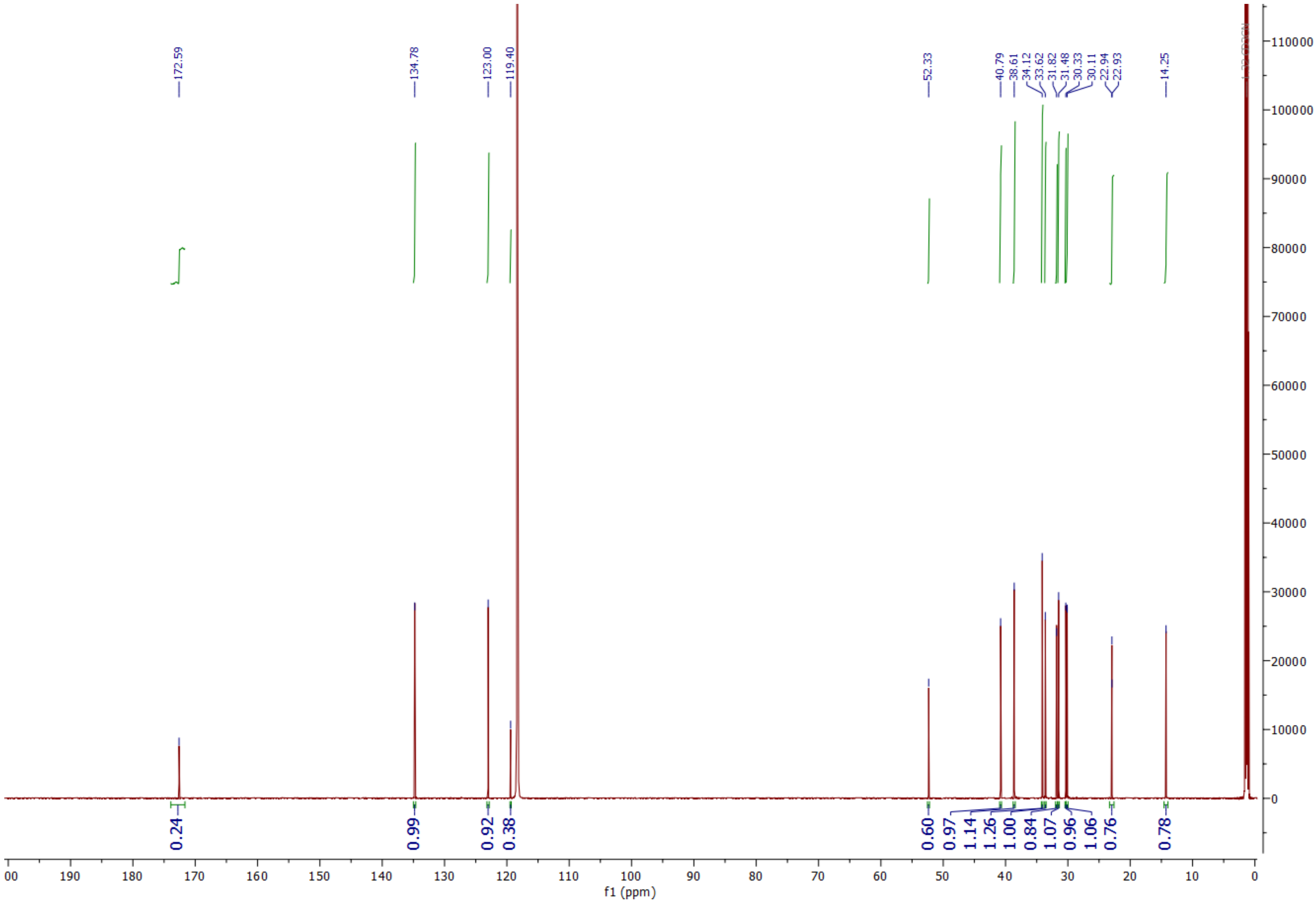

Supplementary Figure 36. ^{13}C NMR (CD_3CN) of Compound **22**.

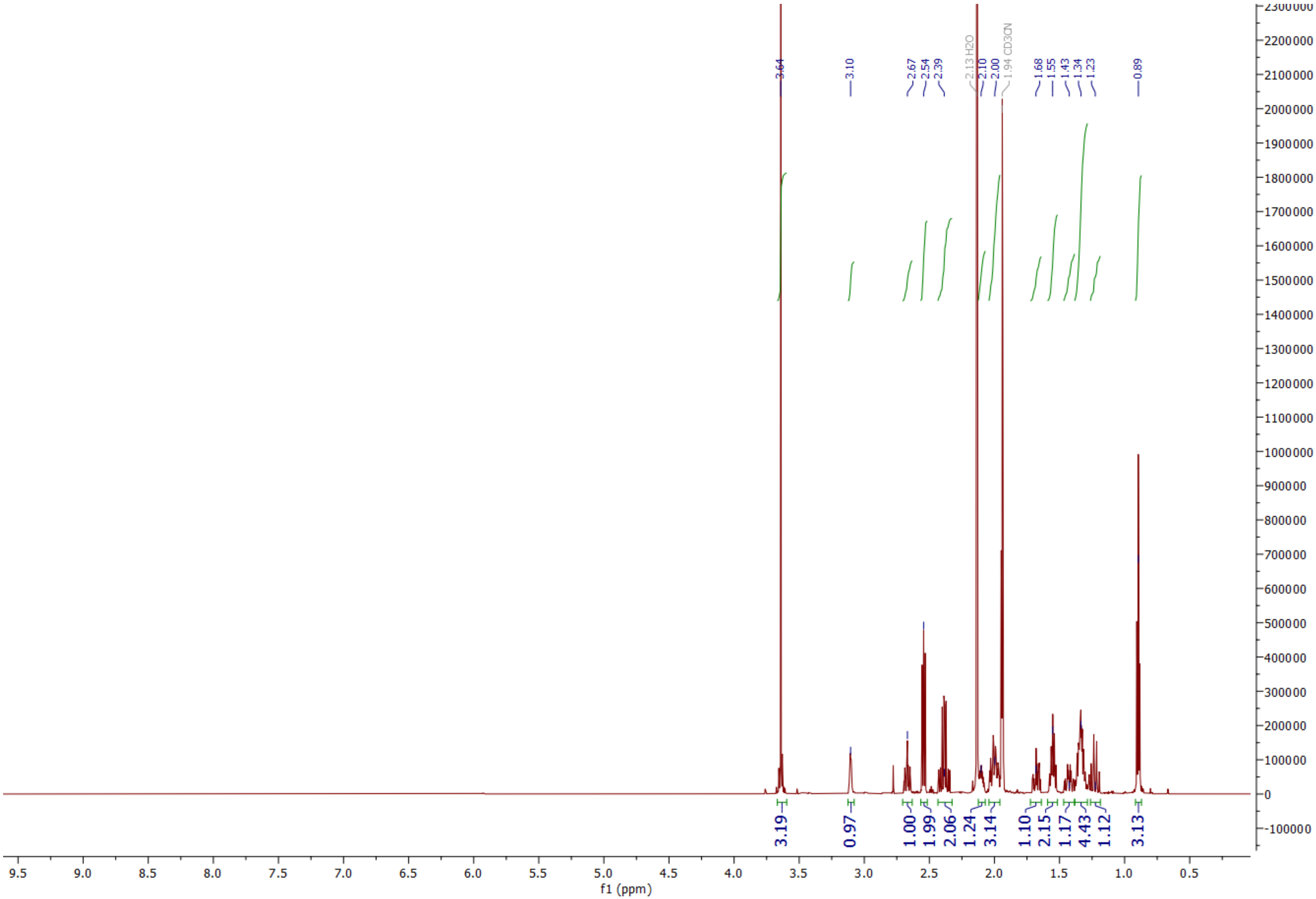

Supplementary Figure 37. ^{1}H NMR (CD_3CN) of Compound **23**.

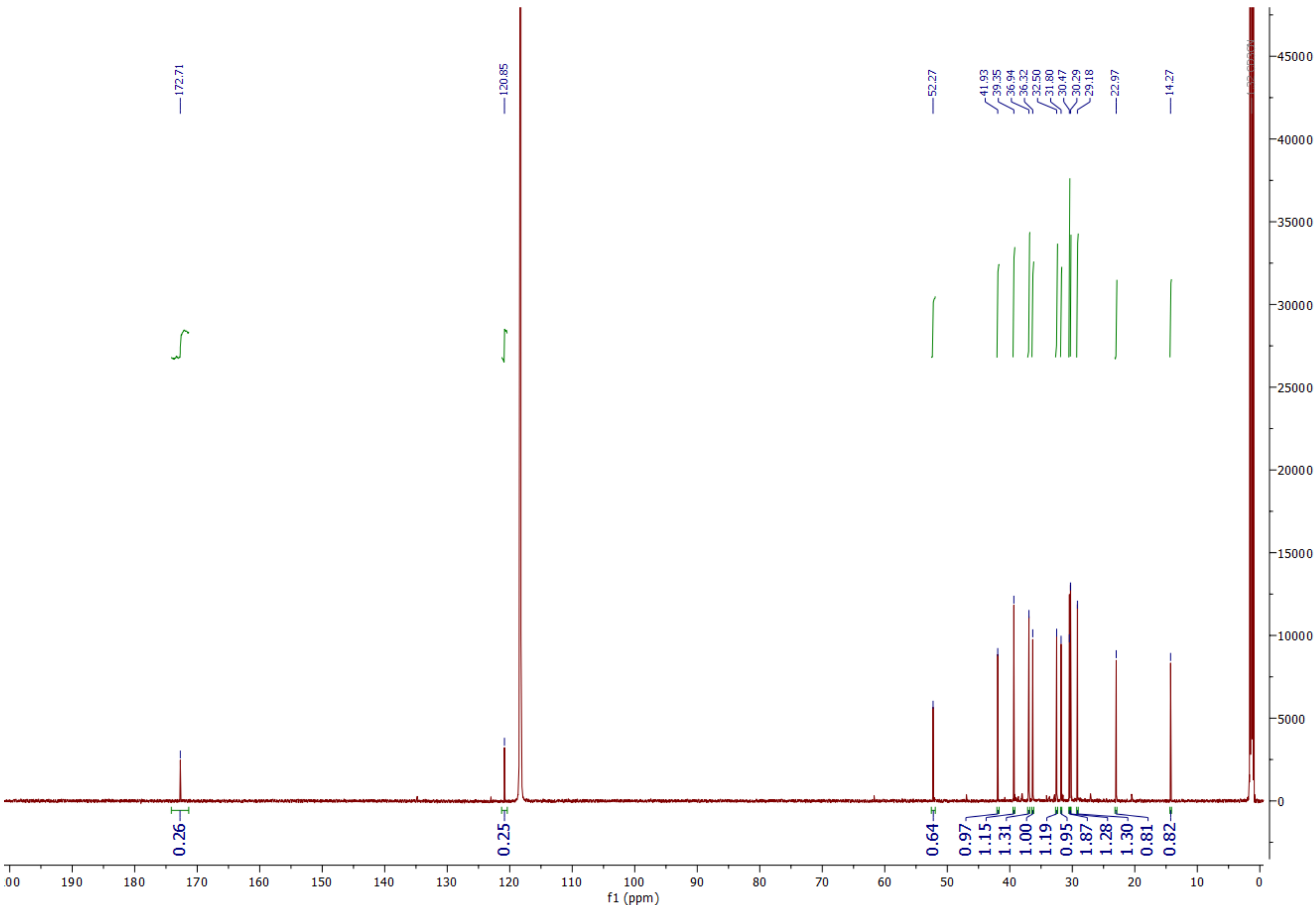

Supplementary Figure 38. ^{13}C NMR (CD_3CN) of Compound **23**.

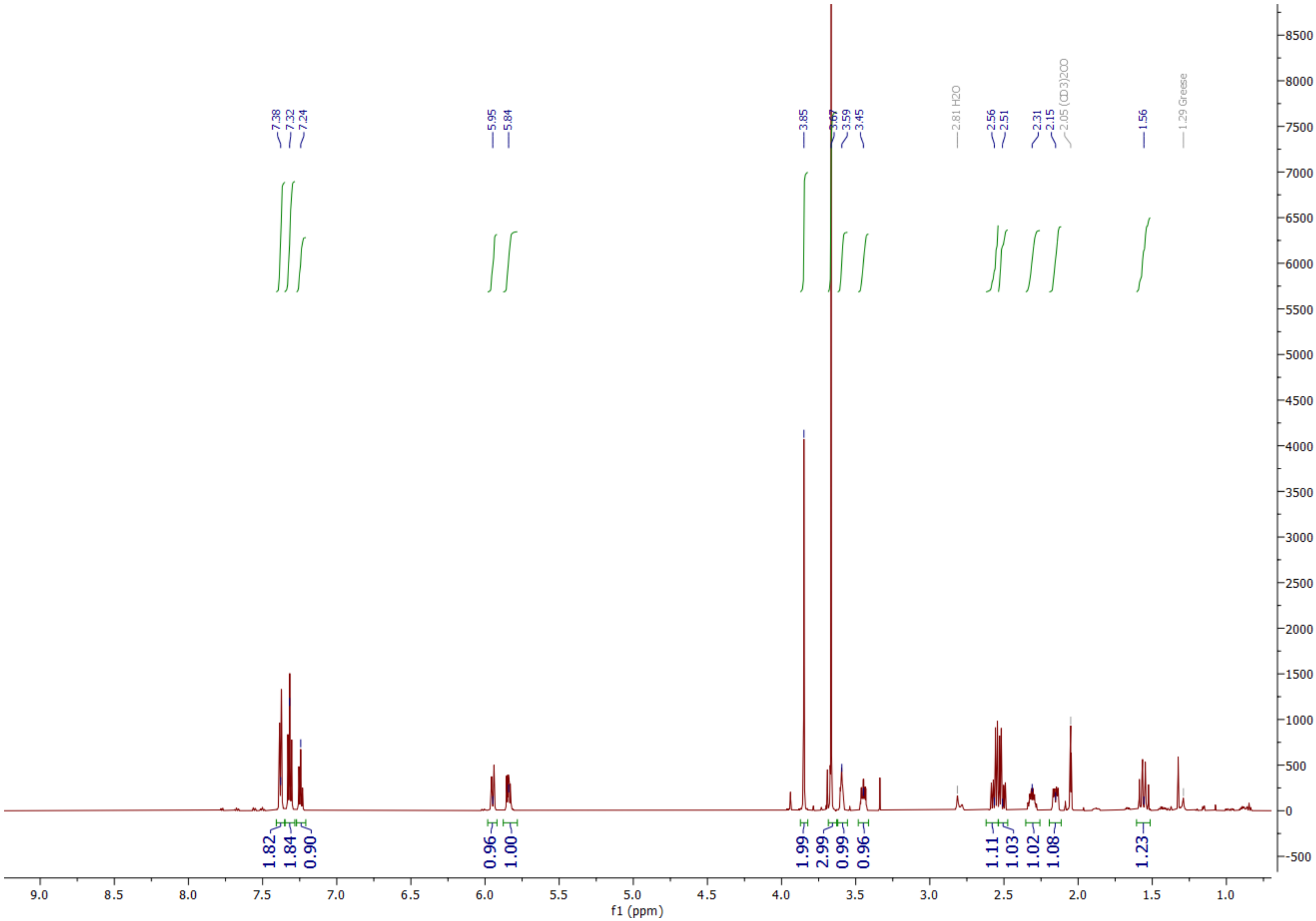

Supplementary Figure 39. ^{1}H NMR ($(CD_3)_2CO$) of Compound **24**.

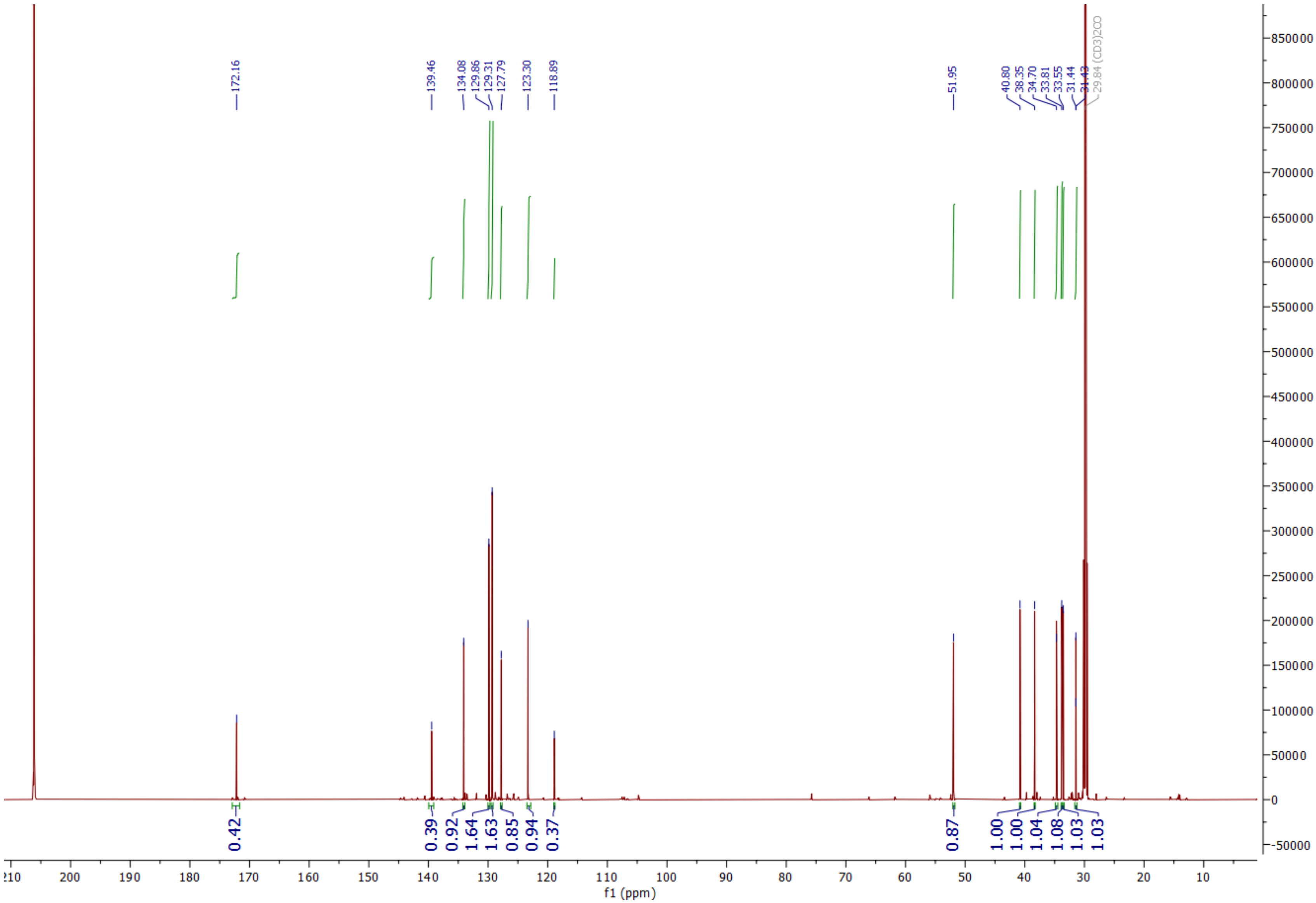

Supplementary Figure 40. ^{13}C NMR ($(CD_3)_2CO$) of Compound **24**.

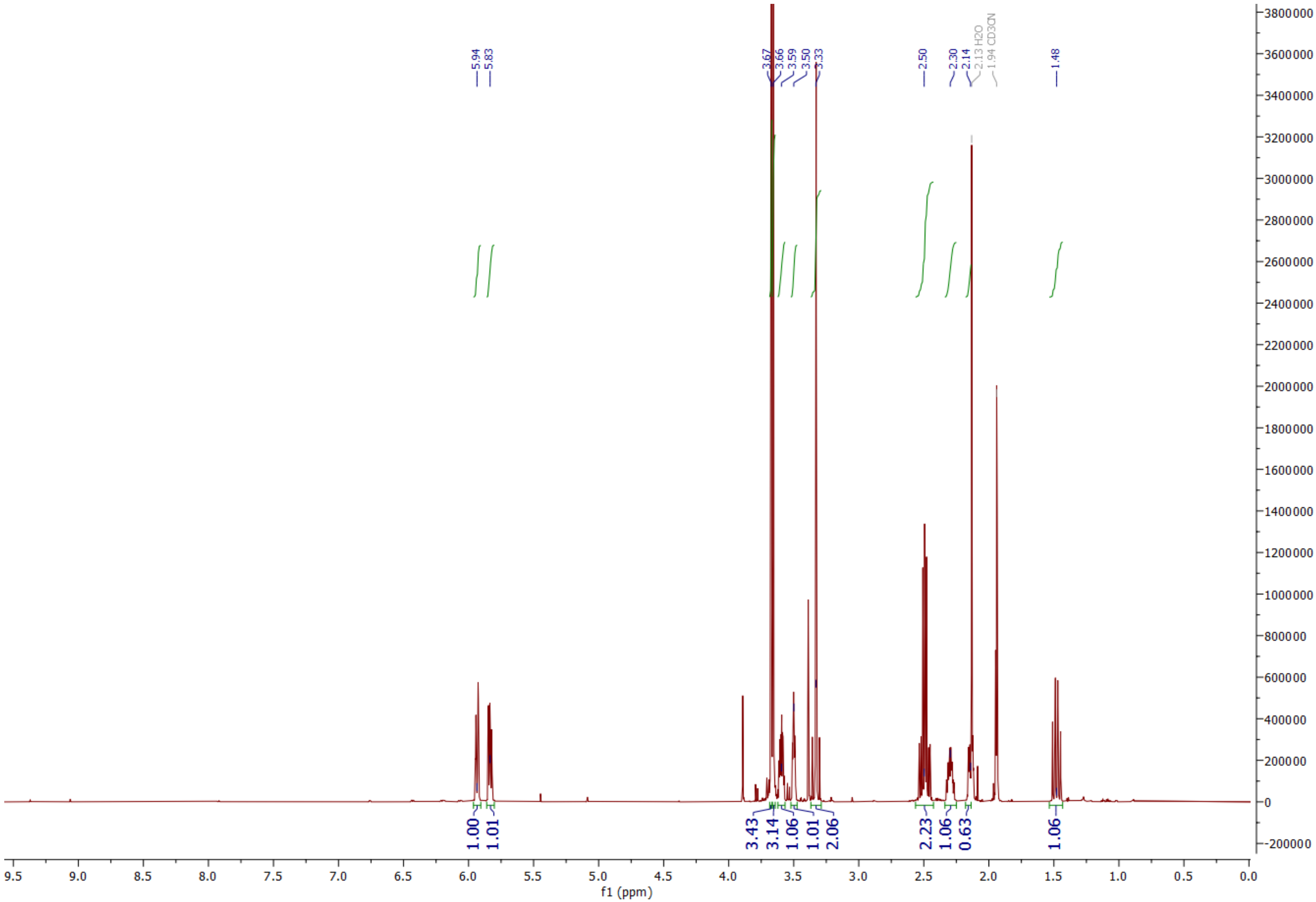

Supplementary Figure 41. ^{1}H NMR (CD_3CN) of Compound **25**.

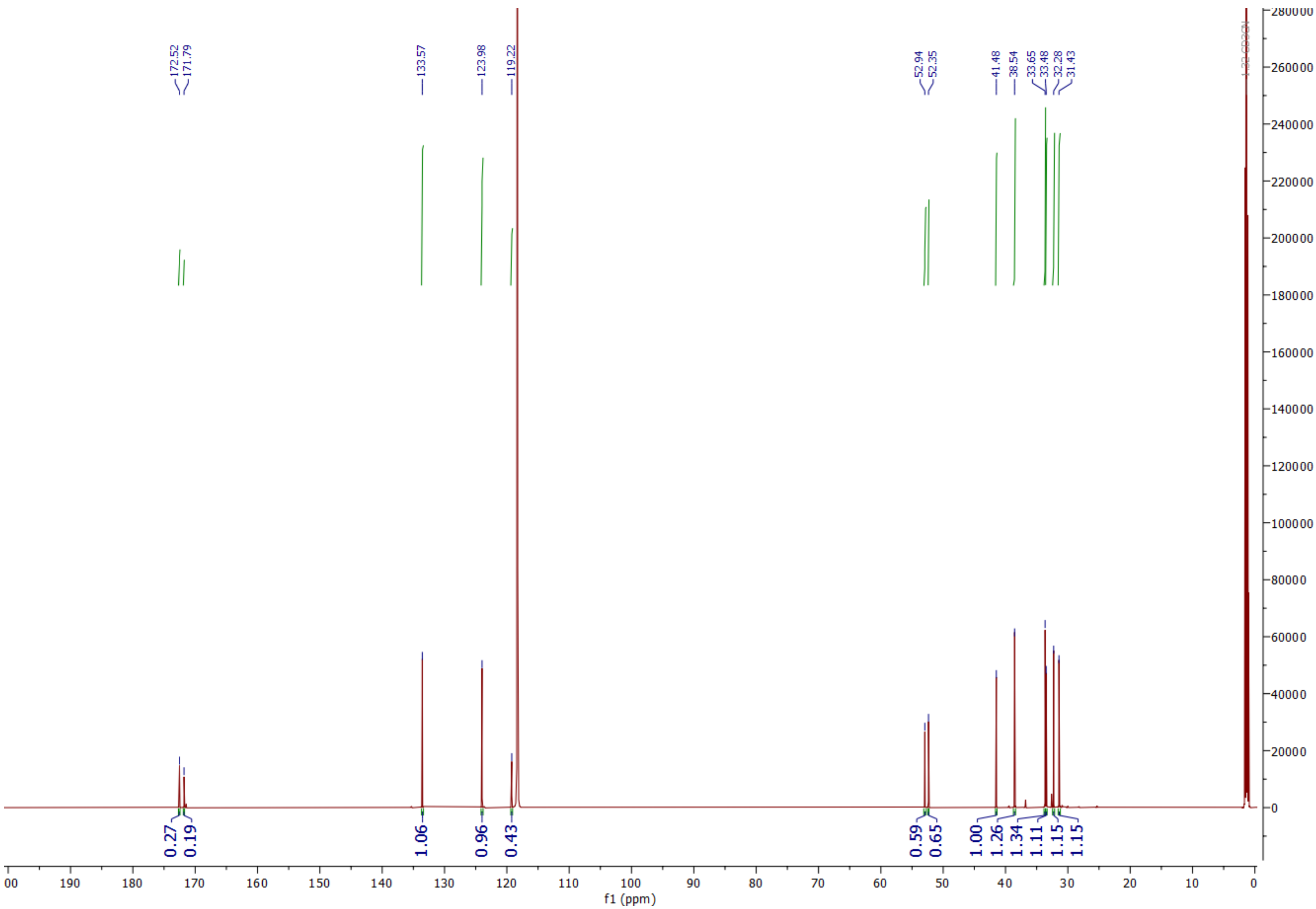

Supplementary Figure 42. ^{13}C NMR (CD_3CN) of Compound **25**.

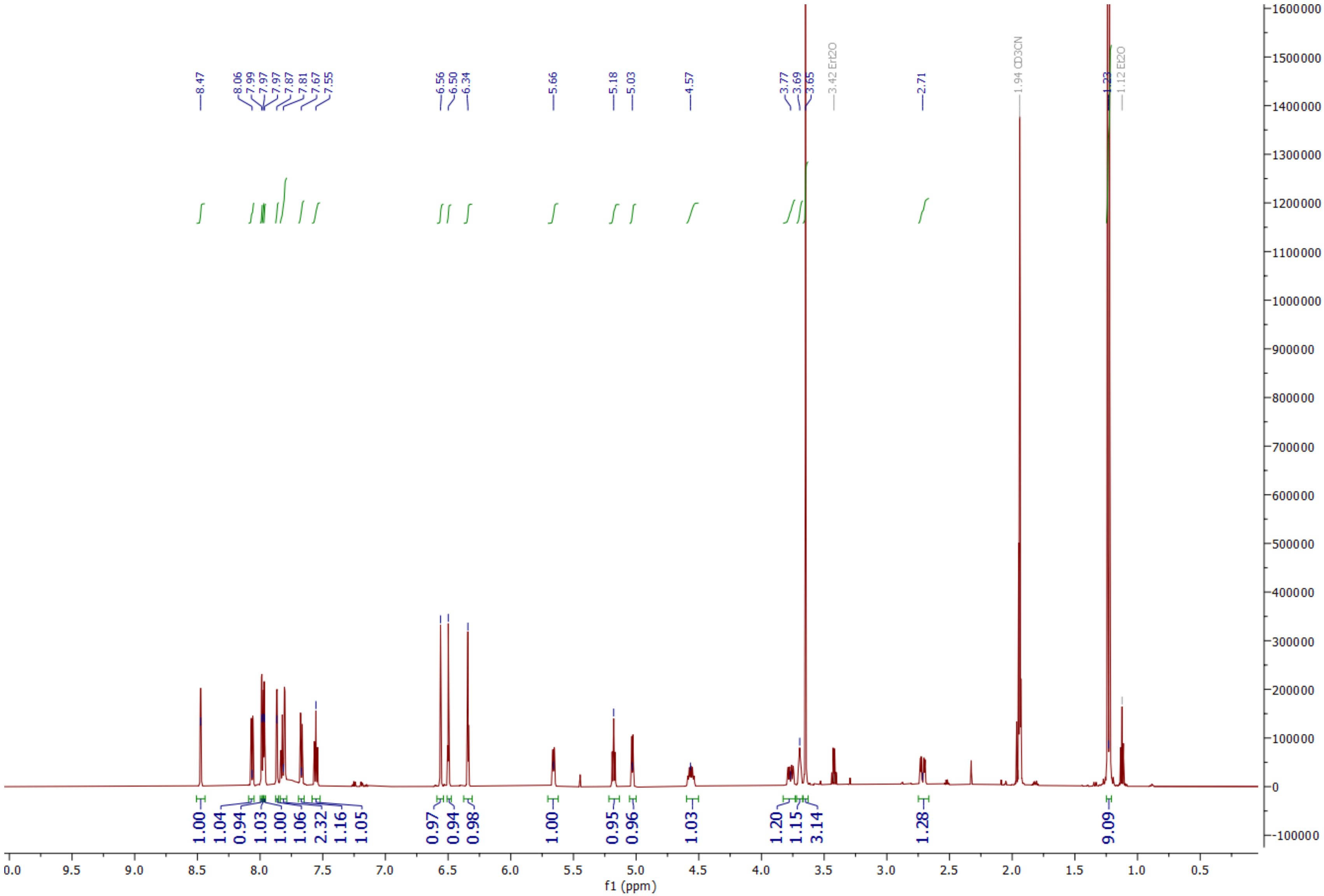

Supplementary Figure 43. ^{1}H NMR (CD_3CN) of Compound **28**.

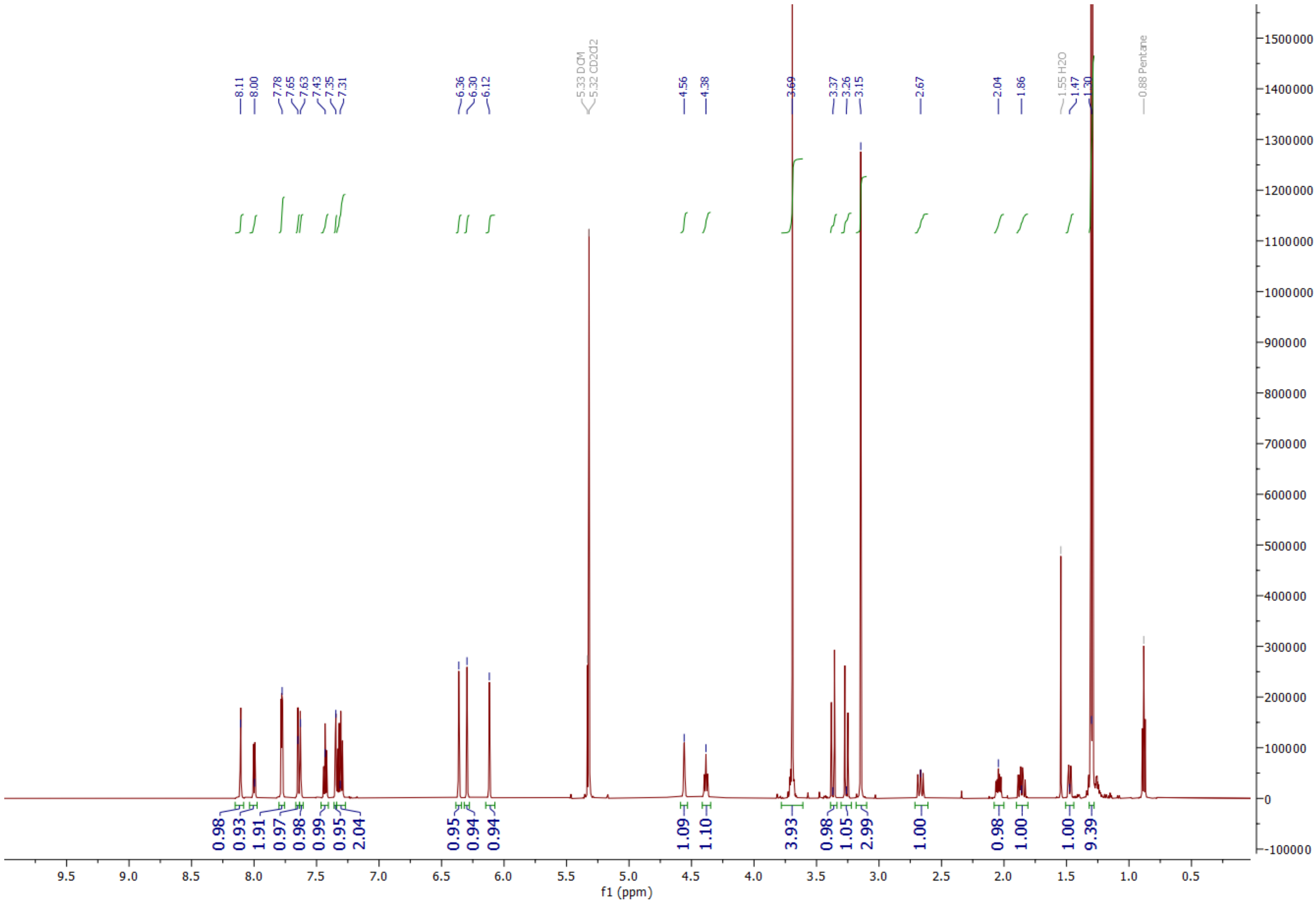

Supplementary Figure 44. ^{1}H NMR (CD_2Cl_2) of Compound **29**.

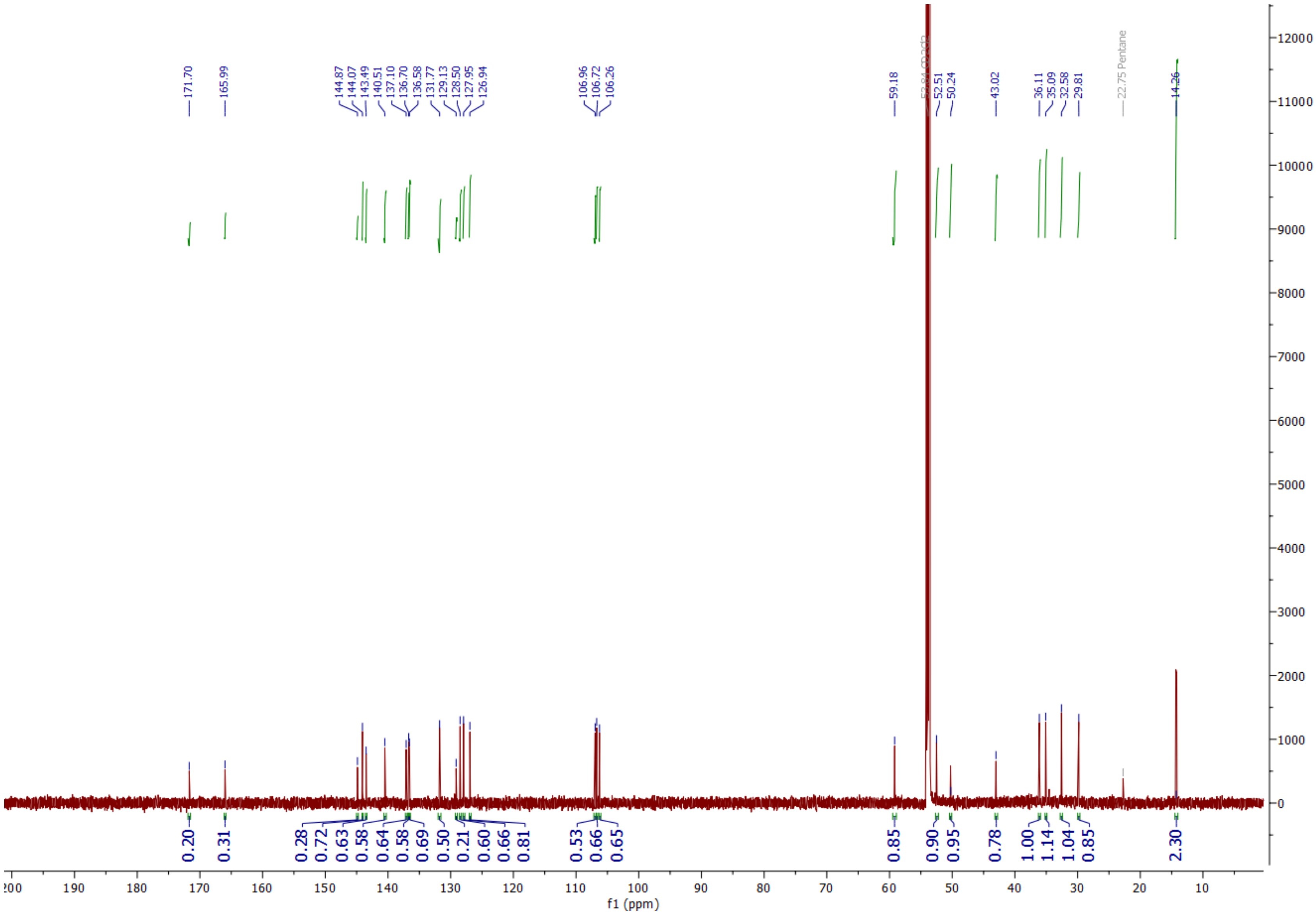

Supplementary Figure 45. ^{13}C NMR (CD_2Cl_2) of Compound **29**.

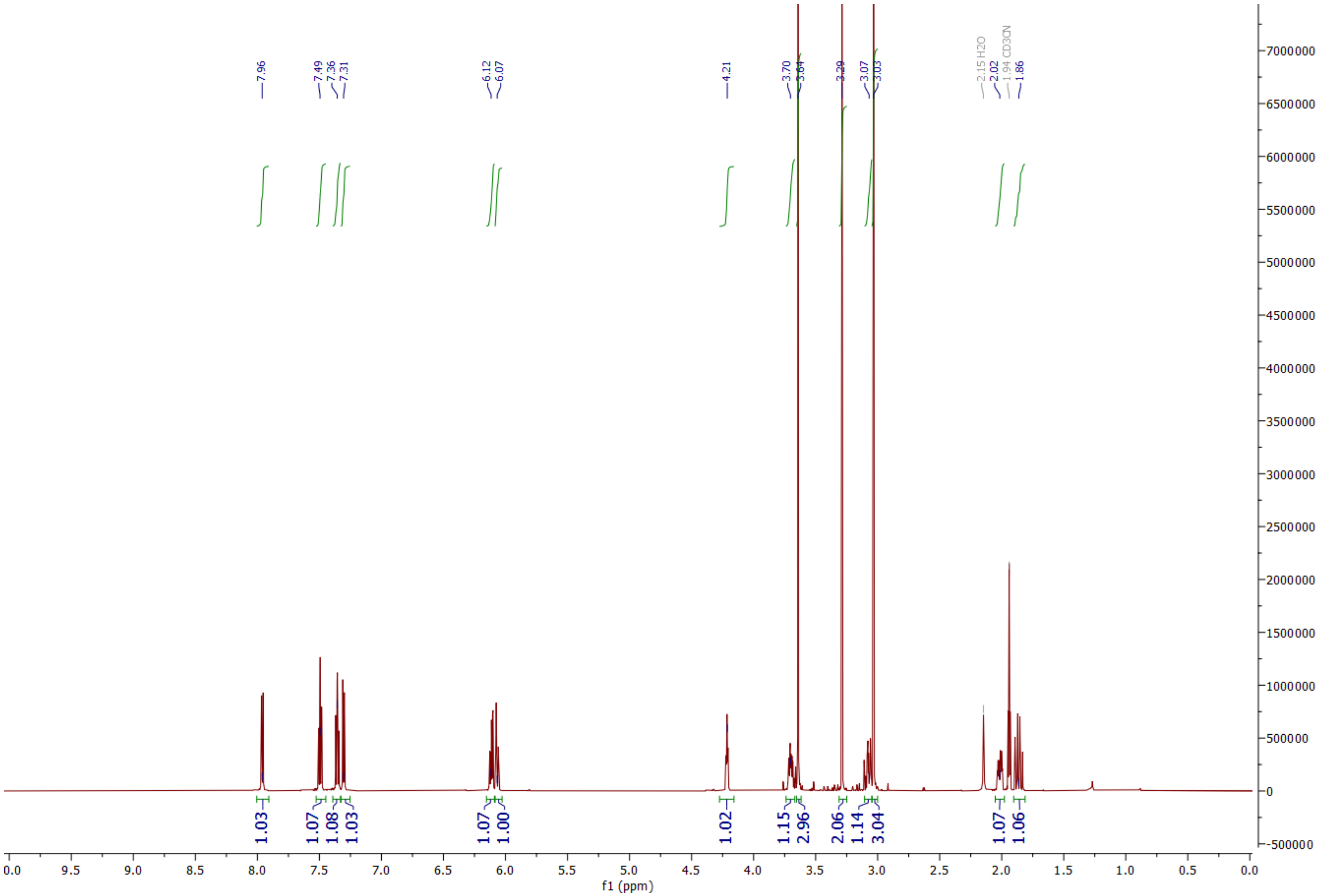

Supplementary Figure 46. ^1H NMR (CD_3CN) of Compound **30**.

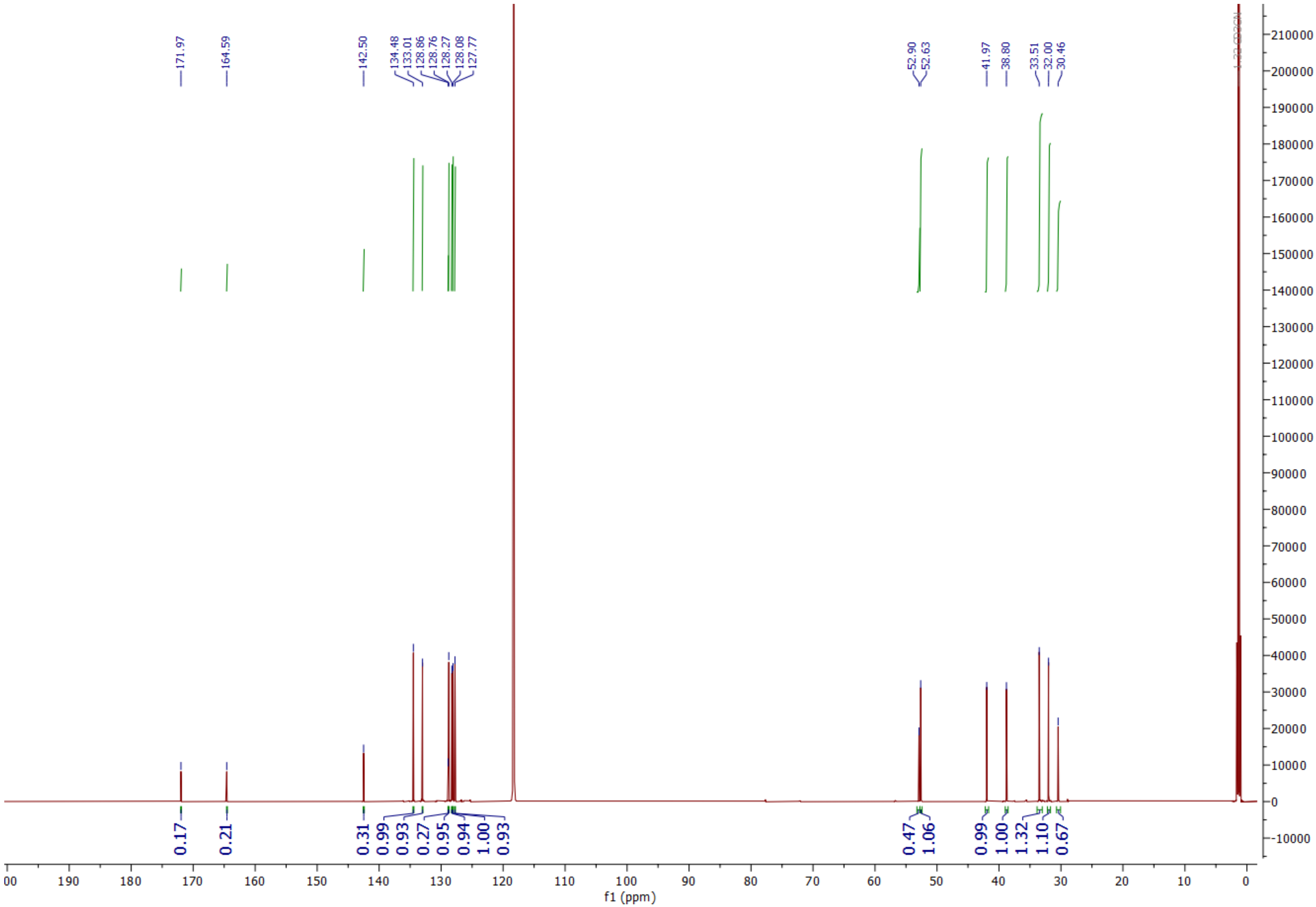

Supplementary Figure 47. ^{13}C NMR (CD_3CN) of Compound **30**.

Crystallographic Data

A single crystal of each of the molecules listed in the tables below was coated with Paratone oil and mounted on a MiTeGen micromount. Data for **2**, **9, 10, 15,** and **16** were collected on a Bruker D8 VENTURE dual wavelength Mo/Cu Kappa four-circle diffractometer using a PHOTON III detector. The diffractometer was equipped with an Oxford Cryostream 800Plus low temperature device and used Mo K_α radiation (λ = 0.71073Å) from an Incoatec IμS 3.0 microfocus sealed X-ray tube with a HELIOS double bounce multilayer mirror as monochromator. Data for **8** and **14** were collected on a Bruker Kappa APEXII Duo system equipped with an Oxford Cryostream 700Plus low temperature device and used a fine-focus sealed tube (Mo K_α, λ = 0.71073 Å) and a graphite monochromator.

Data collection and processing were done within the Bruker APEX3, APEX4 or APEX6 software suite.[21] All data were integrated with SAINT 8.40B using a narrow-frame algorithm and a Multi-Scan absorption correction using SADABS was applied.[22] Using Olex2 as a graphical interface, each structure was solved by dual methods with SHELXT and refined by full-matrix least-squares methods against F^2 using SHELXL.[23-25] All non-hydrogen atoms were refined with anisotropic displacement parameters. In all structures except **14**, the B-H hydrogen atoms as well as the hydrogen atoms on the carbons bound directly to W were located in the electron density map and refined isotropically. All other hydrogen atoms in all structures were placed in calculated positions using a riding model with their U_{iso} values constrained to 1.5 times the U_{eq} of their pivot atoms for terminal sp^3 carbon atoms and 1.2 times for all other carbon atoms. The CIF file for **16** was generated using FinalCif.[26]

For **8, 14** and **16**, the relative occupancy of the disordered atoms was freely refined. Constraints and restraints were used as needed on the anisotropic displacement parameters and/or bond lengths of the disordered atoms.

	**2**	**8**	**9**	**10**
CCDC number	2492652	2492653	2492654	2492655
Empirical formula	$C_{24}H_{37}BN_7O_5PS_2W$	$C_{32}H_{47}BN_7O6PS_2W$	$C_{28}H_{39}BN_7O_5PS_2W$	$C_{24}H_{37}BN_7O_6PS_2W$
Formula weight	793.35	915.81	843.41	809.35
Temp [K]	100(2)	100(2)	100(2)	100(2)
Cryst. system	monoclinic	monoclinic	monoclinic	triclinic
Space group	P 2_1/n	P 2_1/c	P 2_1/n	P -1
a [Å]	15.0164(6)	10.2478(4)	8.4835(3)	8.9515(14)
b [Å]	14.9620(6)	12.4081(4)	16.3613(9)	10.5541(18)
c [Å]	15.1244(6)	30.3304(12)	24.1419(11)	17.720(3)
α [°]	90	90	90	98.033(6)
β [°]	114.6340(10)	93.7510(10)	94.8460(10)	104.171(6)
γ [°]	90	90	90	100.091(6)
Volume [Å^3]	3088.8(2)	3848.4(2)	3338.9(3)	1568.5(5)
Z	4	4	4	2
ρ_{calc} [gcm^{-3}]	1.706	1.581	1.678	1.714
μ [mm^{-1}]	3.973	3.203	3.681	3.916
F(000)	1584	1849	1688	808
Crystal size [mm^3]	0.172 x 0.271 x 0.316	0.154 x 0.202 x 0.203	0.041 x 0.108 x 0.113	0.037 x 0.043 x 0.079

Crystal colour	yellow	colourless	colourless	colourless
Crystal shape	block	block	needle	block
λ [Å]	0.71073	0.71073	0.71073	0.71073
2Θ range [°]	4.02 to 56.62	2.70 to 56.60	4.20 to 52. 08	4.0 to 56.56
Index ranges	-20 ≤ h ≤ 19<br>-19 ≤ k ≤ 19<br>-20 ≤ l ≤ 20	-13 ≤ h ≤ 13<br>-16 ≤ k ≤ 13<br>-40 ≤ l ≤ 40	-10 ≤ h ≤ 10<br>-20 ≤ k ≤ 19<br>-29 ≤ l ≤ 28	-11 ≤ h ≤ 11<br>-14 ≤ k ≤ 14<br>-23 ≤ l ≤ 23
Reflections collected	41253	55444	38084	51573
Independent reflections	7611 [R_{int} = 0.0362]	9577 [R_{int} = 0.0412]	6586 [R_{int} = 0.0779]	7756 [R_{int} = 0.0623]
Completeness [%]	99.1	99.9	99.8	99.8
Data / Restraints / Parameters	7611 / 0 / 388	9577 / 788 / 653	6586 / 0 / 423	7756 / 0 / 397
Goodness-of-fit on F^2	1.096	1.146	1.006	1.027
Final *R* indexes [I≥2σ(I)]	0.0198	0.0312	0.0394	0.0246
Final *R* indexes [all data]	0.0461	0.0620	0.1021	0.0553
Largest peak/hole [eÅ^{-3}]	0.771 / -1.360	1.054 / -2.065	2.395 / -1.066	0.956 / -0.616

	**14**	**15**	**16**
CCDC number	2492656	2492657	2492658
Empirical formula	$C_{27}H_{41}BN_8O_3PSW$	$C_{29}H_{38}BN_8O_3PSW$	$C_{25}H_{36}BN_8O_5PSW$
Formula weight	783.37	804.36	786.31
Temp [K]	100(2)	100(2)	100(2)
Cryst. system	monoclinic	triclinic	monoclinic
Space group	I 2/a	P -1	P 2_1/c
a [Å]	15.9771(7)	7.7521(3)	14.5877(5)
b [Å]	12.0061(6)	12.3219(6)	15.3938(6)
c [Å]	34.1666(19)	17.9965(8)	14.2992(5)
α [°]	90	85.103(2)	90
β [°]	98.259(2)	82.564(2)	108.8350(10)
γ [°]	90	74.487(2)	90
Volume [Å^3]	6486.0(6)	1640.16(13)	3039.09(19)
Z	8	2	4
ρ_{calc} [gcm^{-3}]	1.604	1.629	1.719
μ [mm^{-1}]	3.717	3.678	3.972
F(000)	3144	804	1568
Crystal size [mm^3]	0.071 x 0.072 x 0.143	0.056 x 0.082 x 0.118	0.058×0.064×0.14
Crystal colour	colorless	colorless	colourless
Crystal shape	block	plate	plate
λ [Å]	0.71073	0.71073	0.71073
2Θ range [°]	3.60 to 51.48	4.22 to 52.10	3.96 to 55.10

Index ranges	-16 ≤ h ≤ 19<br>-14 ≤ k ≤ 13<br>-41 ≤ l ≤ 41	-9 ≤ h ≤ 9<br>-15 ≤ k ≤ 15<br>-22 ≤ l ≤ 22	−17 ≤ h ≤ 18<br>−20 ≤ k ≤ 20<br>−18 ≤ l ≤ 18
Reflections collected	36549	43694	51637
Independent reflections	6189 [R_{int} = 0.1009]	6475 [R_{int} = 0.0660]	7002<br>[R_{int} = 0.0661
Completeness [%]	99.8	99.9	100.0
Data / Restraints / Parameters	6189 / 242 / 477	6475 / 0 / 413	7002 / 25 / 441
Goodness-of-fit on F^2	0.855	1.130	1.010
Final *R* indexes [$I \geq 2\sigma(I)$]	0.0399	0.0379	0.0310
Final *R* indexes [all data]	0.0939	0.0797	0.0719
Largest peak/hole [$eÅ^{-3}$]	1.515 / -1.235	2.075 / -1.291	1.32/−0.92

References

[9] S. R. Simpson, P. Siano, D. J. Siela, L. A. Diment, B. C. Song, K. S. Westendorff, M. N. Ericson, K. D. Welch, D. A. Dickie and W. D. Harman, *Journal of the American Chemical Society* **2022**, *144*, 9489-9499.
[19] P. Siano, L. A. Diment, D. J. Siela, M. N. Ericson, M. McGraw, B. C. Song, B. F. Livaudais, S. R. Simpson, D. A. Dickie and W. D. Harman, *Organometallics* **2025**, *In Press*.
[21] in *APEX4; Apex5; Saint*, *Vol.* Bruker AXS, Inc., Madison, WI, USA, **2019**.
[22] L. Krause, R. Herbst-Irmer, G. M. Sheldrick and D. Stalke, *Journal of Applied Crystallography* **2015**, *48*, 3-10.
[23] O. V. Dolomanov, L. J. Bourhis, R. J. Gildea, J. A. K. Howard and H. Puschmann, *Journal of Applied Crystallography* **2009**, *42*, 339-341.
[24] G. Sheldrick, *Acta Crystallographica Section A: Foundations and Advances* **2015**, *71*, 3-8.
[25] G. M. Sheldrick, *Acta Crystallographica Section C: Structural Chemistry* **2015**, *71*, 3-8.
[26] D. Kratzert, "FinalCIF", https://dkratzert.de/finalcif.html, (accessed 2024-04-11).
